# Development of a pipeline for automated, high-throughput analysis of paraspeckle proteins reveals specific roles for importin α proteins

**DOI:** 10.1038/srep43323

**Published:** 2017-02-27

**Authors:** Andrew T. Major, Yoichi Miyamoto, Camden Y. Lo, David A. Jans, Kate L. Loveland

**Affiliations:** 1 Department of Anatomy and Developmental Biology, Monash University, Melbourne, Australia; 2 The ARC Centre of Excellence in Biotechnology and Development, Australia; 3 National Institutes of Biomedical Innovation, Health and Nutrition, Osaka, Japan; 4 Department of Biochemistry and Molecular Biology, Monash University, Melbourne, Australia; 5 Monash Micro Imaging Facility, Monash University, Melbourne, Australia; 6 Centre for Reproductive Health, Hudson Institute of Medical Research, Melbourne, Australia; 7 Department of Molecular and Translational Sciences, School of Clinical Sciences, Monash University, Melbourne, Australia

## Abstract

We developed a large-scale, unbiased analysis method to measure how functional variations in importin (IMP) α2, IMPα4 and IMPα6 each influence PSPC1 and SFPQ nuclear accumulation and their localization to paraspeckles. This addresses the hypothesis that individual IMP protein activities determine cargo nuclear access to influence cell fate outcomes. We previously demonstrated that modulating IMPα2 levels alters paraspeckle protein 1 (PSPC1) nuclear accumulation and affects its localization into a subnuclear domain that affects RNA metabolism and cell survival, the paraspeckle. An automated, high throughput, image analysis pipeline with customisable outputs was created using Imaris software coupled with Python and R scripts; this allowed non-subjective identification of nuclear foci, nuclei and cells. HeLa cells transfected to express exogenous full-length and transport-deficient IMPs were examined using SFPQ and PSPC1 as paraspeckle markers. Thousands of cells and >100,000 nuclear foci were analysed in samples with modulated IMPα functionality. This analysis scale enabled discrimination of significant differences between samples where paraspeckles inherently display broad biological variability. The relative abundance of paraspeckle cargo protein(s) and individual IMPs each influenced nuclear foci numbers and size. This method provides a generalizable high throughput analysis platform for investigating how regulated nuclear protein transport controls cellular activities.

DNA compartmentalization into the nucleus allows tight regulation of gene expression in eukaryotes. Transport between the nucleus and cytoplasm occurs solely through nuclear pore complexes (NPCs), which span the nuclear envelope. The NPC, constructed from about 30 different nucleoporin protein subunits, permits free bi-directional flow of ions and small macromolecules (<45 kDa) by passive diffusion, while larger protein cargos are transported by karyopherin family members, termed importins and exportins. For tight control of gene expression in the nucleus, the chromatin is arranged in specific chromosomal territories^1^, and several discrete and distinct sub-nuclear domains form to serve distinct functions^2^,^3^. Examples of such domains include the nuclear lamina^4^, the nucleolus^5^, Cajal bodies^6^, PML bodies^7^ and nuclear speckles^8^,^9^. Many protein components of sub-nuclear domains have been identified through co-localization studies and whole genome screening for GFP-fusion proteins which form intra-nuclear foci^10^. While not separated by membranes, the constituents of these domains differ and can be dynamically associated through exchange of components. In this study, we focus on understanding how regulated access to the nucleus affects formation of paraspeckles^11^.

Paraspeckles are a distinct nuclear domain built around the long non-coding RNA, nuclear paraspeckle assembly transcript 1 (NEAT1), formerly known as nuclear enriched abundant transcript 1. The NEAT1 transcript acts as a scaffold for recruitment and assembly of other paraspeckle components^12^,^13^,^14^,^15^,^16^. Three core *Drosophila* behaviour, human splicing (DBHS) paraspeckle proteins were initially identified^11^: paraspeckle protein 1 (PSPC1), splicing factor proline/glutamine rich (SFPQ, also named PSF and REP1) and the non-POU-domain-containing, octamer binding protein (NONO, also named NRB54 and P54NRB). The expanding number of proteins identified to localize to paraspeckles^10^,^17^ reflects data from a recent study mapping interactions between paraspeckle components^18^. Such evidence highlights the complex nature of this domain and may be used to understand how paraspeckles are assembled.

The cellular functions of paraspeckles are still being discerned. Thus far they have been shown to influence translation, through nuclear retention of A-to-I edited RNA transcripts^19^ and by the sequestration of proteins^20^. The finding that NEAT1^−/−^ mouse embryonic fibroblasts are more sensitive to proteasome inhibitor-induced apoptosis than their wildtype counterparts^20^ was interpreted as indicative of an influence of paraspeckles on cellular survival. This was supported by further evidence from various types of cancer, including breast^21^, colorectal^22^,^23^, glioma^24^, leukemia^25^,^26^, liver^27^, lung^28^,^29^,^30^,^31^ and prostate^32^ that correlate NEAT1 levels with either patient prognosis or cell behaviour. NEAT1^−/−^ mice lack paraspeckles^33^ but exhibit limited phenotypic defects restricted to mammary gland development^34^ and corpus luteum formation, resulting in female subfertility^35^. These contributions to normal and pathological cell activities highlight the value of learning how paraspeckle formation is governed.

Nucleocytoplasmic trafficking is of central importance to nuclear functions. Active nuclear import and export is facilitated by the karyopherin family proteins, comprised of importins and exportins which bind and transport proteins containing nuclear localization signals (NLSs) or nuclear export signals (NESs), respectively. Both importin αs (IMPαs) and importin βs (IMPβs) facilitate nuclear import. The mouse genome encodes six IMPαs and ~twenty karyopherin β family members, each with individual cargo-binding specificities^36^,^37^,^38^,^39^. In this study, we use the mouse nomenclature, in which each IMPα is a product of its corresponding *KPNA* gene (e.g. IMPα2 encoded by *KPNA2*), as previously^40^. IMPβs can form functional transport complexes in the cytoplasm by binding directly to an NLS-containing cargo protein, while IMPαs typically bind to both the cytoplasmic NLS-containing cargo and to IMPβ1, though an importin beta binding (IBB) domain. These complexes move through the NPC via transient interactions between IMPβs and the nucleoporins that line the NPC inner channel. Within the nucleus, high RAN-GTP levels effect cargo release by binding IMPβ to cause complex dissociation. Conversely, exportins require RAN-GTP to bind and transport NES-containing nuclear-localized cargoes; the export complex dissociates in the cytoplasm following RAN-GTP hydrolysis into RAN-GDP. In addition, some instances of cargo binding to the C-terminal acidic region of IMPα, rather than to its NLS binding groove, can mediate cargo retention in the nucleus^41^,^42^,^43^. Such retained cargoes are imported into the nucleus by different IMPs when there is a shift in the intracellular stoichiometry of IMPs^41^,^42^. These findings, from analysis of differentiating embryonic stem cells, demonstrate that regulated nucleocytoplasmic transport is a developmental gatekeeper. Spatiotemporal expression of individual importins and exportins appears to be tightly regulated during development and differentiation of embryonic stem cells^41^,^44^,^45^, muscle^46^,^47^ and germline cells^40^,^43^,^44^,^48^,^49^,^50^,^51^, although the mechanistic basis for this is largely unknown. Thus, an emerging concept in importin biology is that regulated synthesis of nucleocytoplasmic machinery mediates cellular differentiation, with individual IMPs controlling nuclear access of proteins to determine each cell’s transcriptional activity.

PSPC1 (a core DBHS paraspeckle protein) was identified as an IMPα2-interacting cargo protein in the mouse testis at the time of germline sex determination^49^. This binding relationship is highly likely to be of functional relevance for spermatocytes (meiotic germ cells) and spermatids (haploid germ cells) in the adult testis, as each contains abundant PSPC1^52^ but different amounts of each IMPα2, IMPα3 and IMPα4^43^. We hypothesized that changes in the stoichiometry of individual IMPα proteins are important for cellular differentiation, including during spermatogenesis, and set out to devise a strategy to address this. Our previous work employed HeLa cells, which have been widely used to study the functional outcomes of manipulating importin levels and functionality. By detecting endogenous PSPC1 using immunofluorescence, we observed that IMPα2 levels directly relate to the number of nuclear foci^49^. This analysis, performed using manual cell cropping from confocal z-series images, demonstrated that per cell paraspeckle numbers vary within an apparently homogenous culture, with typically between 5 and 20 foci present per nucleus^53^. This variation in endogenous paraspeckle numbers limited our capacity to discern significantly different outcomes against the background of normal biological variation. The present study provides a significant advance in which we develop and apply an automated, high throughput image analysis pipeline to quantify paraspeckles in cells with altered IMPα protein levels and functionality. This pipeline was used to rigorously analyse large numbers of cells, allowing us to measure variability in nuclear foci numbers, nuclear foci parameters (size, intensity of staining, etc.) and nuclear accumulation (nuclear/cytoplasmic ratios) of two core paraspeckle markers (PSPC1 [endogenous and exogenous] and SFPQ [endogenous only]). These parameters were investigated in response to modulating IMPα2, IMPα4 and IMPα6, corresponding to one representative from each of the three IMPα structural clades^39^,^48^,^54^. The results of this analysis demonstrate how the regulation of individual IMPαs alters core paraspeckle protein delivery to paraspeckles, providing the first high-throughput functional analysis of differences in importin protein levels within a single cell population.

## Results

### A high-throughput semi-automated image analysis pipeline developed to identify cells, nuclei and foci

**Figure 1:**
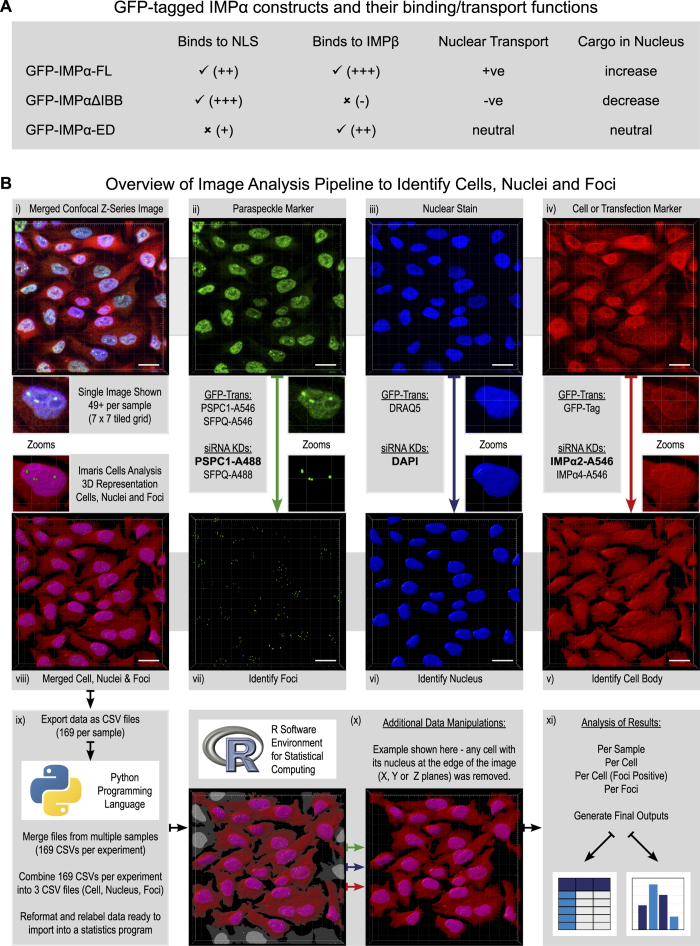
Overview of experimental and analytical approaches used to identify changes to subnuclear foci in response to modulating the cells nuclear transport capacity. Using either transient transfection with plasmids encoding GFP-tagged IMPα2/α4/α6 variants or siRNA knockdown of IMPα2/α4 the capacity of IMPαs to modulate delivery of PSPC1/SFPQ into the nucleus/paraspeckles was investigated. All images are Z-series captured via confocal laser scanning microscopy, scale bars represent 20 μm. (**A**) GFP-tagged IMPα isoforms and their functional properties. Binding is indicated as true (✓) or false (✗), with an indication of binding strength (+/−). (**B**) Overview of image analysis pipeline. From this example merged z-series confocal image (**Bi**), the immunofluorescent signal for endogenous PSPC1 (**Bii**) was used to identify foci (**Bvii**), the nuclear marker DAPI (**Biii**) was used to identify the nuclei (**Bvi**) and the immunofluorescent signal for endogenous IMPα2 (**Biv**) was used to identify the cell body (**Bv**). The other options for paraspeckle marker, nuclear stain and cell or transfection marker for the GFP-tagged IMPα experiments (GFP-Trans) or siRNA knock down experiments (siRNA KDs) are listed. The full 3D reconstruction of cells, their nuclei and foci (**Bviii**) is shown for this example image but it should be noted that each sample in an experiment has 49 + of these images. Data about these cells, their nuclei and foci were exported from Imaris in CSV file formats and reorganised ready for statistical analysis using custom python programming language scripts (**Bix**). Additional data manipulations were performed on a per experiment basis (as detailed for each) using custom R scripts (**Bx**), before final statistical analysis and outputs were generated using the R software environment for Statistical Computing (**Bxi**). The Python logo is a trademark of the Python Software Foundation (https://www.python.org; v2.7). The R logo (https://www.r-project.org/logo/) is licensed under CC BY-SA 4.0; the license terms can be found on the following link: (https://creativecommons.org/licenses/by-sa/4.0/).

To investigate how different IMPαs could modulate PSPC1 delivery into the nucleus and into paraspeckles, expression levels and transporter functionality of individual IMPα within HeLa cells were modulated by two independent approaches. In one, transient transfection was used to introduce expression constructs encoding green fluorescent protein (GFP)-tagged isoforms of IMPα2, IMPα4 and IMPα6, corresponding to either full length or truncated ΔIBB variants (summarized in Fig. 1A). Transient over-expression of a GFP-tagged full-length IMPα protein will increase nuclear accumulation of its cargoes. In contrast, IMPαΔIBB isoforms, which lack the importin beta binding (IBB) domain, exhibit a dominant negative effect on cargo accumulation because these still bind cargo proteins but cannot bind IMPβ1 to form a functional transport complex^55^; the resulting competitive binding will diminish cargo availability for binding endogenous IMPα and thereby reduce cargo nuclear accumulation. For IMPα2, an additional control construct containing two point mutations in the NLS binding groove (lysine replacement at aa192 and arginine at aa396) was used (GFP-IMPα2-ED). These mutations significantly reduce cargo binding^55^,^56^, but have little or no effect on endogenous IMPα2 cargo nuclear transport; this isoform serves as a control for non-nuclear transport-related effects arising from GFP-IMPα2 over-expression. The binding capacity and predicted nuclear transport outcomes from transfection with each of IMPα isoform construct are summarized in Fig. 1A. Other control samples included GFP alone, mock-transfected and not-transfected cells. The second approach used to modulate IMPα levels in HeLa cells was siRNA knockdown, targeting IMPα2 and IMPα4.

In all experiments, tiled confocal z-series images were collected for 3D visualisation and analysis, allowing the full volume of numerous (between 143 and 813) individual cells to be analysed in each sample (Pipeline outlined in Fig. 1B). Briefly, using Imaris software, cells, nuclei and PSPC1/SFPQ nuclear foci were identified. Results were exported from Imaris in CSV formats, processed and compiled into a compatible format using a series of custom Python scripts and then imported into the ‘R environment for statistical computing’ for analysis. This approach facilitated analysis of thousands of cells and quantification of hundreds of thousands of nuclear foci in a consistent and non-subjective manner. All raw data, exported from Imaris along with the custom python, R and shell scripts which compile and analyse these data, are provided in Supplementary Dataset SD1.

Cell gating was initially set to capture a very low GFP signal level, corresponding to the auto-fluorescence signal level. In this way, the cytoplasm and nucleus of every cell was identified, regardless of whether it was transfected or not. This approach removed the need to use an additional cell body marker, maximizing available fluorescence channels and minimising photo-damage by reducing laser exposure. To ensure that only transfected cells were analysed, a final mean GFP intensity threshold per cell (higher expression level of GFP) was later applied to the data using the R environment for statistical computing (Fig. 1Bx). This allowed the GFP thresholding to be applied to all test and control samples simultaneously, with adjustments made to identify a threshold where the detected cell number approached zero in the control samples. GFP thresholding was also selectively withheld from the control samples to extend analyses to non-transfected and mock-transfected cells using the same base parameters for cell, nuclei and foci detection. Overall, this analysis approach enabled accurate cytoplasmic identification of cells that had relatively low GFP-IMPα expression levels to achieve comprehensive measurements for all cells within each sample.

The nuclear detection threshold was set to ensure the nucleus was identified even in cells with a low level nuclear marker signal. Although this could slightly inflate the detected volume of each nucleus, this approach was chosen to avoid missing parts of some nuclei which could underestimate nuclear foci numbers. Using the R environment for statistical computing, nuclei on the edge of an image in the X, Y or Z image planes, and therefore likely to be incomplete nuclei, were excluded from the data sets. Subsequently, all cells without nuclei were also removed from the data sets (see Fig. 1Bx).

In the GFP-IMPα transient transfection study, the non-transfected (Not-Trans-C), mock-transfected (Mock-C) and GFP-transfected (GFP) control sample parameters for nuclear foci varied (Supplementary Tables S5, S6 and S7). We hypothesize that these differences reflect the physiological state of individual cells from each group in regard to cell cycle or local microenvironment differences at the time of sampling. This would be consistent with reported paraspeckle roles, but spotlights the paucity of knowledge about the inherent variability of paraspeckles within a population, and whether these are dynamically modulated within a cell in response to particular conditions. These results lead us to conclude that comparing outcomes within a single IMPα subtype, in which either cargo or IMPβ1 binding has been manipulated, is appropriate, while comparing between different IMPα subtypes should be undertaken cautiously, and with this information in mind.

### Functional IMPα protein levels determine endogenous PSPC1 localization to paraspeckles

**Figure 2:**
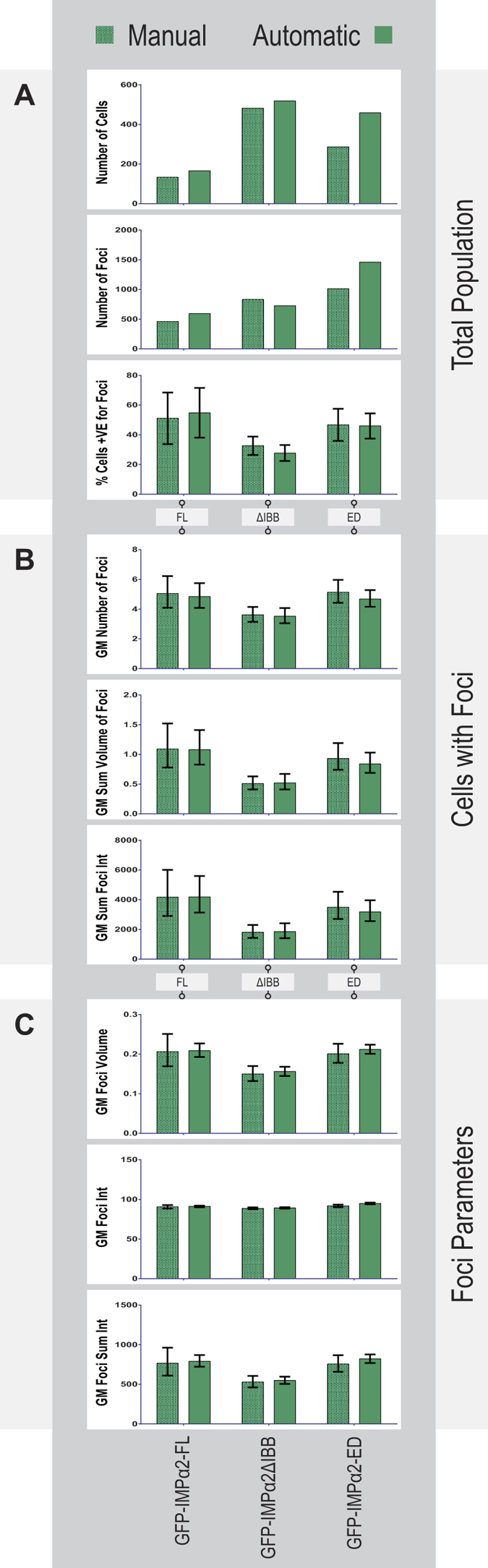
Comparing outcomes from manual versus automatic cell detection methods. Comparative outcomes of modulating functional IMPα2 levels on PSPC1 nuclear transport and paraspeckle localization in HeLa cells. Cells were transiently transfected with constructs encoding GFP-tagged IMPα2 variants as indicated (see Fig. 1A for predicted function). Paraspeckles were identified using indirect immunofluorescence to detect endogenous PSPC1. These measures were assessed within groups for the entire cell population (**A**), per foci positive cell populations (**B**) or on a per foci basis (**C**). Most measures are shown as geometric means (GM); error bars represent 95% confidence intervals.

**Figure 3:**
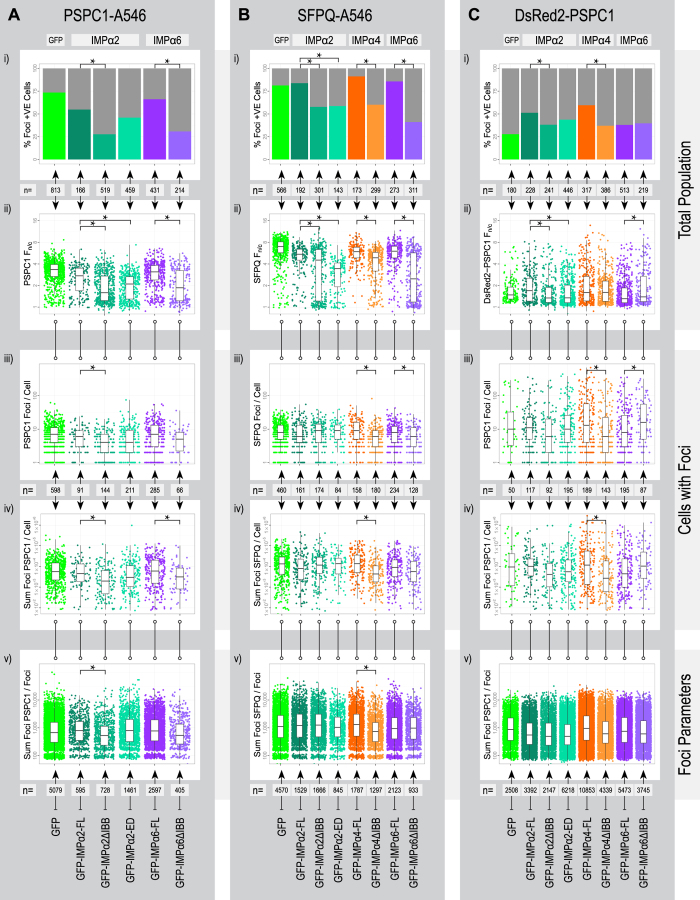
Outcomes of modulating functional IMPα2/α4/α6 levels on paraspeckle marker (endogenous PSPC1/SFPQ or exogenous DsRed2-PSPC1) nuclear transport and paraspeckle localization in HeLa cells. HeLa cells were transiently transfected with constructs encoding GFP-tagged IMPα2/α4/α6 variants (see Fig. 1A for predicted function) as indicated, with labels at the bottom of each panel and consistent colors used throughout. Paraspeckles were assessed within experimental groups using indirect immunofluorescence with an Alexa Fluor 546 (A546) secondary antibody to detect endogenous PSPC1 (**A**), using indirect immunofluorescence with an Alexa Fluor 546 (A546) secondary antibody to detect endogenous SFPQ (**B**) or through exogenous PSPC1 by co-transfecting with a plasmid encoding DsRed2-PSPC1 (**C**). After analysis pipeline as outlined in Fig. 1B, the primary measures are presented as bar graphs and scatter plots with overlaid box plots indicating the mean and interquartile ranges for each experimental group. Samples with statistically significant differences within IMPα groups are indicated (*). The primary measures include the percentage of foci positive cells (i), the ratio fluorescent signal within the nucleus and cytoplasm (F_n/c_) for the paraspeckle marker used (ii), the number of foci detected per cell (iii), the sum foci associated fluorescent signal per cell for the paraspeckle marker used (iv) and the sum foci associated fluorescent signal per foci for the paraspeckle marker used (v). These measures were assessed within groups per entire cell population (i, ii), per foci positive cell populations (iii, iv) or on a per foci basis (v).

To assess the accuracy of the automated analysis pipeline, we initially compared its outcomes with those from our previous analysis using manual selection of individual cells^49^. HeLa cells were transiently transfected to express GFP-tagged IMPα2 or IMPα6, as each binds PSPC1 in a yeast two hybrid system and in an ELISA-based importin binding assay^49^. During cell/nucleus/foci detection in Imaris, nuclear PSPC1 foci were identified using the immunofluorescent signal for endogenous PSPC1 with parameters matching our previous study^49^ to allow a direct comparison. All IMPα2 samples produced results similar to those previously reported^49^, with GFP-IMPα2-ED control values intermediate to those obtained with the other two IMPα2 isoforms (summary comparison in Fig. 2; detailed comparison in Supplementary Table S1). This congruency demonstrates that automated detection of cells and nuclei is of comparable accuracy to the laborious manual cell image cropping. For all paraspeckle-related endpoints, all but one of the GFP-IMPα2ΔIBB sample values were significantly reduced relative to the GFP-IMPα2-FL values (Table 1 and Fig. 3A). The one exception was the geometric mean (GM) PSPC1 voxel intensity (per foci), which was not significantly reduced (Table 1 L).

To interrogate nuclear accumulation, the mean of the fluorescent signal in the nucleus (F_n_) and cytoplasm (F_c_) was converted to a ratio (F_n/c_) for each cell^44^,^57^. Mean PSPC1 F_n/c_ values for all IMPα2 samples increased with increasing IMPα2 functionality as expected (Table 1E and Fig. 3Aii; ΔIBB [lowest function]: 2.04; ED: 2.08; FL [highest function]: 2.69). The other GFP-IMPα2-FL sample parameters were unchanged or slightly increased compared with those from the GFP-IMPα2-ED control. The only significantly different result was the PSPC1 F_n/c_ value, indicating that the FL isoform significantly enhances PSPC1 nuclear accumulation.

Our previous demonstration of IMPα6 binding to PSPC1 in yeast two hybrid and ELISA assays was extended here by measuring paraspeckle numbers and size in HeLa cells relative to IMPα6 functionality. Significant differences in several parameters were recorded when comparing the FL and ΔIBB variants of IMPα6. The ΔIBB variant exhibited a lower proportion of foci-positive cells, reduced nuclear accumulation of PSPC1 (PSPC1 F_n/c_), a lower total volume of foci per cell and a reduction in the total signal from PSPCI-foci per cell when compared to the FL isoform (Table 1 and Fig. 3A). Although the number of foci measured per cell was reduced in the ΔIBB sample (FL:6.00; ΔIBB:4.41), this outcome did not reach significance, which most likely reflects the low proportion of cells containing nuclear foci in these samples (54.8% in FL [n = 91]; 30.8% in ΔIBB [n = 66]). This finding indicates that changing levels of IMPα6 will also influence PSPC1 nuclear accumulation and the characteristics of PSPC1-positive nuclear foci, as recorded for IMPα2.

These data demonstrate IMPα2 and IMPα6 can each modulate endogenous PSPC1 nuclear accumulation and localization to paraspeckles. In addition, the direct comparison to our previous work with IMPα2 validates the automated analysis pipeline as an effective tool for detecting these outcomes.

### Functional IMPα protein levels modulates endogenous SFPQ localization to paraspeckles

To determine if changes in IMPα expression levels that altered PSPC1 nuclear accumulation and localization into paraspeckles also affected another core DBHS paraspeckle marker, we examined endogenous SFPQ in HeLa cells transiently transfected to express GFP-tagged IMPα constructs. IMPα2 variants influence SFPQ localization to nuclear foci in a manner similar to that recorded for PSPC1 localization (Table 2 and Fig. 3B). The percentage of cells containing SFPQ nuclear foci is greatly increased in the IMPα2-FL group (83.9%), and slightly decreased in the IMPα2ΔIBB group (57.8%), compared to the IMPα2-ED control sample (58.7%); ED and ΔIBB values are each significantly different (p = 0.0000) from the FL outcome (Table 2D and Fig. 3Bi). The F_n/c_ for SFPQ was significantly reduced (p = 0.0000) in the ΔIBB (3.21) and ED (2.80) groups in comparison to IMPα2-FL (4.75); the odds ratios when compared to the FL set to 1.0 are 0.675 for ΔIBB and 0.590 for ED (Table 2E and Fig. 3Bii). No other paraspeckle parameters displayed statistically significant differences. These outcomes suggest that SFPQ transport is affected by IMPα2 functionality, but its relationship to paraspeckles is not.

Transfection with IMPα4 isoforms resulted in remarkable and significant differences measured between IMPα4-FL and IMPα4ΔIBB samples, across the population, cell and individual foci parameters (Table 2 and Fig. 3B). Many parameters showed a higher value in the IMPα4-FL sample, including: the percentage of cells with SFPQ nuclear foci (FL:91.3%; ΔIBB:60.2%), the number of foci per cell (FL:8.13; ΔIBB:5.14), the average volume of foci (FL:0.320 μm^3^; ΔIBB:0.202 μm^3^) and the sum of the SFPQ staining intensity per foci (FL:1204; ΔIBB:728). This demonstrates that IMPα4 functionality can determine SFPQ localization to nuclear foci.

The IMPα6-FL group contained a significantly higher percentage of cells with nuclear foci (85.7%) than did the IMPα6ΔIBB group (41.2%; p = 0.0000; Table 2D and Fig. 3Bi). A significantly greater F_n/c_ per cell for SFPQ (FL:5.32; ΔIBB:2.66, p = 0.0000), and number of nuclear foci per cell (FL:6.85; ΔIBB:5.25, p = 0.0046) was measured within the IMPα6-FL group compared to the IMPα6ΔIBB group (Table 2E,H and Fig. 3Bi,Biii). The absence of other statistically significant differences indicates that, while the number of paraspeckles per cell differs depending on IMPα6 functionality, the parameters of individual foci (volumes and SFPQ) do not.

These results show that changes in the functional levels of individual IMPα influence multiple paraspeckle parameters, including the localization of specific, key components. Thus the relative intracellular abundance of individual importins, and their availability for cargo binding, will affect paraspeckle formation.

### Functional IMPα protein levels modulate exogenous dsRed2-PSPC1 localization to paraspeckles

We predicted that the changing levels of specific cargos would also alter how IMPαs influence paraspeckle parameters. To test the impact of IMPα functionality when cargo is elevated, exogenous PSPC1 (dsRed2-PSPC1) and GFP-tagged IMPα constructs were co-transfected into HeLa cells.

A greater but not significantly different (p = 0.0614) proportion of cells contained PSPC1 foci in the IMPα2-FL (51.3%) compared to IMPα2-ED samples (43.7%), while this was significantly lower in the IMPα2ΔIBB group (38.2%; p = 0.0042, compared to FL; Table 3D and Fig. 3Ci). Only the DsRed2-PSPC1 F_n/c_ value was statistically significantly higher in the FL sample relative to the IMPα2-ED (p = 0.0001) and ΔIBB (p = 0.0019) groups (FL:1.86; ΔIBB:1.62; ED:1.60; Table 3E and Fig. 3Cii). We interpret this as indicating that cells have an increased capacity for cargo transport (above endogenous levels) in the presence of increased levels of transport-competent IMPα2. A direct comparison of exogenous versus endogenous PSPC1 data is shown in Supplementary Table S1. As expected, samples containing exogenous PSPC1 have a more and larger nuclear foci containing more PSPC1, relative to samples containing only endogenous PSPC1.

No significant difference in the percentage of cells with nuclear DsRed2-PSPC1 foci was recorded between samples expressing FL (38%) or ΔIBB (39.7%) IMPα6 variants. However, the F_n/c_ (FL:1.56; ΔIBB:1.86, p = 0.0000) and number of paraspeckles per cell (FL:9.04; ΔIBB:15.58, p = 0.0063) differs significantly. Unexpectedly, the ΔIBB construct displays higher F_n/c_ and paraspeckle number per cell, and the average foci volume per cell trends higher (FL:1.29; ΔIBB:2.59, p = 0.0080, not considered significant with Bonferroni correction).

Analysis of IMPα4 variants revealed significant effects on several outcomes measured for exogenous PSPC1, but only when considered at the level of individual cells. The IMPα4-FL values were higher than ΔIBB levels for: percentage of cells with nuclear foci (FL:59.6%; ΔIBB:37%, p = 0.0000), number of foci per cell (FL:14.94; ΔIBB:7.78, p = 0.0001) and cumulative volume of foci (FL:2.48; ΔIBB:1.00, p = 0.0001). No significant reduction in DsRed2-PSPC1 F_n/c_ was recorded, which was different than the significant decreases observed with the transport-deficient isoforms of either IMPα2 or IMPα6. This suggests transport of exogenous PSPC1 is not regulated by IMPα4 levels, but that IMPα4 does influence PSPC1 localization into paraspeckles. This aligns with ELISA-based assays that measured IMPα4 binding to PSPC1 only at high IMPα4 concentrations, with weaker binding than was recorded for IMPα2 or IMPα6^49^.

### Expression levels of IMPα2 or IMPα4 correlate with PSPC1 nucleocytoplasmic distribution

As an alternative approach to measuring the outcomes of modulating importin function, IMPα2 or IMPα4 knockdown by targeted siRNA was followed by simultaneous detection of either endogenous PSPC1 or SFPQ (each in duplicate experiments) and the relevant IMPα by indirect immunofluorescence (Supplementary Tables S8–S15). The mean intensity of IMPα2 per cell on a population basis was reduced across the four experimental samples by introduction of siRNA targeting IMPα2 when compared to the scrambled siRNA control. The IMPα2 siRNA versus control signals were 0.43 and 0.59 for samples in which PSPC1 was detected, and 0.65 and 0.87 for SFPQ samples (calculated from values in Supplementary Tables S8–S15), demonstrating effective IMPα2-targeting by these siRNAs. This was confirmed by Western blot with cell lysates (data not shown). Although the attempted siRNA knockdown of IMPα4 was not consistently effective, these samples provided cell populations with a range of IMPα4 levels that were used in subsequent analyses.

A faster approach for image acquisition was trialled, using a resonance scanner to capture confocal z-series images for these samples. While scanning times were reduced to approximately 25% (from 32 days with galvo-scan imaging, to 8 days using the resonance scanner), reduced image quality made robust identification of foci impossible. As a consequence, outputs requiring foci detection are not presented or discussed for these experiments. F_n/c_ measurements, which require only detection of the cell nucleus and cytoplasm, were reliably determined from these images, allowing the influence of each IMPα on PSPC1 or SFPQ nuclear accumulation to be determined following resonance scanning. The PSPC1 F_n/c_ values in the IMPα2 siRNA knockdown samples were reduced to ~80% of their scrambled counterparts (PSPC1: 0.78 and 0.80; SFPQ: 0.81 and 0.94; calculated from values in Supplementary Tables S8–S15).

**Figure 4:**
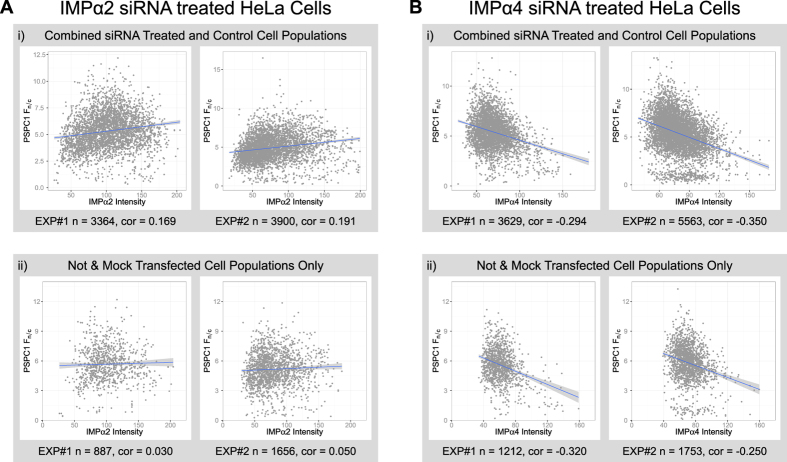
PSPC1 nuclear accumulation correlates with cellular IMPα levels in HeLa cells. Population wide correlations were observed, where treatment groups (Not transfected, mock transfected, scrambled-10, scrambled-25, IMPα-10, IMPα-25) within two independent experiments (EXP#1 and EXP#2) were pooled and the total number of cells (n) were used to produce correlation coefficients (c) between cellular IMPα intensity and the ratio of PSCP1 nuclear to cytoplasmic intensity (F_n/c_).

To explore the flexibility and power of creating hierarchically linked outputs that describe multiple aspects of each cell, a different analysis approach was applied. Instead of making comparisons between siRNA knockdown groups, these outputs based on fluorescence signal were considered across the whole population of cells, regardless of treatment group. Correlations between PSPC1 F_n/c_ and the IMPα signal within each cell are presented in Fig. 4. The upward sloping line in Fig. 4Ai indicates that, as IMPα2 levels increase within cells, PSPC1 F_n/c_ values also increase (correlation coefficients of 0.169 and 0.191 obtained for two independent experiments). The IMPα4 samples generated the opposite result, showing a reciprocal relationship between PSPC1 F_n/c_ and IMPα4 levels (downwards sloping trend line, Fig. 4Bi; correlation coefficients of −0.294 and −0.350 for each of two experiments). These results provide an additional indication that IMPα2 is a nuclear transporter for endogenous PSPC1 in HeLa cells, and they suggest that IMPα4 is not. An alternative explanation for the lack of correlation with IMPα4 levels may be that the expression across the cell population is relatively low and uniform, yielding a small dynamic range of signal. A similar analysis for SFPQ did not yield consistent results between replicates (Supplementary Figure S1); we interpret this to indicate IMPα2 and IMPα4 are not the only transporters for this paraspeckle protein because knockdown did not alter SFPQ distribution, while over-expression of IMPαs did (Fig. 3).

Finally, non- and mock- transfected cell groups alone were examined to study cell populations with a broad range of endogenous IMPα expression in the absence of any importin manipulations (Fig. 4Aii and Bii). The overall trends observed were similar to those obtained from the complete set of siRNA knockdown samples (Fig. 4Ai and Bi). This result confirms the value of previous studies, in which F_n/c_ values correlate with IMP-based transport outcomes. Most importantly, the result of analyzing cells which have not been transfected demonstrates how application of a high throughput image analysis system can yield sophisticated and functionally relevant outcomes using only indirect immunofluorescence to detect endogenous cargo(s) and IMP proteins. This provides an exciting avenue for studying nucleocytoplasmic transport within intact tissues, by examining developmental systems in the absence of manipulations.

## Discussion

Development and application of an automated image analysis pipeline enabled the rigorous interrogation of how IMPα functionality affects paraspeckle number and size. Imaris software allowed non-subjective and relatively fast batch-processing of hundreds of 3D images to identify cells, nuclei and foci. This was linked into an analysis pipeline using python and R scripts that extended the flexibility of data manipulation and provided access to a diversity of statistical analysis tools and graphical outputs. To also investigate nuclear transport of two key paraspeckle components, PSPC1 and PSF, distinct from their localization for nuclear foci formation, the pipeline calculated the ratio between the fluorescent nuclear and cytoplasmic signals for these proteins (F_n/c_). Manual F_n/c_ measurement is very time-consuming, potentially subjective, and cannot be accurately applied to samples with uneven fluorescent signals that can arise from protein localization to subcellular structures, such as paraspeckles. Because our approach segments the entire nucleus and cytoplasm in 3D, brighter or darker structures in either compartment are accounted for in the measured means. Once appropriate cell/nucleus/vesicle detection parameters have been determined, many images/cells can be analysed easily, with high quality 3D image acquisition times then becoming the primary limiting factor for extending cell analysis numbers. At present, achieving the correct balance between lengthy imaging times and final image quality is a challenging aspect of such high throughput experiments. We trialled the use of resonance confocal scanning to accelerate image acquisition for the IMPα siRNA experiments. The associated loss of image quality made this approach inappropriate for sub-organelle feature scale quantification, however analyses of organelle feature scales (such as F_n/c_ outcomes) for whole cell populations provided meaningful measurement of endogenous nucleocytoplasmic transport activity.

**Figure 5:**
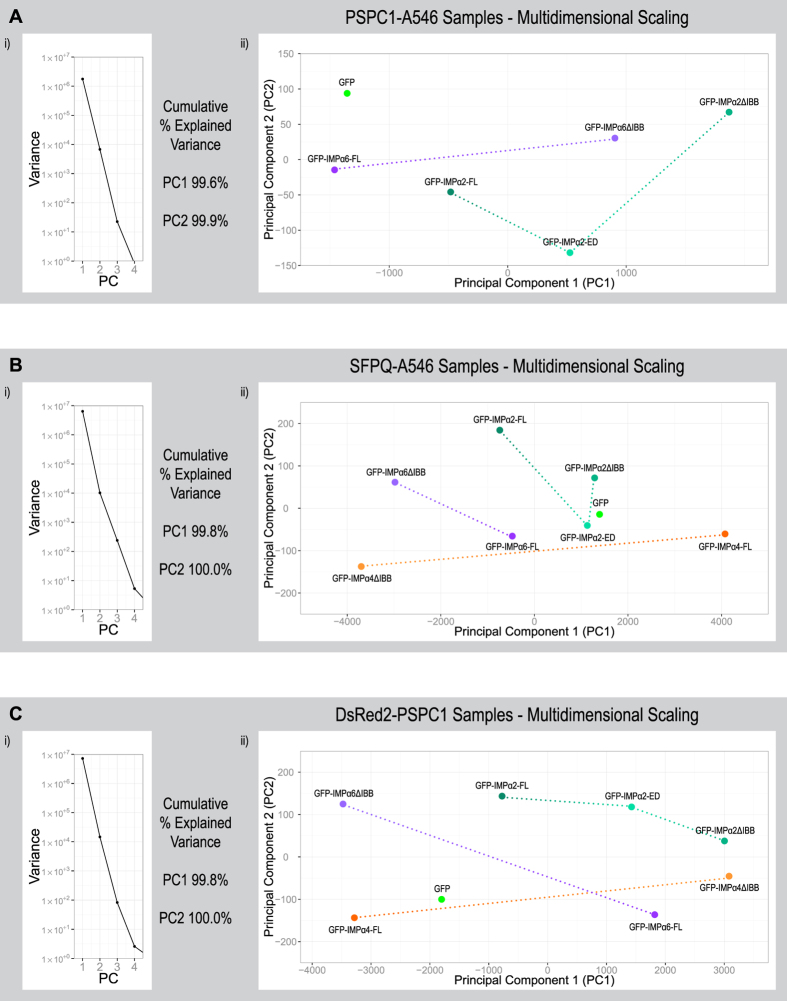
Modulating functional IMPα2/α4/α6 levels affected a plethora measurable paraspeckle related outcomes that can be simultaneously visualised using principal component analysis (PCA). The results of transiently transfecting HeLa cells with constructs encoding GFP-tagged IMPα2/α4/α6 variants (Tables 1, 2 and 3 and Fig. 2) were used to perform PCA, allowing simultaneous comparisons of multiple parameters and revealing strong patterns between groups. In each experiment PC1 explains >99% of the variance across all parameter and therefore the distances between groups across the X axis (PC1) should be considered as the primary delineator. Paraspeckles were assessed within experimental groups using indirect immunofluorescence with an Alexa Fluor 546 (A546) secondary antibody to detect endogenous PSPC1 (**A**), using indirect immunofluorescence with an Alexa Fluor 546 (A546) secondary antibody to detect endogenous SFPQ (**B**) or through exogenous PSPC1 by co-transfecting with a plasmid encoding DsRed2-PSPC1 (**C**). Parameters used to compare the geometric means of groups within experiments using a specific paraspeckle marker (PSM; A:PSPC1, B:SFPQ, C:DsRed2-PSPC1) were “% cells positive for foci”, “cytoplasmic PSM intensity”, “nuclear PSM intensity”, “PSM F_n/c_ per cell”, “PSM intensity per cell”, “number of nuclear foci per cell”, “sum volume of nuclear foci per cell”, “sum nuclear foci PSM intensity per cell”, “nuclear foci volume”, “nuclear foci PSM intensity” and “sum nuclear foci PSM intensity”.

Using an automated high-throughput image analysis pipeline can generate an overwhelming amount of data across multiple parameters in a relatively short time frame; sifting through this to identify the meaningful results can be both challenging and tedious. To help solve this problem we included principal component analysis (PCA) as part of the analysis pipeline. Through PCA, multiple parameters across groups of each experiment were condensed into two principal components, allowing a simple 2D relationship across all included parameters to be generated (Fig. 5). In addition to providing an accessible summary of the results, PCA also helps identify key outcomes during the initial stages of data analysis, thereby providing strategic directions for subsequent data interrogation.

The results in this study collectively demonstrate that modulating functional levels of IMPα2, IMPα4 and IMPα6 will impact nuclear import and delivery of PSPC1 and SFPQ to nuclear paraspeckles, and also provides evidence that the relative abundance of individual IMPαs and the cargo paraspeckle protein(s) influences these outcomes. In addition to reinforcing the knowledge that PSPC1 is a transport cargo of IMPα2^49^, the manipulation of IMPα6 functionality in HeLa cells provides new evidence that this importin can also effect nuclear transport of this core paraspeckle protein. The transport role of IMPα4 is less clear, because the F_n/c_ of over-expressed PSPC1 was not significantly different between samples co-transfection with either fully functional (FL) or transport-deficient (ΔIBB) isoforms. This contrasts with IMPα2 and IMPα6, for which the ΔIBB variants had lower nuclear-localized PSPC1 relative to FL counterparts. The endogenous SFPQ dataset (Fig. 3B and Table 2) differs, with IMPα2, IMPα4 and IMPα6 isoforms each influencing nuclear accumulation (F_n/c_) and the percentage of foci-positive cells. Given that SFPQ has not been documented as an IMPα cargo, further investigation would be required to determine if these effects are a result of direct or indirect actions of IMPα. Importantly, all SFPQ paraspeckle parameters are significantly influenced by the IMPα4 isoform (but not by IMPα2 and IMPα6, for which no individual foci parameters were affected). This suggests a unique functional relationship exists between SFPQ and IMPα4 that facilitates SFPQ nuclear import and paraspeckle localization. IMPα4 over-expression does not increase exogenous PSPC1 nuclear accumulation, but increases DsRed2-PSPC1 nuclear foci numbers, indicative of higher paraspeckle numbers in each cell. We hypothesize that IMPα4 over-expression mediates paraspeckle enlargement, potentially through the elevation of SFPQ in paraspeckles, thereby stabilizing NEAT1 RNA^17^, and enabling higher levels of PSPC1 recruitment and accumulation into paraspeckles.

These findings will be of particular importance in developmental systems in which IMPα levels are dynamically regulated and paraspeckles or components thereof are also present. We previously showed that IMPα2 expression peaks in the embryonic mouse testis (E12.5) and the adult mouse testis at developmental stages overlapping with PSPC1 expression^49^. NEAT1 transcripts also increase during muscle differentiation from myoblasts into myotubes, when paraspeckles are documented as enlarged and present in greater numbers^12^. This observation is interesting given that regulated expression of the nuclear transport machinery has also been implicated in muscle differentiation, with increasing IMPα2 linked to myoblast proliferation, myocyte migration and myotube size^46^.

IMPα2 expression has been identified as a prognostic marker of poor outcome in many cancers^58^, including those in which the long non-coding paraspeckle RNA NEAT1 has been independently implicated, including breast^59^,^60^,^61^,^62^, colon^63^, liver^64^ and lung^65^,^66^. The link identified here between functional IMPα levels and the nuclear accumulation and localization of PSPC1 and SFPQ to paraspeckles leads us to speculate that enhanced paraspeckle formation and function may affect prognostic outcomes and provide therapeutic targets in oncology. The automated image analysis pipeline allowed for non-subjective, comprehensive examination of subcellular features on a mass scale, with the number of cells analysed extending far beyond what is feasible with manual analysis. This adaptable, high-throughput analysis pipeline could be used to answer other research questions requiring quantification of subtle changes at subcellular levels or larger imaging scales. Within the Imaris cells module, the object named “vesicles” can be used to identify spots or foci, while the “nucleus” and “cell” components will identify larger objects. These three object types do not have to be cells, nuclei or vesicles; they could be anything, micro or macro, that is identifiable by intensity thresholding. Because the parameters from these object types are linked hierarchically within Imaris, the diversity of outputs, and information about their inter-relationships, is extensive. Furthermore, custom parameters can be achieved by those with programming knowledge by creating Imaris plug-ins (XTensions) or calculating them from existing Imaris outputs within the R environment for statistical computing. As imaging techniques advance and larger 3D data sets can be acquired in shorter time frames, automated analysis pipelines such as this, which allow subtle subcellular events to be rigorously interrogated across many thousands or millions of cells, will deepen our understanding of fundamental cellular processes.

## Materials and Methods

### Constructs

GFP-tagged IMPα constructs for mammalian cell expression were generated previously^44^,^50^,^55^ and encoded full length IMPα variants (GFP-IMPα2-FL, GFP-IMPα4-FL, GFP-IMPα6-FL), ED mutants (GFP-IMPα2-ED) and truncated dominant negative IMPαs (GFP-IMPα2ΔIBB, GFP-IMPα4ΔIBB, GFP-IMPα6ΔIBB). The murine PSPC1 sequence (encoding aa 3–523) was amplified by PCR and recombined into a DsRed2-tagged mammalian cell expression vector using the Invitrogen Gateway System, as previously described^49^.

### Cell culture, transfection and indirect immunofluorescent staining

HeLa cells were maintained in Dulbecco’s modified eagle medium with 10% (v/v) fetal calf serum, Penicillin-Streptomycin (Pen-Strep), L-Glutamine and MEM Non-Essential Amino Acids in 5% CO_2_ at 37 °C. Twenty-four hrs prior to transfection, cells were seeded on round coverslips in medium lacking Pen-Strep in 12 well plates for siRNA knockdown or 24 well plates for GFP/RFP-tagged construct transfections. Lipofectamine 2000 (Invitrogen) was used to transfect PSPC1 and IMPα2 constructs, following the manufacturer’s method with 2.5 μg of DNA (single plasmid or 1.25 μg of each for co-transfection). The Dharmacon ON-TARGETplus siRNA system (GE Life Sciences) with DharmaFECT 1 transfection reagent was used as per manufacturer’s instructions. Pre-designed siRNAs targeting IMPα2 (SMARTpool L-004702-00) and IMPα4 (SMARTpool L-017477-00) were used, with a non-targeting (SCRAM siRNA) control pool (D-001810-10) as the siRNA negative control.

At 48 hrs post transfection, cells were fixed in 3.2% paraformaldehyde (in PBS) for 10 min and washed (2 × 5 min, PBS) before proceeding to indirect immunofluorescence staining, as previously^49^. To detect endogenous mouse PSPC1 and SFPQ, mouse monoclonal antibodies specific to SFPQ and to the longer PSPC1 isoform were used^67^. Rabbit anti-IMPα2 (Abcam, cat#ab84440) and goat anti-IMPα4 (Abcam, cat#ab6039) were used to detect IMPα2 and IMPα4, respectively, for immunofluorescence. Primary antibodies (1:100 in 0.5% BSA/PBS) were applied overnight at 4 °C. Secondary antibodies, rabbit anti-mouse Alexa Fluor 546 (Molecular Probes-Invitrogen, cat#A11060) for GFP/RFP-tagged transfections and donkey anti-mouse Alexa Fluor 488 (Molecular Probes-Invitrogen, cat#A21202) plus goat anti-rabbit Alexa Fluor 546 (Molecular Probes-Invitrogen, cat#A11010) or rabbit anti-goat Alexa Fluor 546 (Molecular Probes-Invitrogen, cat#A21085) for siRNA knockdown samples (1:200 in 0.5% BSA/PBS), were applied for 90 mins at room temperature.

### HeLa cell image acquisition

Imaging was performed using a Leica SP5 laser scanning confocal system (DMI6000 microscope, motorised stage, 63 × water/glycerol objective, Monash Micro Imaging Facility). Images were collected as Z-series and tiled in a 7 × 7 field of view grid (coverage of approximately 1.7 mm^2^), with resonant scanning mode (8000 Hz) used for siRNA samples (coverage of approximately 0.9 mm^2^).

### Imaris-assisted image analysis to detect cells, nucleus and paraspeckles

To assess paraspeckle number, size and PSPC1 intensity within each cell, the Imaris software package “Cells” module (Bitplane, Version 8) was used to batch process identification of cells, their nuclei, and paraspeckles, within the larger image sets described above (as shown in Fig. 1Bi–viii). Throughout the GFP-tagged IMPα transient transfection experiments, Draq5 signal identified the nucleus, GFP signal was used to identify the cell body, and nuclear foci were identified using the particular paraspeckle marker signal under investigation (i.e. PSPC1, SFPQ or DsRed2-PSPC1). For siRNA samples, DAPI signal identified the nucleus, IMPα (IMPα2 or IMPα4) signal identified the cell body, and nuclear foci were identified using the paraspeckle marker signal (PSPC1 or SFPQ). The results (output in CSV file formats) were combined and manipulated using Python scripts (Python Software Foundation, version 2.7), then analysed using the R Project for Statistical Computing scripts (The R Foundation, version 3.2). Incomplete cells with no nucleus or their nucleus on the very edge of an image (X, Y or Z image planes) were excluded from datasets for analysis and GFP thresholding was applied to datasets as described for the GFP-tagged IMPα transient transfection experiments. Additional R packages used for analysis were “car”^68^, “epitools”^69^., “geepack”^70^, “ggplot2”^71^. Graphs presented in Fig. 2 were generated using Prism (GraphPad Software, Version 6), while all others were generated using R and the “ggplot2” package.

### Statistical Analysis

For statistical testing, individual cells were assumed to be independent, but paraspeckles within each cell were assumed to be correlated. When analysing the individual cell or paraspeckle data, three outcome types were generated: 1) binary responses based on whether or not a cell was positive for paraspeckles, 2) counts data based on the number of paraspeckles within each cell (including/excluding zeroes) and 3) continuous data based on paraspeckle volume sum and paraspeckle PSPC1 intensity sum.

Comparisons between groups were made using generalised linear models (GLM); logistic regression for the binary data, linear regression for the count and continuous data. As the count and continuous data were both skewed, data were transformed using the natural logarithm to allow valid statistical inference from the linear regression models. The p-values are based on the transformed data; however, the results were then back-transformed to give estimates in the original scale for ease of interpretation. By taking the exponent of the mean of log-transformed data, the geometric mean and confidence intervals (CIs) were obtained on the original linear scale. By taking the exponent of the linear regression coefficients obtained on the log-transformed scale, the ratio of the geometric means and their 95% CIs were obtained on the original scale. Odds ratios are given for logistic regression results. When assessing data on a per paraspeckle basis, continuous outcomes were examined, which again required log transformations. Generalised estimating equations (GEE) were used to enable correlation between paraspeckles originating from the same cell^72^.

## Additional Information

**How to cite this article:** Major, A. T. *et al*. Development of a pipeline for automated, high-throughput analysis of paraspeckle proteins reveals specific roles for importin α proteins. *Sci. Rep.* **7**, 43323; doi: 10.1038/srep43323 (2017).

**Publisher's note:** Springer Nature remains neutral with regard to jurisdictional claims in published maps and institutional affiliations.

## Supplementary Material

Supplementary Figures and Tables

Supplementary Dataset SD1-A

Supplementary Dataset SD1-B

Supplementary Dataset SD1-C PART 1

Supplementary Dataset SD1-c PART 2

Supplementary Dataset SD1-D

Supplementary Dataset SD1-E

Supplementary Dataset SD1-F

Supplementary Dataset SD1-G

Supplementary Dataset SD1-H

Supplementary Dataset SD1-I

Supplementary Dataset SD1-J

Supplementary Dataset SD1-K

Supplementary Dataset SD1-L

## Figures and Tables

**Table 1:** Outcomes of modulating IMPα expression and transport function on endogenous PSPC1-positive nuclear foci.

		GFP	GFP-IMPα2-FL	GFP-IMPα2ΔIBB	GFP-IMPα2-ED	GFP-IMPα6-FL	GFP-IMPα6ΔIBB
**A**)	**Number of Cells Analysed-Total: 2602**	**813**	**166**	**519**	**459**	**431**	**214**
**B**)	**Number of PSPC1 Nuclear foci-Total: 10865**	**5079**	**595**	**728**	**1461**	**2597**	**405**
**C**)	**Number of Cells +VE for Nuclear foci**	**598**	**91**	**144**	**211**	**285**	**66**
**D**)	**% Cells +VE for foci**	**73.6%**	**54.8%**	**27.7%**	**46%**	**66.1%**	**30.8%**
	95% CI	(62.1 ↔ 85.0)%	(38.1 ↔ 71.6)%	(22.4 ↔ 33.1)%	(37.5 ↔ 54.4)%	(52.9 ↔ 79.3)%	(21.9 ↔ 39.8)%
	Odds Ratio (IMPα Normalised)		1.000	0.316	0.701	1.000	0.228
	95% CI		(Control)	(0.221 ↔ 0.454)	(0.491 ↔ 1.002)	(Control)	(0.161 ↔ 0.325)
	Significance Value (Lg Reg)			p = 0.0000*	p = 0.0505		p = 0.0000*
**Mean per cell values (All cells)**		**GFP**	**GFP-IMPα2-FL**	**GFP-IMPα2ΔIBB**	**GFP-IMPα2-ED**	**GFP-IMPα6-FL**	**GFP-IMPα6ΔIBB**
**E**)	**GM F**_**n/c**_ **per cell-PSPC1-A546**	**3.09**	**2.45**	**1.83**	**1.92**	**2.85**	**1.95**
	95% CI	(3.01 ↔ 3.17)	(2.28 ↔ 2.62)	(1.76 ↔ 1.90)	(1.85 ↔ 1.99)	(2.74 ↔ 2.97)	(1.82 ↔ 2.09)
	Ratio of GM (IMPα Normalised)		1.000	0.746	0.785	1.000	0.682
	95% CI		(Control)	(0.693 ↔ 0.804)	(0.728 ↔ 0.846)	(Control)	(0.636 ↔ 0.731)
	Significance Value (Ln Reg)			p = 0.0000*	p = 0.0000*		p = 0.0000*
**F**)	**GM intensity per cell - PSPC1-A546**	**8.77**	**7.66**	**7.16**	**9.09**	**8.79**	**6.82**
	95% CI	(8.58 ↔ 8.96)	(7.38 ↔ 7.95)	(7.00 ↔ 7.33)	(8.81 ↔ 9.38)	(8.53 ↔ 9.05)	(6.6 ↔ 7.05)
	Ratio of GM (IMPα Normalised)		1.000	0.935	1.186	1.000	0.776
	95% CI		(Control)	(0.887 ↔ 0.985)	(1.124 ↔ 1.251)	(Control)	(0.738 ↔ 0.815)
	Significance Value (Ln Reg)			p = 0.0117	p = 0.0000*		p = 0.0000*
**G**)	**GM intensity per cell - GFP**	**18.35**	**11.40**	**17.25**	**11.12**	**17.18**	**12.73**
	95% CI	(17.35 ↔ 19.41)	(10.28 ↔ 12.64)	(16.18 ↔ 18.39)	(10.5 ↔ 11.78)	(15.84 ↔ 18.65)	(11.75 ↔ 13.8)
	Ratio of GM (IMPα Normalised)		1.000	1.513	0.976	1.000	0.741
	95% CI		(Control)	(1.326 ↔ 1.726)	(0.853 ↔ 1.116)	(Control)	(0.655 ↔ 0.839)
	Significance Value (Ln Reg)			p = 0.0000*	p = 0.719		p = 0.0000*
**Mean per cell values (PSPC1-A56 nuclear foci positive cells only)**		**GFP**	**GFP-IMPα2-FL**	**GFP-IMPα2ΔIBB**	**GFP-IMPα2-ED**	**GFP-IMPα6-FL**	**GFP-IMPα6ΔIBB**
**H**)	**GM number of foci** (**per cell**)	**6.20**	**4.84**	**3.52**	**4.68**	**6.00**	**4.41**
	95% CI	(5.79 ↔ 6.63)	(4.08 ↔ 5.75)	(3.05 ↔ 4.07)	(4.16 ↔ 5.28)	(5.36 ↔ 6.72)	(3.59 ↔ 5.42)
	Ratio of GM (IMPα Normalised)		1.000	0.727	0.967	1.000	0.735
	95% CI		(Control)	(0.576 ↔ 0.916)	(0.778 ↔ 1.201)	(Control)	(0.580 ↔ 0.931)
	Significance Value (Ln Reg)			p = 0.0071*	p = 0.7621		p = 0.0107
**I**)	**GM ∑ foci volume** (**per cell**)	**1.32**	**1.08**	**0.52**	**0.84**	**1.34**	**0.75**
	95% CI	(1.18 ↔ 1.47)	(0.83 ↔ 1.41)	(0.41 ↔ 0.67)	(0.69 ↔ 1.03)	(1.12 ↔ 1.61)	(0.54 ↔ 1.06)
	Ratio of GM (IMPα Normalised)		1.000	0.482	0.779	1.000	0.562
	95% CI		(Control)	(0.331 ↔ 0.702)	(0.548 ↔ 1.107)	(Control)	(0.383 ↔ 0.824)
	Significance Value (Ln Reg)			p = 0.0001*	p = 0.1640		p = 0.0033*
**J**)	**GM ∑ foci PSPC1-A546 intensity** (**per cell**)	**5070**	**4190**	**1848**	**3183**	**5173**	**2812**
	95% CI	(4521 ↔ 5686)	(3136 ↔ 5597)	(1416 ↔ 2412)	(2558 ↔ 3960)	(4244 ↔ 6305)	(1926 ↔ 4104)
	Ratio of GM (IMPα Normalised)		1.000	0.441	0.760	1.000	0.544
	95% CI		(Control)	(0.294 ↔ 0.662)	(0.520 ↔ 1.11)	(Control)	(0.359 ↔ 0.822)
	Significance Value (Ln Reg)			p = 0.0001*	p = 0.1560		p = 0.0039*
**Mean values per PSPC1-A546 nuclear foci**		**GFP**	**GFP-IMPα2-FL**	**GFP-IMPα2ΔIBB**	**GFP-IMPα2-ED**	**GFP-IMPα6-FL**	**GFP-IMPα6ΔIBB**
**K**)	**GM foci volume** (**per foci**)	**0.190**	**0.209**	**0.156**	**0.212**	**0.215**	**0.173**
	95% CI	(0.185 ↔ 0.196)	(0.193 ↔ 0.227)	(0.145 ↔ 0.168)	(0.201 ↔ 0.224)	(0.207 ↔ 0.224)	(0.157 ↔ 0.191)
	Ratio of GM (IMPα Normalised)		1.000	0.744	1.013	1.000	0.804
	95% CI		(Control)	(0.613 ↔ 0.903)	(0.785 ↔ 1.307)	(Control)	(0.652 ↔ 0.991)
	Significance Value (GEE)			p = 0.0028*	p = 0.9225		p = 0.041
**L**)	**GM foci PSPC1-A546 voxel intensity** (**per foci**)	**90.70**	**91.12**	**89.32**	**94.90**	**92.33**	**90.31**
	95% CI	(90.35 ↔ 91.06)	(90.07 ↔ 92.12)	(88.44 ↔ 90.20)	(93.90 ↔ 95.91)	(91.71 ↔ 92.95)	(89.00 ↔ 91.64)
	Ratio of GM (IMPα Normalised)		1.000	0.980	1.041	1.000	0.989
	95% CI		(Control)	(0.954 ↔ 1.01)	(0.976 ↔ 1.11)	(Control)	(0.958 ↔ 1.022)
	Significance Value (GEE)			p = 0.15	p = 0.22		p = 0.5086
**M**)	**GM ∑ foci PSPC1-A546 intensity** (**per foci**)	**701**	**792**	**549**	**822**	**815**	**639**
	95% CI	(678 ↔ 723)	(722 ↔ 869)	(505 ↔ 597)	(770 ↔ 877)	(778 ↔ 854)	(571 ↔ 716)
	Ratio of GM (IMPα Normalised)		1.000	0.693	1.037	1.000	0.797
	95% CI		(Control)	(0.554 ↔ 0.867)	(0.752 ↔ 1.431)	(Control)	(0.628 ↔ 1.010)
	Significance Value (GEE)			p = 0.0013*	p = 0.8225		p = 0.061

The analysed cell numbers for each GFP-tagged IMPα transfection group, the number of detected PSPC1-positive nuclear foci and proportion of cells determined to contain PSPC1 nuclear foci (detected by indirect PSPC1 immunofluorescence with an Alexa Fluor 546 [A546] secondary antibody) are presented. Samples were assessed on a per cell or per PSPC1 nuclear foci basis, with geometric means (GM) and 95% confidence intervals (95% CI) calculated. To determine significant differences between groups, a logistic regression (Lg Reg) model was used for PSPC1 foci positive/negative cells, linear regression (Ln Reg) models were used for per cell data and generalised estimating equations (GEE) were used for per PSPC1 nuclear foci data. Comparative significance values using IMPα-FL as the reference groups (set at 1.000) are shown. Using Bonferroni correction, the significance threshold was reassigned from ≤0.05 to ≤0.008 (0.05 ÷ 6 experimental groups), with those outcomes below the threshold indicated (*). Further details are provided in Fig. 2A with additional samples and analysis parameters included in Supplementary Tables S2 and S5.

**Table 2:** Outcomes of modulating IMPα expression and transport function on endogenous SFPQ-positive nuclear foci.

		GFP	GFP-IMPα2-FL	GFP-IMPα2ΔIBB	GFP-IMPα2-ED	GFP-IMPα4-FL	GFP-IMPα4ΔIBB	GFP-IMPα6-FL	GFP-IMPα6ΔIBB
**A**)	**Number of cells analysed - Total: 2258**	**566**	**192**	**301**	**143**	**173**	**299**	**273**	**311**
**B**)	**SFPQ nuclear foci - Total: 14750**	**4570**	**1529**	**1666**	**845**	**1787**	**1297**	**2123**	**933**
**C**)	**Number of cells + VE for nuclear foci**	**460**	**161**	**174**	**84**	**158**	**180**	**234**	**128**
**D**)	**% Cells +VE for foci**	**81.3%**	**83.9%**	**57.8%**	**58.7%**	**91.3%**	**60.2%**	**85.7%**	**41.2%**
	95% CI	(64.1 ↔ 98.4)%	(51.6 ↔ 116.1)%	(44.6 ↔ 71.0)%	(39.2 ↔ 78.3)%	(43.0 ↔ 139.7)%	(46.3 ↔ 74.1)%	(56.7 ↔ 114.8)%	(31.9 ↔ 50.5)%
	Odds Ratio (IMPα Normalised)		1.000	0.264	0.274	1.000	0.144	1.000	0.117
	95% CI		(Control)	(0.169 ↔ 0.413)	(0.165 ↔ 0.456)	(Control)	(0.081 ↔ 0.256)	(Control)	(0.078 ↔ 0.175)
	Significance Value (Lg Reg)			p = 0.0000*	p = 0.0000*		p = 0.0000*		p = 0.0000*
**Mean per cell values (All cells)**		**GFP**	**GFP-IMPα2-FL**	**GFP-IMPα2ΔIBB**	**GFP-IMPα2-ED**	**GFP-IMPα4-FL**	**GFP-IMPα4ΔIBB**	**GFP-IMPα6-FL**	**GFP-IMPα6ΔIBB**
**E**)	**GM F**_**n/c**_ **per cell - SFPQ-A546**	**6.24**	**4.75**	**3.21**	**2.80**	**5.49**	**3.93**	**5.32**	**2.66**
	95% CI	(6.01 ↔ 6.47)	(4.43 ↔ 5.09)	(2.93 ↔ 3.50)	(2.57 ↔ 3.06)	(5.21 ↔ 5.79)	(3.70 ↔ 4.19)	(5.05 ↔ 5.62)	(2.44 ↔ 2.90)
	Odds Ratio (IMPα Normalised)		1.000	0.675	0.590	1.000	0.716	1.000	0.500
	95% CI		(Control)	(0.609 ↔ 0.749)	(0.521 ↔ 0.668)	(Control)	(0.643 ↔ 0.797)	(Control)	(0.456 ↔ 0.549)
	Significance Value (Ln Reg)			p = 0.0000*	p = 0.0000*		p = 0.0000*		p = 0.0000*
**F**)	**GM intensity per cell - SFPQ-A546**	**7.13**	**9.22**	**6.70**	**11.04**	**9.68**	**7.59**	**8.55**	**5.34**
	95% CI	(6.95 ↔ 7.33)	(8.70 ↔ 9.77)	(6.29 ↔ 7.14)	(9.90 ↔ 12.32)	(9.22 ↔ 10.16)	(7.24 ↔ 9.95)	(8.21 ↔ 8.90)	(5.07 ↔ 5.63)
	Odds Ratio (IMPα Normalised)		1.000	0.727	1.198	1.000	0.784	1.000	0.625
	95% CI		(Control)	(0.672 ↔ 0.786)	(1.091 ↔ 1.315)	(Control)	(0.723 ↔ 0.849)	(Control)	(0.583 ↔ 0.670)
	Significance Value (Ln Reg)			p = 0.0000*	p = 0.0002*		p = 0.0000*		p = 0.0000*
**G**)	**GM intensity per cell – GFP**	**13.38**	**9.34**	**11.91**	**8.40**	**9.42**	**12.50**	**12.91**	**11.00**
	95% CI	(12.76 ↔ 14.04)	(8.63 ↔ 10.11)	(11.20 ↔ 12.67)	(7.76 ↔ 9.10)	(8.67 ↔ 10.24)	(11.62 ↔ 13.38)	(11.66 ↔ 14.28)	(10.17 ↔ 11.90)
	Odds Ratio (IMPα Normalised)		1.000	1.276	0.900	1.000	1.326	1.000	0.852
	95% CI		(Control)	(1.138 ↔ 1.429)	(0.785 ↔ 1.031)	(Control)	(1.179 ↔ 1.492)	(Control)	(0.769 ↔ 0.944)
	Significance Value (Ln Reg)			p = 0.0000*	p = 0.1286		p = 0.0000*		p = 0.0022*
**Mean per cell values (SFPQ-A546 nuclear foci positive cells only)**		**GFP**	**GFP-IMPα2-FL**	**GFP-IMPα2ΔIBB**	**GFP-IMPα2-ED**	**GFP-IMPα4-FL**	**GFP-IMPα4ΔIBB**	**GFP-IMPα6-FL**	**GFP-IMPα6ΔIBB**
**H**)	**GM number of foci** (**per cell**)	**7.61**	**6.25**	**7.10**	**7.50**	**8.13**	**5.14**	**6.85**	**5.25**
	95% CI	(7.07 ↔ 8.20)	(5.39 ↔ 7.26)	(6.24 ↔ 8.07)	(6.23 ↔ 9.01)	(7.08 ↔ 9.34)	(4.54 ↔ 5.82)	(6.17 ↔ 7.60)	(4.51 ↔ 6.11)
	Odds Ratio (IMPα Normalised)		1.000	1.135	1.199	1.000	0.632	1.000	0.766
	95% CI		(Control)	(0.945 ↔ 1.363)	(0.957 ↔ 1.501)	(Control)	(0.527 ↔ 0.759)	(Control)	(0.637 ↔ 0.921)
	Significance Value (Ln Reg)			p = 0.1751	p = 0.1152		p = 0.0000*		p = 0.0046*
**I**)	**GM ∑ foci volume** (**per cell**)	**2.68**	**1.96**	**2.44**	**2.33**	**3.11**	**1.16**	**1.99**	**1.38**
	95% CI	(2.33 ↔ 3.07)	(1.51 ↔ 2.54)	(1.94 ↔ 3.06)	(1.70 ↔ 3.20)	(2.48 ↔ 3.89)	(0.95 ↔ 1.41)	(1.65 ↔ 2.41)	(1.05 ↔ 1.80)
	Odds Ratio (IMPα Normalised)		1.000	1.247	1.1931	1.000	0.3731	1.000	0.691
	95% CI		(Control)	(0.904 ↔ 1.722)	(0.802 ↔ 1.774)	(Control)	(0.271 ↔ 0.515)	(Control)	(0.499 ↔ 0.955)
	Significance Value (Ln Reg)			p = 0.1792	p = 0.3835		p = 0.0000*		p = 0.0254
**J**)	**GM ∑ foci SFPQ-A546 intensity** (**per cell**)	**9422**	**7276**	**9306**	**9159**	**12102**	**4343**	**7557**	**5052**
	95% CI	(8159 ↔ 10879)	(5567 ↔ 9509)	(7313 ↔ 11843)	(6604 ↔ 12703)	(9600 ↔ 15255)	(3543 ↔ 5322)	(6200 ↔ 9212)	(3803 ↔ 6712)
	Odds Ratio (IMPα Normalised)		1.000	1.279	1.259	1.000	0.359	1.000	0.669
	95% CI		(Control)	(0.914 ↔ 1.790)	(0.833 ↔ 1.903)	(Control)	(0.257 ↔ 0.502)	(Control)	(0.477 ↔ 0.973)
	Significance Value (Ln Reg)			p = 0.1511	p = 0.2753		p = 0.0000*		p = 0.0195
**Mean values per SFPQ-A546 nuclear foci**		**GFP**	**GFP-IMPα2-FL**	**GFP-IMPα2ΔIBB**	**GFP-IMPα2-ED**	**GFP-IMPα4-FL**	**GFP-IMPα4ΔIBB**	**GFP-IMPα6-FL**	**GFP-IMPα6ΔIBB**
**K**)	**GM foci volume** (**per foci**)	**0.318**	**0.329**	**0.317**	**0.286**	**0.320**	**0.202**	**0.264**	**0.264**
	95% CI	(0.307 ↔ 0.329)	(0.309 ↔ 0.349)	(0.300 ↔ 0.308)	(0.266 ↔ 0.308)	(0.303 ↔ 0.337)	(0.191 ↔ 0.214)	(0.252 ↔ 0.277)	(0.246 ↔ 0.283)
	Odds Ratio (IMPα Normalised)		1.000	0.965	0.871	1.000	0.632	1.000	0.998
	95% CI		(Control)	(0.773 ↔ 1.204)	(0.693 ↔ 1.094)	(Control)	(0.538 ↔ 0.741)	(Control)	(0.842 ↔ 1.184)
	Significance Value (GEE)			p = 0.750	p = 0.234		p = 0.0000*		p = 0.9837
**L**)	**GM foci SFPQ-A546 voxel intensity** (**per foci**)	**89.56**	**87.21**	**89.01**	**89.26**	**90.16**	**87.65**	**87.48**	**88.31**
	95% CI	(89.17 ↔ 89.95)	(86.57 ↔ 87.85)	(88.32 ↔ 89.71)	(88.42 ↔ 90.11)	(89.51 ↔ 90.82)	(87.08 ↔ 88.22)	(86.97 ↔ 87.99)	(87.60 ↔ 89.03)
	Odds Ratio (IMPα Normalised)		1.000	1.02	1.02	1.000	0.972	1.000	1.009
	95% CI		(Control)	(0.998 ↔ 1.05)	(0.999 ↔ 1.05)	(Control)	(0.953 ↔ 0.991)	(Control)	(0.991 ↔ 1.03)
	Significance Value (GEE)			p = 0.0736	p = 0.0582		p = 0.0045*		p = 0.3070
**M**)	**GM ∑ foci SFPQ-A546 intensity** (**per foci**)	**1107**	**1202**	**1188**	**1071**	**1204**	**728**	**964**	**963**
	95% CI	(1068 ↔ 1149)	(1126 ↔ 1282)	(1117 ↔ 1263)	(988 ↔ 1160)	(1135 ↔ 1278)	(684 ↔ 775)	(914 ↔ 1017)	(891 ↔ 1041)
	Odds Ratio (IMPα Normalised)		1.000	0.993	0.891	1.000	0.604	1.000	0.999
	95% CI		(Control)	(0.782 ↔ 1.260)	(0.701 ↔ 1.132)	(Control)	(0.507 ↔ 0.720)	(Control)	(0.835 ↔ 1.196)
	Significance Value (GEE)			p = 0.952	p = 0.344		p = 0.0000*		p = 0.9931

The analysed cell numbers for each GFP-tagged IMPα transfection group, the number of detected SFPQ-positive nuclear foci and proportion of cells determined to contain SFPQ nuclear foci (detected by indirect SFPQ immunofluorescence with an Alexa Fluor 546 [A546] secondary antibody) are presented. Samples were assessed on a per cell or per SFPQ nuclear foci basis, with geometric means (GM) and 95% confidence intervals (95% CI) calculated. To determine significant differences between groups, a logistic regression (Lg Reg) model was used for SFPQ foci positive/negative cells, linear regression (Ln Reg) models were used for per cell data and generalised estimating equations (GEE) were used for per PSPC1 nuclear foci data. Comparative significance values using IMPα-FL as the reference groups (set at 1.000) are shown. Using Bonferroni correction, the significance threshold was reassigned from ≤0.05 to ≤0.0063 (0.05 ÷ 8 experimental groups), with those outcomes below the threshold indicated (*). Further details are provided in Fig. 2C with additional samples and analysis parameters included in Supplementary Tables S3 and S6.

**Table 3:** Outcomes of modulating IMPα expression and transport function on exogenous dsRed2-PSPC1-positive nuclear foci.

		GFP	GFP-IMPα2-FL	GFP-IMPα2ΔIBB	GFP-IMPα2-ED	GFP-IMPα4-FL	GFP-IMPα4ΔIBB	GFP-IMPα6-FL	GFP-IMPα6ΔIBB
**A**)	**Number of cells analysed - Total: 2530**	**180**	**228**	**241**	**446**	**317**	**386**	**513**	**219**
**B**)	**DsRed2-PSPC1 nuclear foci - Total: 38657**	**2508**	**3392**	**2147**	**6218**	**10853**	**4339**	**5473**	**3745**
**C**)	**Number of cells +VE for nuclear foci**	**50**	**117**	**92**	**195**	**189**	**143**	**195**	**87**
**D**)	**% Cells +VE for foci**	**27.8%**	**51.3%**	**38.2%**	**43.7%**	**59.6%**	**37%**	**38%**	**39.7%**
	95% CI	(18.7 ↔ 36.8)%	(38.0 ↔ 64.6)%	(28.3 ↔ 48.1)%	(35.5 ↔ 51.9)%	(46.2 ↔ 73.0)%	(29.4 ↔ 44.7)%	(31.2 ↔ 44.8)%	(29.0 ↔ 50.5)%
	Odds Ratio (IMPα Normalised)		1.000	0.586	0.737	1.000	0.399	1.000	1.075
	95% CI		(Control)	(0.406 ↔ 0.846)	(0.535 ↔ 1.015)	(Control)	(0.294 ↔ 0.541)	(Control)	(0.777 ↔ 1.486)
	Significance Value (Lg Reg)			p = 0.0042*	p = 0.0614		p = 0.0000*		p = 0.6625
**Mean per cell values (All cells)**		**GFP**	**GFP-IMPα2-FL**	**GFP-IMPα2ΔIBB**	**GFP-IMPα2-ED**	**GFP-IMPα4-FL**	**GFP-IMPα4ΔIBB**	**GFP-IMPα6-FL**	**GFP-IMPα6ΔIBB**
**E**)	**GM F**_**n/c**_ **per cell - DsRed2-PSPC1**	**1.66**	**1.86**	**1.62**	**1.60**	**1.94**	**1.78**	**1.56**	**1.86**
	95% CI	(1.57 ↔ 1.76)	(1.75 ↔ 1.98)	(1.53 ↔ 1.72)	(1.54 ↔ 1.66)	(1.82 ↔ 2.06)	(1.70 ↔ 1.87)	(1.51 ↔ 1.62)	(1.72 ↔ 3.62)
	Odds Ratio (IMPα Normalised)		1.000	0.873	0.860	1.000	0.922	1.000	1.191
	95% CI		(Control)	(0.801 ↔ 0.951)	(0.798 ↔ 0.928)	(Control)	(0.859 ↔ 0.989)	(Control)	(1.105 ↔ 1.284)
	Significance Value (Ln Reg)			p = 0.0019*	p = 0.0001*		p = 0.0235		p = 0.0000*
**F**)	**GM intensity per cell - DsRed2-PSPC1**	**5.78**	**5.85**	**4.28**	**5.19**	**6.38**	**4.95**	**4.40**	**4.59**
	95% CI	(5.57 ↔ 6.00)	(5.66 ↔ 6.06)	(4.123 ↔ 4.434)	(4.99 ↔ 5.40)	(6.12 ↔ 6.65)	(4.76 ↔ 5.15)	(4.28 ↔ 4.53)	(4.38 ↔ 4.81)
	Odds Ratio (IMPα Normalised)		1.000	0.730	0.887	1.000	0.777	1.000	1.042
	95% CI		(Control)	(0.685 ↔ 0.779)	(0.838 ↔ 0.939)	(Control)	(0.737 ↔ 0.819)	(Control)	(0.986 ↔ 1.103)
	Significance Value (Ln Reg)			p = 0.0000*	p = 0.0000*		p = 0.0000*		p = 0.144
**G**)	**GM intensity per cell – GFP**	**24.51**	**11.10**	**18.05**	**13.04**	**10.59**	**14.31**	**15.74**	**14.46**
	95% CI	(21.6 ↔ 27.9)	(10.30 ↔ 11.96)	(16.39 ↔ 19.89)	(12.29 ↔ 13.84)	(9.99 ↔ 11.22)	(13.25 ↔ 15.47)	(14.67 ↔ 16.88)	(13.05 ↔ 16.01)
	Odds Ratio (IMPα Normalised)		1.000	1.627	1.175	1.000	1.352	1.000	0.919
	95% CI		(Control)	(1.427 ↔ 1.855)	(1.047 ↔ 1.139)	(Control)	(1.214 ↔ 1.505)	(Control)	(0.819 ↔ 1.030)
	Significance Value (Ln Reg)			p = 0.0000*	p = 0.0062*		p = 0.0000*		p = 0.1462
**Mean per cell values (DsRed2-PSPC1 nuclear foci positive cells only)**		**GFP**	**GFP-IMPα2-FL**	**GFP-IMPα2ΔIBB**	**GFP-IMPα2-ED**	**GFP-IMPα4-FL**	**GFP-IMPα4ΔIBB**	**GFP-IMPα6-FL**	**GFP-IMPα6ΔIBB**
**H**)	**GM number of foci** (**per cell**)	**12.70**	**11.51**	**7.49**	**10.32**	**14.94**	**7.78**	**9.04**	**15.58**
	95% CI	(7.88 ↔ 20.48)	(9.04 ↔ 14.65)	(5.53 ↔ 10.13)	(8.39 ↔ 12.70)	(11.70 ↔ 19.07)	(5.96 ↔ 10.15)	(7.33 ↔ 11.14)	(11.30 ↔ 21.50)
	Odds Ratio (IMPα Normalised)		1.000	0.651	0.897	1.000	0.521	1.000	1.725
	95% CI		(Control)	(0.427 ↔ 0.992)	(0.629 ↔ 1.278)	(Control)	(0.372 ↔ 0.729)	(Control)	(1.167 ↔ 2.549)
	Significance Value (Ln Reg)			p = 0.0462	p = 0.5481		p = 0.0001*		p = 0.0063*
**I**)	**GM ∑ foci volume** (**per cell**)	**2.15**	**1.88**	**1.03**	**1.43**	**2.48**	**1.00**	**1.29**	**2.59**
	95% CI	(1.13 ↔ 4.08)	(1.37 ↔ 2.53)	(0.71 ↔ 1.51)	(1.10 ↔ 1.86)	(1.79 ↔ 3.42)	(0.71 ↔ 1.42)	(0.97 ↔ 1.71)	(1.71 ↔ 3.91)
	Odds Ratio (IMPα Normalised)		1.000	0.550	0.761	1.000	0.405	1.000	2.003
	95% CI		(Control)	(0.316 ↔ 0.956)	(0.478 ↔ 1.211)	(Control)	(0.261 ↔ 0.628)	(Control)	(1.200 ↔ 3.343)
	Significance Value (Ln Reg)			p = 0.0342	p = 0.2490		p = 0.0001*		p = 0.0080
**J**)	**GM ∑ foci DsRed2-PSPC1 intensity** (**per cell**)	**8724**	**7704**	**3921**	**5499**	**10240**	**3838**	**5102**	**10400**
	95% CI	(4397 ↔ 17310)	(5476 ↔ 10840)	(2602 ↔ 5908)	(4152 ↔ 7283)	(7250 ↔ 14460)	(2649 ↔ 5562)	(3744 ↔ 6954)	(6684 ↔ 16190)
	Odds Ratio (IMPα Normalised)		1.000	0.509	0.714	1.000	0.375	1.000	2.039
	95% CI		(Control)	(0.281 ↔ 9.214)	(0.434 ↔ 1.175)	(Control)	(0.234 ↔ 6.012)	(Control)	(1.177 ↔ 3.532)
	Significance Value (Ln Reg)			p = 0.0259	p = 0.1850		p = 0.0001*		p = 0.0112
**Mean values per DsRed2-PSPC1 nuclear foci**		**GFP**	**GFP-IMPα2-FL**	**GFP-IMPα2ΔIBB**	**GFP-IMPα2-ED**	**GFP-IMPα4-FL**	**GFP-IMPα4ΔIBB**	**GFP-IMPα6-FL**	**GFP-IMPα6ΔIBB**
**K**)	**GM foci volume** (**per foci**)	**0.215**	**0.162**	**0.159**	**0.155**	**0.229**	**0.179**	**0.197**	**0.180**
	95% CI	(0.207 ↔ 0.224)	(0.157 ↔ 0.167)	(0.152 ↔ 0.166)	(0.151 ↔ 0.159)	(0.225 ↔ 0.234)	(0.174 ↔ 0.185)	(0.192 ↔ 0.202)	(0.174 ↔ 0.185)
	Odds Ratio (IMPα Normalised)		1.000	0.980	0.956	1.000	0.783	1.000	0.911
	95% CI		(Control)	(0.710 ↔ 1.353)	(0.779 ↔ 1.175)	(Control)	(0.629 ↔ 0.975)	(Control)	(0.709 ↔ 1.172)
	Significance Value (GEE)			p = 0.9026	p = 0.6707		p = 0.0290		p = 0.469
**L**)	**GM foci DsRed2-PSPC1 voxel intensity** (**per foci**)	**99.89**	**95.18**	**97.14**	**92.37**	**102.8**	**95.52**	**102.1**	**97.08**
	95% CI	(98.84 ↔ 100.9)	(94.42 ↔ 95.94)	(95.95 ↔ 98.34)	(91.83 ↔ 92.92)	(102.3 ↔ 103.4)	(94.76 ↔ 96.29)	(101.3 ↔ 102.9)	(96.22 ↔ 97.94)
	Odds Ratio (IMPα Normalised)		1.000	1.023	0.971	1.000	0.929	1.000	0.951
	95% CI		(Control)	(0.931 ↔ 1.12)	(0.924 ↔ 1.02)	(Control)	(0.861 ↔ 1.002)	(Control)	(0.880 ↔ 1.026)
	Significance Value (GEE)			p = 0.637	p = 0.232		p = 0.0565		p = 0.1947
**M**)	**GM ∑ foci DsRed2-PSPC1 intensity** (**per foci**)	**885**	**614**	**613**	**577**	**970**	**694**	**820**	**707**
	95% CI	(845 ↔ 928)	(590 ↔ 638)	(581 ↔ 647)	(560 ↔ 594)	(946 ↔ 994)	(669 ↔ 720)	(793 ↔ 848)	(679 ↔ 736)
	Odds Ratio (IMPα Normalised)		1.000	1.00	0.94	1.000	0.716	1.000	0.862
	95% CI		(Control)	(0.649 ↔ 1.55)	(0.726 ↔ 1.22)	(Control)	(0.532 ↔ 0.963)	(Control)	(0.617 ↔ 1.205)
	Significance Value (GEE)			p = 0.991	p = 0.6383		p = 0.0270		p = 0.385

The analysed cell numbers for each GFP-tagged IMPα group, all co-transfected with DsRed2-PSPC1, the number of detected DsRed2-PSPC1-positive nuclear foci and proportion of cells determined to contain DsRed2-PSPC1 nuclear foci (detected by DsRed2-PSPC1 fluorescence) are presented. Samples were assessed on a per cell or per DsRed2-PSPC1 nuclear foci basis, with geometric means (GM) and 95% confidence intervals (95% CI) calculated. To determine significant differences between groups, a logistic regression (Lg Reg) model was used for DsRed2-PSPC1 foci positive/negative cells, linear regression (Ln Reg) models were used for per cell data and generalised estimating equations (GEE) were used for per DsRed2-PSPC1 nuclear foci data. Comparative significance values using IMPα-FL as the reference groups (set at 1.000) are shown. Using Bonferroni correction, the significance threshold was reassigned from ≤0.05 to ≤0.0063 (0.05 ÷ 8 experimental groups), with those outcomes below the threshold indicated (*). Further details are provided in Fig. 2B with additional samples and analysis parameters included in Supplementary Tables S4 and S7.
