# Supplementary material for: Development of a pipeline for automated, high-throughput analysis of paraspeckle proteins reveals specific roles for importin α proteins
Source: Sci Rep. 2017 Feb 27;7:43323. doi: 10.1038/srep43323 (PMC5379994; doi:10.1038/srep43323)
Supplement: Supplementary Figures and Tables [file srep43323-s1.pdf]

Development of a pipeline for automated, high-throughput analysis of paraspeckle proteins reveals specific roles for importin α proteins

Andrew T. Major, Yoichi Miyamoto, Camden Y. Lo, David A. Jans, Kate L. Loveland

Supplementary Tables and Figures

Tables:

Supplementary Table S1

Supplementary Table S2

Supplementary Table S3

Supplementary Table S4

Supplementary Table S5

Supplementary Table S6

Supplementary Table S7

Supplementary Table S8

Supplementary Table S9

Supplementary Table S10

Supplementary Table S11

Supplementary Table S12

Supplementary Table S13

Supplementary Table S14

Supplementary Table S15

Figures:

Supplementary Figure S1

Supplementary Table S1: Comparisons of results using automated cell detection and manual cell cropping methods.

Total per sample values		GFP	GFP-IMPα2-FL	GFP-IMPα2 ΔIBB	GFP-IMPα2-ED	GFP-IMPα4-FL	GFP-IMPα4 ΔIBB	GFP-IMPα6-FL	GFP-IMPα6 ΔIBB
Number of Cells (Nuclei)	Auto PSPC1 Total: 6108	813	166	519	459		550	431	214
	Manual PSPC1 Total: 902		133	482	287				
	Auto dsRed2-PSPC1 Total: 2886	180	228	241	446	317	386	513	219
Number of Nuclear Foci	Auto PSPC1 Total: 26883	5080	595	728	1460		815	2600	405
	Manual PSPC1 Total: 2305		461	831	1013				
	Auto dsRed2-PSPC1 Total: 58320	2510	3390	2150	6220	10900	4340	5470	3740
% Cells (Nuclei) Positive for Foci	Auto PSPC1	73.6	54.8	27.7	46.0		27.5	66.1	30.8
	Manual PSPC1		51.1	32.6	46.7				
	Auto dsRed2-PSPC1	27.8	51.3	38.2	43.7	59.6	37	38	39.7
Mean per cell values (PSPC1 foci positive cells)									
GM Number of Nuclear Foci per Cell (Nuclei)	Auto PSPC1	6.2	4.84	3.52	4.68		3.7	6	4.41
	Manual PSPC1		5.05	3.61	5.13				
	Auto dsRed2-PSPC1	12.7	11.5	7.49	10.3	14.9	7.78	9.04	15.6
GM Sum Volume of Nuclear Foci per Cell (Cell)	Auto PSPC1	1.32	1.08	0.523	0.845		0.605	1.34	0.754
	Manual PSPC1		1.09	0.51	0.93				
	Auto dsRed2-PSPC1	2.15	1.88	1.03	1.43	2.48	1	1.29	2.59
GM Sum Nuclear Foci Int per Cell (Cell) - PSPC1-A546	Auto PSPC1	5070	4190	1850	3180		2170	5170	2810
	Manual PSPC1		4176	1812	3496				
	Auto dsRed2-PSPC1	8720	7700	3920	5500	10200	3840	5100	10400
Mean values per PSPC1 foci									
GM Nuclear Foci Volume	Auto PSPC1	0.19	0.209	0.156	0.212		0.165	0.215	0.173
	Manual PSPC1		0.206	0.150	0.201				
	Auto dsRed2-PSPC1	0.215	0.162	0.159	0.155	0.229	0.179	0.197	0.18
GM Nuclear Foci Int - PSPC1-A546	Auto PSPC1	90.7	91.1	89.3	94.9		87.8	92.3	90.3
	Manual PSPC1		90.6	88.8	91.8				
	Auto dsRed2-PSPC1	99.9	95.2	97.1	92.4	103	95.5	102	97.1
GM Sum Nuclear Foci Int - PSPC1-A546	Auto PSPC1	701	792	549	822		584	815	639
	Manual PSPC1		766	529	756				
	Auto dsRed2-PSPC1	885	613	613	577	970	694	820	707

Comparative values across samples using endogenous (“Auto PSPC1” – data from Table 1) and exogenous (“Manual PSPC1” – data from Table 2) PSPC1 to detect paraspeckles. This allows for direct comparison between automatically detected cells/nuclei (“Auto PSPC1”) and previously published data where cells were manually identified and cropped [“Manual PSPC1”[49]].

Supplementary Table S2: Outcomes of modulating IMPα expression and transport function on endogenous PSPC1-positive nuclear foci.

	GFP	GFP-IMPα2-FL	GFP-IMPα2ΔIBB	GFP-IMPα2-ED	GFP-IMPα6-FL	GFP-IMPα6ΔIBB
A) Number of Cells Analysed - Total: 2602	813	166	519	459	431	214
B) Number of PSPC1 Nuclear foci - Total: 10865	5079	595	728	1461	2597	405
C) Number of Cells +VE for Nuclear foci	598	91	144	211	285	66
D) % Cells +VE for foci	73.6%	54.8%	27.7%	46%	66.1%	30.8%
95% CI	(62.1↔85.0)%	(38.1↔71.6)%	(22.4↔33.1)%	(37.5↔54.4)%	(52.9↔79.3)%	(21.9↔39.8)%
Odds Ratio (GFP Normalised)	1.000	0.436	0.138	0.306	0.702	0.160
95% CI	(Control)	(0.310↔0.615)	(0.108↔0.177)	(0.240↔0.389)	(0.545↔0.904)	(0.115↔0.223)
Significance Value (Lg Reg)		p=0.0000 *	p=0.0000 *	p=0.0000 *	p=0.0060 *	p=0.0000 *
Odds Ratio (IMPα Normalised)		1.000	0.316	0.701	1.000	0.228
95% CI		(Control)	(0.221↔0.454)	(0.491↔1.002)	(Control)	(0.161↔0.325)
Significance Value (Lg Reg)			p=0.0000 *	p=0.0505		p=0.0000 *
**Mean per cell values (All cells)**	**GFP**	**GFP-IMPα2-FL**	**GFP-IMPα2ΔIBB**	**GFP-IMPα2-ED**	**GFP-IMPα6-FL**	**GFP-IMPα6ΔIBB**
E) GM $F_{n/c}$ per cell - PSPC1-A546	3.09	2.45	1.83	1.92	2.85	1.95
95% CI	(3.01↔3.17)	(2.28↔2.62)	(1.76↔1.90)	(1.85↔1.99)	(2.74↔2.97)	(1.82↔2.09)
Ratio of GM (GFP Normalised)	1.000	0.792	0.591	0.622	0.924	0.630
95% CI	(Control)	(0.738↔0.850)	(0.564↔0.619)	(0.592↔0.653)	(0.879↔0.971)	(0.591↔0.671)
Significance Value (Ln Reg)		p=0.0018 *	p=0.0000 *	p=0.0000 *	p=0.0018 *	p=0.0000 *
Ratio of GM (IMPα Normalised)		1.000	0.746	0.785	1.000	0.682
95% CI		(Control)	(0.693↔0.804)	(0.728↔0.846)	(Control)	(0.636↔0.731)
Significance Value (Ln Reg)			p=0.0000 *	p=0.0000 *		p=0.0000 *
F) GM intensity per cell - PSPC1-A546	8.77	7.66	7.16	9.09	8.79	6.82
95% CI	(8.58↔8.96)	(7.38↔7.95)	(7.00↔7.33)	(8.81↔9.38)	(8.53↔9.05)	(6.6↔7.05)
Ratio of GM (GFP Normalised)	1.000	0.874	0.817	1.036	1.002	0.778
95% CI	(Control)	(0.831↔0.919)	(0.790↔0.844)	(1.001↔1.073)	(0.968↔1.038)	(0.743↔0.814)
Significance Value (Ln Reg)		p=0.0000 *	p=0.0000 *	p=0.0417	p=0.8979	p=0.0000 *
Ratio of GM (IMPα Normalised)		1.000	0.935	1.186	1.000	0.776
95% CI		(Control)	(0.887↔0.985)	(1.124↔1.251)	(Control)	(0.738↔0.815)
Significance Value (Ln Reg)			p=0.0117	p=0.0000 *		p=0.0000 *
G) GM intensity per cell - GFP	18.35	11.40	17.25	11.12	17.18	12.73
95% CI	(17.35↔19.41)	(10.28↔12.64)	(16.18↔18.39)	(10.5↔11.78)	(15.84↔18.65)	(11.75↔13.8)
Ratio of GM (GFP Normalised)	1.000	0.621	0.940	0.606	0.936	0.694
95% CI	(Control)	(0.548↔0.705)	(0.865↔1.021)	(0.556↔0.661)	(0.857↔1.023)	(0.619↔0.777)
Significance Value (Ln Reg)		p=0.0000 *	p=0.1440	p=0.0000 *	p=0.1440	p=0.0000 *
Ratio of GM (IMPα Normalised)		1.000	1.513	0.976	1.000	0.741
95% CI		(Control)	(1.326↔1.726)	(0.853↔1.116)	(Control)	(0.655↔0.839)
Significance Value (Ln Reg)			p=0.0000 *	p=0.719		p=0.0000 *
**Mean per cell values (PSPC1-A56 nuclear foci positive cells only)**	**GFP**	**GFP-IMPα2-FL**	**GFP-IMPα2ΔIBB**	**GFP-IMPα2-ED**	**GFP-IMPα6-FL**	**GFP-IMPα6ΔIBB**
H) GM number of foci (per cell)	6.20	4.84	3.52	4.68	6.00	4.41
95% CI	(5.79↔6.63)	(4.08↔5.75)	(3.05↔4.07)	(4.16↔5.28)	(5.36↔6.72)	(3.59↔5.42)
Ratio of GM (GFP Normalised)	1.000	0.782	0.568	0.756	0.968	0.711
95% CI	(Control)	(0.643↔0.950)	(0.484↔0.667)	(0.658↔0.868)	(0.855↔1.097)	(0.569↔0.891)
Significance Value (Ln Reg)		p=0.0133	p=0.0000 *	p=0.0001 *	p=0.6150	p=0.0030 *
Ratio of GM (IMPα Normalised)		1.000	0.727	0.967	1.000	0.735
95% CI		(Control)	(0.576↔0.916)	(0.778↔1.201)	(Control)	(0.580↔0.931)
Significance Value (Ln Reg)			p=0.0071 *	p=0.7621		p=0.0107

I) GM ∑ foci volume (per cell)	1.32	1.08	0.52	0.84	1.34	0.75
95% CI	(1.18↔1.47)	(0.83↔1.41)	(0.41↔0.67)	(0.69↔1.03)	(1.12↔1.61)	(0.54↔1.06)
Ratio of GM (GFP Normalised)	1.000	0.823	0.397	0.641	1.018	0.572
95% CI	(Control)	(0.600↔1.128)	(0.306↔0.515)	(0.512↔0.802)	(0.832↔1.245)	(0.397↔0.823)
Significance Value (Ln Reg)		p=0.2265	p=0.0000 *	p=0.0001 *	p=0.8652	p=0.0027 *
Ratio of GM (IMPα Normalised)		1.000	0.482	0.779	1.000	0.562
95% CI		(Control)	(0.331↔0.702)	(0.548↔1.107)	(Control)	(0.383↔0.824)
Significance Value (Ln Reg)			p=0.0001 *	p=0.1640		p=0.0033 *
J) GM ∑ foci PSPC1-A546 intensity (per cell)	5070	4190	1848	3183	5173	2812
95% CI	(4521↔5686)	(3136↔5597)	(1416↔2412)	(2558↔3960)	(4244↔6305)	(1926↔4104)
Ratio of GM (GFP Normalised)	1.000	0.826	0.364	0.628	1.020	0.555
95% CI	(Control)	(0.588↔1.162)	(0.275↔0.483)	(0.493↔0.800)	(0.820↔1.269)	(0.374↔0.821)
Significance Value (Ln Reg)		p=0.2726	p=0.0000 *	p=0.0002 *	p=0.8568	p=0.0033 *
Ratio of GM (IMPα Normalised)		1.000	0.441	0.760	1.000	0.544
95% CI		(Control)	(0.294↔0.662)	(0.520↔1.11)	(Control)	(0.359↔0.822)
Significance Value (Ln Reg)			p=0.0001 *	p=0.1560		p=0.0039 *
Mean values per PSPC1-A546 nuclear foci	GFP	GFP-IMPα2-FL	GFP-IMPα2ΔIBB	GFP-IMPα2-ED	GFP-IMPα6-FL	GFP-IMPα6ΔIBB
K) GM foci volume (per foci)	0.190	0.209	0.156	0.212	0.215	0.173
95% CI	(0.185↔0.196)	(0.193↔0.227)	(0.145↔0.168)	(0.201↔0.224)	(0.207↔0.224)	(0.157↔0.191)
Ratio of GM (GFP Normalised)	1.000	1.100	0.819	1.114	1.131	0.908
95% CI	(Control)	(0.930↔1.301)	(0.719↔0.932)	(0.903↔1.375)	(1.023↔1.249)	(0.742↔1.113)
Significance Value (GEE)		p=0.2645	p=0.0024 *	p=0.3141	p=0.0158	p=0.3539
Ratio of GM (IMPα Normalised)		1.000	0.744	1.013	1.000	0.804
95% CI		(Control)	(0.613↔0.903)	(0.785↔1.307)	(Control)	(0.652↔0.991)
Significance Value (GEE)			p=0.0028 *	p=0.9225		p=0.041
L) GM foci PSPC1-A546 voxel intensity (per foci)	90.70	91.12	89.32	94.90	92.33	90.31
95% CI	(90.35↔91.06)	(90.07↔92.12)	(88.44↔90.20)	(93.90↔95.91)	(91.71↔92.95)	(89.00↔91.64)
Ratio of GM (GFP Normalised)	1.000	1.005	0.985	1.046	1.018	1.007
95% CI	(Control)	(0.981↔1.03)	(0.968↔1.00)	(0.984↔1.11)	(1.001↔1.04)	(0.978↔1.04)
Significance Value (GEE)		p=0.699	p=0.071	p=0.151	p=0.038	p=0.648
Ratio of GM (IMPα Normalised)		1.000	0.980	1.041	1.000	0.989
95% CI		(Control)	(0.954↔1.01)	(0.976↔1.11)	(Control)	(0.958↔1.022)
Significance Value (GEE)			p=0.15	p=0.22		p=0.5086
M) GM ∑ foci PSPC1-A546 intensity (per foci)	701	792	549	822	815	639
95% CI	(678↔723)	(722↔869)	(505↔597)	(770↔877)	(778↔854)	(571↔716)
Ratio of GM (GFP Normalised)	1.000	1.131	0.783	1.173	1.164	0.927
95% CI	(Control)	(0.933↔1.370)	(0.676↔0.908)	(0.892↔1.542)	(1.036↔1.308)	(0.739↔1.164)
Significance Value (GEE)		p=0.2116	p=0.0012 *	p=0.2539	p=0.0105	p=0.5162
Ratio of GM (IMPα Normalised)		1.000	0.693	1.037	1.000	0.797
95% CI		(Control)	(0.554↔0.867)	(0.752↔1.431)	(Control)	(0.628↔1.010)
Significance Value (GEE)			p=0.0013 *	p=0.8225		p=0.061

The analysed cell numbers for each GFP-tagged IMPα transfection group, the number of detected PSPC1-positive nuclear foci and proportion of cells determined to contain PSPC1 nuclear foci (detected by indirect PSPC1 immunofluorescence with an Alexa Fluor 546 [A546] secondary antibody) are presented. Samples were assessed on a per cell or per PSPC1 nuclear foci basis, with geometric means (GM) and 95% confidence intervals (95% CI) calculated. To determine significant differences between groups, a logistic regression (Lg Reg) model was used for PSPC1 foci positive/negative cells, linear regression (Ln Reg) models were used for per cell data and generalised estimating equations (GEE) were used for per PSPC1 nuclear foci data. Comparative significance values using either GFP or IMPα-FL as the reference group (set at 1.000) are shown. Using Bonforeni correction, the significance threshold was reassigned from ≤0.05 to ≤0.008 (0.05 ÷ 6 experimental groups), with those outcomes below the threshold indicated (*). Further details are provided in Figure 2A with additional samples and analysis parameters included in Supplementary Table S5.

Supplementary Table S3: Outcomes of modulating IMPα expression and transport function on endogenous SFPQ-positive nuclear foci.

	GFP	GFP-IMPα2-FL	GFP-IMPα2ΔIBB	GFP-IMPα2-ED	GFP-IMPα4-FL	GFP-IMPα4ΔIBB	GFP-IMPα6-FL	GFP-IMPα6ΔIBB
A) Number of cells analysed - Total: 2258	566	192	301	143	173	299	273	311
B) SFPQ nuclear foci - Total: 14750	4570	1529	1666	845	1787	1297	2123	933
C) Number of cells +VE for nuclear foci	460	161	174	84	158	180	234	128
D) % Cells +VE for foci	81.3%	83.9%	57.8%	58.7%	91.3%	60.2%	85.7%	41.2%
95% CI	(64.1↔98.4)%	(51.6↔116.1)%	(44.6↔71.0)%	(39.2↔78.3)%	(43.0↔139.7)%	(46.3↔74.1)%	(56.7↔114.8)%	(31.9↔50.5)%
Odds Ratio (GFP Normalised)	1.000	1.197	0.316	0.328	2.427	0.349	1.383	0.161
95% CI	(Control)	(0.772↔1.856)	(0.231↔0.431)	(0.221↔0.487)	(1.373↔4.292)	(0.255↔0.477)	(0.927↔2.061)	(0.118↔0.220)
Significance Value (Lg Reg)		p=0.4217	p=0.0000 *	p=0.0000 *	p=0.0018 *	p=0.0000 *	p=0.1108	p=0.0000 *
Odds Ratio (IMPα Normalised)		1.000	0.264	0.274	1.000	0.144	1.000	0.117
95% CI		(Control)	(0.169↔0.413)	(0.165↔0.456)	(Control)	(0.081↔0.256)	(Control)	(0.078↔0.175)
Significance Value (Lg Reg)			p=0.0000 *	p=0.0000 *		p=0.0000 *		p=0.0000 *
**Mean per cell values (All cells)**	**GFP**	**GFP-IMPα2-FL**	**GFP-IMPα2ΔIBB**	**GFP-IMPα2-ED**	**GFP-IMPα4-FL**	**GFP-IMPα4ΔIBB**	**GFP-IMPα6-FL**	**GFP-IMPα6ΔIBB**
E) GM $F_{n/c}$ per cell - SFPQ-A546	6.24	4.75	3.21	2.80	5.49	3.93	5.32	2.66
95% CI	(6.01↔6.47)	(4.43↔5.09)	(2.93↔3.50)	(2.57↔3.06)	(5.21↔5.79)	(3.70↔4.19)	(5.05↔5.62)	(2.44↔2.90)
Odds Ratio (GFP Normalised)	1.000	0.761	0.514	0.449	0.880	0.631	0.854	0.427
95% CI	(Control)	(0.693↔0.836)	(0.475↔0.557)	(0.405↔0.499)	(0.799↔0.971)	(0.582↔0.683)	(0.786↔0.927)	(0.394↔0.462)
Significance Value (Ln Reg)		p=0.0000 *	p=0.0000 *	p=0.0000 *	p=0.0106	p=0.0000 *	p=0.0002 *	p=0.0000 *
Odds Ratio (IMPα Normalised)		1.000	0.675	0.590	1.000	0.716	1.000	0.500
95% CI		(Control)	(0.609↔0.749)	(0.521↔0.668)	(Control)	(0.643↔0.797)	(Control)	(0.456↔0.549)
Significance Value (Ln Reg)			p=0.0000 *	p=0.0000 *		p=0.0000 *		p=0.0000 *
F) GM intensity per cell - SFPQ-A546	7.13	9.22	6.70	11.04	9.68	7.59	8.55	5.34
95% CI	(6.95↔7.33)	(8.70↔9.77)	(6.29↔7.14)	(9.90↔12.32)	(9.22↔10.16)	(7.24↔9.95)	(8.21↔8.90)	(5.07↔5.63)
Odds Ratio (GFP Normalised)	1.000	1.292	0.939	1.547	1.357	1.063	1.198	0.749
95% CI	(Control)	(1.204↔1.386)	(0.884↔0.997)	(1.430↔1.675)	(1.261↔1.460)	(1.001↔1.129)	(1.126↔1.275)	(0.705↔0.795)
Significance Value (Ln Reg)		p=0.0000 *	p=0.0401	p=0.0000 *	p=0.0000 *	p=0.0467	p=0.0000 *	p=0.0000 *
Odds Ratio (IMPα Normalised)		1.000	0.727	1.198	1.000	0.784	1.000	0.625
95% CI		(Control)	(0.672↔0.786)	(1.091↔1.315)	(Control)	(0.723↔0.849)	(Control)	(0.583↔0.670)
Significance Value (Ln Reg)			p=0.0000 *	p=0.0002 *		p=0.0000 *		p=0.0000 *
G) GM intensity per cell – GFP	13.38	9.34	11.91	8.40	9.42	12.50	12.91	11.00
95% CI	(12.76↔14.04)	(8.63↔10.11)	(11.20↔12.67)	(7.76↔9.10)	(8.67↔10.24)	(11.62↔13.38)	(11.66↔14.28)	(10.17↔11.90)
Odds Ratio (GFP Normalised)	1.000	0.698	0.890	0.628	0.704	0.934	0.964	0.822
95% CI	(Control)	(0.629↔0.773)	(0.815↔0.972)	(0.559↔0.705)	(0.633↔0.784)	(0.855↔1.020)	(0.881↔1.056)	(0.753↔0.897)
Significance Value (Ln Reg)		p=0.0000 *	p=0.0095	p=0.0000 *	p=0.4332	p=0.1283	p=0.4332	p=0.0000 *
Odds Ratio (IMPα Normalised)		1.000	1.276	0.900	1.000	1.326	1.000	0.852
95% CI		(Control)	(1.138↔1.429)	(0.785↔1.031)	(Control)	(1.179↔1.492)	(Control)	(0.769↔0.944)
Significance Value (Ln Reg)			p=0.0000 *	p=0.1286		p=0.0000 *		p=0.0022 *
**Mean per cell values (SFPQ-A546 nuclear foci positive cells only)**	**GFP**	**GFP-IMPα2-FL**	**GFP-IMPα2ΔIBB**	**GFP-IMPα2-ED**	**GFP-IMPα4-FL**	**GFP-IMPα4ΔIBB**	**GFP-IMPα6-FL**	**GFP-IMPα6ΔIBB**
H) GM number of foci (per cell)	7.61	6.25	7.10	7.50	8.13	5.14	6.85	5.25
95% CI	(7.07↔8.20)	(5.39↔7.26)	(6.24↔8.07)	(6.23↔9.01)	(7.08↔9.34)	(4.54↔5.82)	(6.17↔7.60)	(4.51↔6.11)
Odds Ratio (GFP Normalised)	1.000	0.822	0.932	0.985	1.068	0.675	0.900	0.689
95% CI	(Control)	(0.705↔0.958)	(0.803↔1.082)	(0.807↔1.201)	(0.915↔1.246)	(0.583↔0.782)	(0.787↔1.029)	(0.583↔0.815)
Significance Value (Ln Reg)		p=0.012	p=0.358	p=0.879	p=0.406	p=0.0000 *	p=0.123	p=0.0000 *
Odds Ratio (IMPα Normalised)		1.000	1.135	1.199	1.000	0.632	1.000	0.766
95% CI		(Control)	(0.945↔1.363)	(0.957↔1.501)	(Control)	(0.527↔0.759)	(Control)	(0.637↔0.921)
Significance Value (Ln Reg)			p=0.1751	p=0.1152		p=0.0000 *		p=0.0046 *

I) GM ∑ foci volume (per cell)	2.68	1.96	2.44	2.33	3.11	1.16	1.99	1.38
95% CI	(2.33↔3.07)	(1.51↔2.54)	(1.94↔3.06)	(1.70↔3.20)	(2.48↔3.89)	(0.95↔1.41)	(1.65↔2.41)	(1.05↔1.80)
Odds Ratio (GFP Normalised)	1.000	0.731	0.911	0.872	1.161	0.433	0.744	0.514
95% CI	(Control)	(0.558↔0.957)	(0.701↔1.184)	(0.614↔1.237)	(0.884↔1.523)	(0.334↔0.561)	(0.587↔0.943)	(0.382↔0.690)
Significance Value (Ln Reg)		p=0.0228	p=0.4884	p=0.4417	p=0.2828	p=0.0000 *	p=0.0146	p=0.0000 *
Odds Ratio (IMPα Normalised)		1.000	1.247	1.1931	1.000	0.3731	1.000	0.691
95% CI		(Control)	(0.904↔1.722)	(0.802↔1.774)	(Control)	(0.271↔0.515)	(Control)	(0.499↔0.955)
Significance Value (Ln Reg)			p=0.1792	p=0.3835		p=0.0000 *		p=0.0254
J) GM ∑ foci SFPQ-A546 intensity (per cell)	9422	7276	9306	9159	12102	4343	7557	5052
95% CI	(8159↔10879)	(5567↔9509)	(7313↔11843)	(6604↔12703)	(9600↔15255)	(3543↔5322)	(6200↔9212)	(3803↔6712)
Odds Ratio (GFP Normalised)	1.000	0.757	0.968	0.953	1.259	0.452	0.786	0.526
95% CI	(Control)	(0.571↔1.003)	(0.737↔1.273)	(0.662↔1.372)	(0.948↔1.671)	(0.345↔0.592)	(0.614↔1.006)	(0.387↔0.714)
Significance Value (Ln Reg)		p=0.0526	p=0.8172	p=0.7955	p=0.1112	p=0.0000 *	p=0.0563	p=0.0000 *
Odds Ratio (IMPα Normalised)		1.000	1.279	1.259	1.000	0.359	1.000	0.669
95% CI		(Control)	(0.914↔1.790)	(0.833↔1.903)	(Control)	(0.257↔0.502)	(Control)	(0.477↔0.973)
Significance Value (Ln Reg)			p=0.1511	p=0.2753		p=0.0000 *		p=0.0195
Mean values per SFPQ-A546 nuclear foci	GFP	GFP-IMPα2-FL	GFP-IMPα2ΔIBB	GFP-IMPα2-ED	GFP-IMPα4-FL	GFP-IMPα4ΔIBB	GFP-IMPα6-FL	GFP-IMPα6ΔIBB
K) GM foci volume (per foci)	0.318	0.329	0.317	0.286	0.320	0.202	0.264	0.264
95% CI	(0.307↔0.329)	(0.309↔0.349)	(0.300↔0.308)	(0.266↔0.308)	(0.303↔0.337)	(0.191↔0.214)	(0.252↔0.277)	(0.246↔0.283)
Odds Ratio (GFP Normalised)	1.000	1.034	0.997	0.900	1.006	0.636	0.831	0.830
95% CI	(Control)	(0.858↔1.245)	(0.849↔1.170)	(0.760↔1.065)	(0.875↔1.157)	(0.558↔0.724)	(0.732↔0.944)	(0.711↔0.968)
Significance Value (GEE)		p=0.7277	p=0.9697	p=0.2205	p=0.9328	p=0.0000 *	p=0.0044 *	p=0.0178
Odds Ratio (IMPα Normalised)		1.000	0.965	0.871	1.000	0.632	1.000	0.998
95% CI		(Control)	(0.773↔1.204)	(0.693↔1.094)	(Control)	(0.538↔0.741)	(Control)	(0.842↔1.184)
Significance Value (GEE)			p=0.750	p=0.234		p=0.0000 *		p=0.9837
L) GM foci SFPQ-A546 voxel intensity (per foci)	89.56	87.21	89.01	89.26	90.16	87.65	87.48	88.31
95% CI	(89.17↔89.95)	(86.57↔87.85)	(88.32↔89.71)	(88.42↔90.11)	(89.51↔90.82)	(87.08↔88.22)	(86.97↔87.99)	(87.60↔89.03)
Odds Ratio (GFP Normalised)	1.000	0.972	0.995	0.995	1.005	0.977	0.975	0.984
95% CI	(Control)	(0.954↔0.990)	(0.974↔1.016)	(0.976↔1.014)	(0.988↔1.022)	(0.962↔0.991)	(0.962↔0.988)	(0.968↔1.000)
Significance Value (GEE)		p=0.0022 *	p=0.6131	p=0.5925	p=0.5858	p=0.0020 *	p=0.0003 *	p=0.0552
Odds Ratio (IMPα Normalised)		1.000	1.02	1.02	1.000	0.972	1.000	1.009
95% CI		(Control)	(0.998↔1.05)	(0.999↔1.05)	(Control)	(0.953↔0.991)	(Control)	(0.991↔1.03)
Significance Value (GEE)			p=0.0736	p=0.0582		p=0.0045 *		p=0.3070
M) GM ∑ foci SFPQ-A546 intensity (per foci)	1107	1202	1188	1071	1204	728	964	963
95% CI	(1068↔1149)	(1126↔1282)	(1117↔1263)	(988↔1160)	(1135↔1278)	(684↔775)	(914↔1017)	(891↔1041)
Odds Ratio (GFP Normalised)	1.000	1.082	1.074	0.964	1.084	0.655	0.868	0.867
95% CI	(Control)	(0.890↔1.315)	(0.900↔1.281)	(0.807↔1.151)	(0.931↔1.262)	(0.568↔0.755)	(0.759↔0.992)	(0.736↔1.022)
Significance Value (GEE)		p=0.430	p=0.429	p=0.684	p=0.0808	p=0.0000 *	p=0.038	p=0.089
Odds Ratio (IMPα Normalised)		1.000	0.993	0.891	1.000	0.604	1.000	0.999
95% CI		(Control)	(0.782↔1.260)	(0.701↔1.132)	(Control)	(0.507↔0.720)	(Control)	(0.835↔1.196)
Significance Value (GEE)			p=0.952	p=0.344		p=0.0000 *		p=0.9931

The analysed cell numbers for each GFP-tagged IMPα transfection group, the number of detected SFPQ-positive nuclear foci and proportion of cells determined to contain SFPQ nuclear foci (detected by indirect SFPQ immunofluorescence with an Alexa Fluor 546 [A546] secondary antibody) are presented. Samples were assessed on a per cell or per SFPQ nuclear foci basis, with geometric means (GM) and 95% confidence intervals (95% CI) calculated. To determine significant differences between groups, a logistic regression (Lg Reg) model was used for SFPQ foci positive/negative cells, linear regression (Ln Reg) models were used for per cell data and generalised estimating equations (GEE) were used for per PSPC1 nuclear foci data. Comparative significance values using either GFP or IMPα-FL as the reference group (set at 1.000) are shown. Using Bonforeni correction, the significance threshold was reassigned from ≤0.05 to ≤0.0063 (0.05 ÷ 8 experimental groups), with those outcomes below the threshold indicated (*). Further details are provided in Figure 2C with additional samples and analysis parameters included in Supplementary Table S6.

Supplementary Table S4: Outcomes of modulating IMPα expression and transport function on exogenous dsRed2-PSPC1-positive nuclear foci.

	GFP	GFP-IMPα2-FL	GFP-IMPα2ΔIBB	GFP-IMPα2-ED	GFP-IMPα4-FL	GFP-IMPα4ΔIBB	GFP-IMPα6-FL	GFP-IMPα6ΔIBB
A) Number of cells analysed - Total: 2530	180	228	241	446	317	386	513	219
B) DsRed2-PSPC1 nuclear foci - Total: 38657	2508	3392	2147	6218	10853	4339	5473	3745
C) Number of cells +VE for nuclear foci	50	117	92	195	189	143	195	87
D) % Cells +VE for foci	27.8%	51.3%	38.2%	43.7%	59.6%	37%	38%	39.7%
95% CI	(18.7↔36.8)%	(38.0↔64.6)%	(28.3↔48.1)%	(35.5↔51.9)%	(46.2↔73.0)%	(29.4↔44.7)%	(31.2↔44.8)%	(29.0↔50.5)%
Odds Ratio (GFP Normalised)	1.000	2.741	1.605	2.020	3.839	1.530	1.594	1.714
95% CI	(Control)	(1.806↔4.158)	(1.058↔2.436)	(1.387↔2.942)	(2.584↔5.704)	(1.040↔2.251)	(1.099↔2.312)	(1.122↔2.618)
Significance Value (Lg Reg)		p=0.0000 *	p=0.0256	p=0.0002 *	p=0.0000 *	p=0.0303	p=0.0135	p=0.0124
Odds Ratio (IMPα Normalised)		1.000	0.586	0.737	1.000	0.399	1.000	1.075
95% CI		(Control)	(0.406↔0.846)	(0.535↔1.015)	(Control)	(0.294↔0.541)	(Control)	(0.777↔1.486)
Significance Value (Lg Reg)			p=0.0042 *	p=0.0614		p=0.0000 *		p=0.6625
**Mean per cell values (All cells)**	**GFP**	**GFP-IMPα2-FL**	**GFP-IMPα2ΔIBB**	**GFP-IMPα2-ED**	**GFP-IMPα4-FL**	**GFP-IMPα4ΔIBB**	**GFP-IMPα6-FL**	**GFP-IMPα6ΔIBB**
E) GM $F_{n/c}$ per cell - DsRed2-PSPC1	1.66	1.86	1.62	1.60	1.94	1.78	1.56	1.86
95% CI	(1.57↔1.76)	(1.75↔1.98)	(1.53↔1.72)	(1.54↔1.66)	(1.82↔2.06)	(1.70↔1.87)	(1.51↔1.62)	(1.72↔3.62)
Odds Ratio (GFP Normalised)	1.000	1.120	0.977	0.962	1.165	1.074	0.940	1.120
95% CI	(Control)	(1.020↔1.227)	(0.891↔1.070)	(0.887↔1.045)	(1.068↔1.270)	(0.987↔1.168)	(0.867↔1.018)	(1.020↔1.229)
Significance Value (Ln Reg)		p=0.0177	p=0.6122	p=0.3600	p=0.0006 *	p=0.0967	p=0.1323	p=0.0179
Odds Ratio (IMPα Normalised)		1.000	0.873	0.860	1.000	0.922	1.000	1.191
95% CI		(Control)	(0.801↔0.951)	(0.798↔0.928)	(Control)	(0.859↔0.989)	(Control)	(1.105↔1.284)
Significance Value (Ln Reg)			p=0.0019 *	p=0.0001 *		p=0.0235		p=0.0000 *
F) GM intensity per cell - DsRed2-PSPC1	5.78	5.85	4.28	5.19	6.38	4.95	4.40	4.59
95% CI	(5.57↔6.00)	(5.66↔6.06)	(4.123↔4.434)	(4.99↔5.40)	(6.12↔6.65)	(4.76↔5.15)	(4.28↔4.53)	(4.38↔4.81)
Odds Ratio (GFP Normalised)	1.000	1.013	0.740	0.898	1.103	0.857	0.762	0.794
95% CI	(Control)	(0.945↔1.086)	(0.691↔0.792)	(0.844↔0.955)	(1.034↔1.178)	(0.804↔0.912)	(0.717↔0.809)	(0.741↔0.852)
Significance Value (Ln Reg)		p=0.7230	p=0.0000 *	p=0.0006 *	p=0.0031 *	p=0.0000 *	p=0.0000 *	p=0.0000 *
Odds Ratio (IMPα Normalised)		1.000	0.730	0.887	1.000	0.777	1.000	1.042
95% CI		(Control)	(0.685↔0.779)	(0.838↔0.939)	(Control)	(0.737↔0.819)	(Control)	(0.986↔1.103)
Significance Value (Ln Reg)			p=0.0000 *	p=0.0000 *		p=0.0000 *		p=0.144
G) GM intensity per cell – GFP	24.51	11.10	18.05	13.04	10.59	14.31	15.74	14.46
95% CI	(21.6↔27.9)	(10.30↔11.96)	(16.39↔19.89)	(12.29↔13.84)	(9.99↔11.22)	(13.25↔15.47)	(14.67↔16.88)	(13.05↔16.01)
Odds Ratio (GFP Normalised)	1.000	0.453	0.737	0.532	0.432	0.584	0.642	0.590
95% CI	(Control)	(0.393↔0.522)	(0.640↔0.847)	(0.469↔0.603)	(0.378↔0.493)	(0.514↔0.664)	(0.568↔0.726)	(0.511↔0.680)
Significance Value (Ln Reg)		p=0.0000 *	p=0.0000 *	p=0.0000 *	p=0.0000 *	p=0.0000 *	p=0.0000 *	p=0.0000 *
Odds Ratio (IMPα Normalised)		1.000	1.627	1.175	1.000	1.352	1.000	0.919
95% CI		(Control)	(1.427↔1.855)	(1.047↔1.139)	(Control)	(1.214↔1.505)	(Control)	(0.819↔1.030)
Significance Value (Ln Reg)			p=0.0000 *	p=0.0062 *		p=0.0000 *		p=0.1462
**Mean per cell values (DsRed2-PSPC1 nuclear foci positive cells only)**	**GFP**	**GFP-IMPα2-FL**	**GFP-IMPα2ΔIBB**	**GFP-IMPα2-ED**	**GFP-IMPα4-FL**	**GFP-IMPα4ΔIBB**	**GFP-IMPα6-FL**	**GFP-IMPα6ΔIBB**
H) GM number of foci (per cell)	12.70	11.51	7.49	10.32	14.94	7.78	9.04	15.58
95% CI	(7.88↔20.48)	(9.04↔14.65)	(5.53↔10.13)	(8.39↔12.70)	(11.70↔19.07)	(5.96↔10.15)	(7.33↔11.14)	(11.30↔21.50)
Odds Ratio (GFP Normalised)	1.000	0.906	0.589	0.813	1.176	0.613	0.711	1.227
95% CI	(Control)	(0.543↔1.511)	(0.346↔1.004)	(0.503↔1.314)	(0.726↔1.904)	(0.372↔1.008)	(0.440↔1.150)	(0.717↔2.101)
Significance Value (Ln Reg)		p=0.7051	p=0.0518	p=0.3973	p=0.5095	p=0.0539	p=0.1649	p=0.4558
Odds Ratio (IMPα Normalised)		1.000	0.651	0.897	1.000	0.521	1.000	1.725
95% CI		(Control)	(0.427↔0.992)	(0.629↔1.278)	(Control)	(0.372↔0.729)	(Control)	(1.167↔2.549)
Significance Value (Ln Reg)			p=0.0462	p=0.5481		p=0.0001 *		p=0.0063*

I) GM ∑ foci volume (per cell)	2.15	1.88	1.03	1.43	2.48	1.00	1.29	2.59
95% CI	(1.13↔4.08)	(1.37↔2.53)	(0.71↔1.51)	(1.10↔1.86)	(1.79↔3.42)	(0.71↔1.42)	(0.97↔1.71)	(1.71↔3.91)
Odds Ratio (GFP Normalised)	1.000	0.876	0.481	0.666	1.153	0.467	0.601	1.204
95% CI	(Control)	(0.448↔1.713)	(0.239↔0.967)	(0.355↔1.250)	(0.613↔2.169)	(0.243↔0.896)	(0.320↔1.128)	(0.595↔2.437)
Significance Value (Ln Reg)		p=0.6982	p=0.0402	p=0.2065	p=0.6587	p=0.0222	p=0.1135	p=0.6053
Odds Ratio (IMPα Normalised)		1.000	0.550	0.761	1.000	0.405	1.000	2.003
95% CI		(Control)	(0.316↔0.956)	(0.478↔1.211)	(Control)	(0.261↔0.628)	(Control)	(1.200↔3.343)
Significance Value (Ln Reg)			p=0.0342	p=0.2490		p=0.0001 *		p=0.0080
J) GM ∑ foci DsRed2-PSPC1 intensity (per cell)	8724	7704	3921	5499	10240	3838	5102	10400
95% CI	(4397↔17310)	(5476↔10840)	(2602↔5908)	(4152↔7283)	(7250↔14460)	(2649↔5562)	(3744↔6954)	(6684↔16190)
Odds Ratio (GFP Normalised)	1.000	0.883	0.449	0.630	1.173	0.440	0.585	1.193
95% CI	(Control)	(0.430↔1.814)	(0.213↔9.50)	(0.321↔1.238)	(0.596↔2.311)	(0.218↔8.859)	(0.298↔1.149)	(0.560↔2.540)
Significance Value (Ln Reg)		p=0.7349	p=0.0365	p=0.181	p=0.6437	p=0.0217	p=0.1198	p=0.6482
Odds Ratio (IMPα Normalised)		1.000	0.509	0.714	1.000	0.375	1.000	2.039
95% CI		(Control)	(0.281↔9.214)	(0.434↔1.175)	(Control)	(0.234↔6.012)	(Control)	(1.177↔3.532)
Significance Value (Ln Reg)			p=0.0259	p=0.1850		p=0.0001 *		p=0.0112
**Mean values per DsRed2-PSPC1 nuclear foci**	**GFP**	**GFP-IMPα2-FL**	**GFP-IMPα2ΔIBB**	**GFP-IMPα2-ED**	**GFP-IMPα4-FL**	**GFP-IMPα4ΔIBB**	**GFP-IMPα6-FL**	**GFP-IMPα6ΔIBB**
K) GM foci volume (per foci)	0.215	0.162	0.159	0.155	0.229	0.179	0.197	0.180
95% CI	(0.207↔0.224)	(0.157↔0.167)	(0.152↔0.166)	(0.151↔0.159)	(0.225↔0.234)	(0.174↔0.185)	(0.192↔0.202)	(0.174↔0.185)
Odds Ratio (GFP Normalised)	1.000	0.753	0.738	0.720	1.065	0.834	0.916	0.835
95% CI	(Control)	(0.593↔0.955)	(0.519↔1.049)	(0.561↔0.924)	(0.835↔1.360)	(0.646↔1.077)	(0.720↔1.165)	(0.627↔1.112)
Significance Value (GEE)		p=0.0195	p=0.0903	p=0.0097	p=0.6124	p=0.1643	p=0.4734	p=0.2164
Odds Ratio (IMPα Normalised)		1.000	0.980	0.956	1.000	0.783	1.000	0.911
95% CI		(Control)	(0.710↔1.353)	(0.779↔1.175)	(Control)	(0.629↔0.975)	(Control)	(0.709↔1.172)
Significance Value (GEE)			p=0.9026	p=0.6707		p=0.0290		p=0.469
L) GM foci DsRed2-PSPC1 voxel intensity (per foci)	99.89	95.18	97.14	92.37	102.8	95.52	102.1	97.08
95% CI	(98.84↔100.9)	(94.42↔95.94)	(95.95↔98.34)	(91.83↔92.92)	(102.3↔103.4)	(94.76↔96.29)	(101.3↔102.9)	(96.22↔97.94)
Odds Ratio (GFP Normalised)	1.000	0.953	0.975	0.925	1.029	0.956	1.022	0.972
95% CI	(Control)	(0.883↔1.028)	(0.873↔1.088)	(0.857↔0.998)	(0.944↔1.122)	(0.877↔1.024)	(0.943↔1.108)	(0.886↔1.066)
Significance Value (GEE)		p=0.211	p=0.647	p=0.045	p=0.511	p=0.309	p=0.590	p=0.544
Odds Ratio (IMPα Normalised)		1.000	1.023	0.971	1.000	0.929	1.000	0.951
95% CI		(Control)	(0.931↔1.12)	(0.924↔1.02)	(Control)	(0.861↔1.002)	(Control)	(0.880↔1.026)
Significance Value (GEE)			p=0.637	p=0.232		p=0.0565		p=0.1947
M) GM ∑ foci DsRed2-PSPC1 intensity (per foci)	885	614	613	577	970	694	820	707
95% CI	(845↔928)	(590↔638)	(581↔647)	(560↔594)	(946↔994)	(669↔720)	(793↔848)	(679↔736)
Odds Ratio (GFP Normalised)	1.000	0.693	0.694	0.651	1.095	0.784	0.926	0.798
95% CI	(Control)	(0.506↔0.948)	(0.430↔1.123)	(0.469↔0.905)	(0.785↔1.528)	(0.557↔1.104)	(0.670↔1.279)	(0.544↔1.172)
Significance Value (GEE)		p=0.022	p=0.137	p=0.011	p=0.593	p=0.163	p=0.641	p=0.251
Odds Ratio (IMPα Normalised)		1.000	1.00	0.94	1.000	0.716	1.000	0.862
95% CI		(Control)	(0.649↔1.55)	(0.726↔1.22)	(Control)	(0.532↔0.963)	(Control)	(0.617↔1.205)
Significance Value (GEE)			p=0.991	p=0.6383		p=0.0270		p=0.385

The analysed cell numbers for each GFP-tagged IMPα group, all co-transfected with DsRed2-PSPC1, the number of detected DsRed2-PSPC1-positive nuclear foci and proportion of cells determined to contain DsRed2-PSPC1 nuclear foci (detected by DsRed2-PSPC1 fluorescence) are presented. Samples were assessed on a per cell or per DsRed2-PSPC1 nuclear foci basis, with geometric means (GM) and 95% confidence intervals (95% CI) calculated. To determine significant differences between groups, a logistic regression (Lg Reg) model was used for DsRed2-PSPC1 foci positive/negative cells, linear regression (Ln Reg) models were used for per cell data and generalised estimating equations (GEE) were used for per DsRed2-PSPC1 nuclear foci data. Comparative significance values using either GFP or IMPα-FL as the reference group (set at 1.000) are shown. Using Bonforeni correction, the significance threshold was reassigned from ≤0.05 to ≤0.0063 (0.05 ÷ 8 experimental groups), with those outcomes below the threshold indicated (*). Further details are provided in Figure 2B with additional samples and analysis parameters included in Supplementary Table S7.

Supplementary Table S5: Detailed outcomes of modulating IMPα expression and transport function on endogenous PSPC1-positive nuclear foci

8C-S-PSPC1_V2.00	Not-Trans	Not-Trans-C	Mock	Mock-C	ALL-GFP	ALL-GFP-C	GFP	GFP-1	GFP-2	GFP-IMPa2-FL	GFP-IMPa2-IBB	GFP-IMPa2-ED	GFP-IMPa4-IBB	GFP-IMPa6-FL	GFP-IMPa6-IBB	
SortOrder	1	1	4	4	6	6	7	8	9	10	11	12	14	15	16	Additional Limits
Nuclei PRE X, Y & Z Exclusions	2898	2898	933	933	2542	2542	2162	1353	809	1146	1739	1399	1276	917	894	
CountExcludeImgBound.x	184	184	24	24	127	127	112	86	26	46	82	93	80	43	24	
CountExcludeImgBound.y	149	149	28	28	111	111	99	70	29	66	78	91	59	51	23	
CountExcludeImgBound.z	13	13	507	507	37	37	451	206	245	570	583	197	20	134	379	
CountExcludeImgBound	336	336	537	537	270	270	632	341	291	644	700	344	152	212	414	
Nuclei POST X, Y & Z Exclusions	2562	2562	396	396	2272	2272	1530	1012	518	502	1039	1055	1124	705	480	
CountPreGFPLimit	2962	2962	1066	1066	2689	2689	2326	1437	889	1301	1869	1497	1609	1092	1153	
CountPostGFPLimit	31	2962	16	1066	588	2689	1291	770	521	476	1029	631	790	661	528	
Cells removed on IMP Limit	NA	NA	NA	NA	NA	NA	NA	NA	NA	NA	NA	NA	NA	NA	NA	
Number of Cells (Nuclei)	29	2562	3	396	467	2272	813	514	299	166	519	459	550	431	214	Per Sample
Number Cells (Nuclei) +VE for Foci	18	1996	NA	123	1	3	598	430	168	91	144	211	151	285	66	
Number Cells (Nuclei) -VE for Foci	11	566	3	273	466	2269	215	84	131	75	375	248	399	146	148	
**% Cells (Nuclei) Positive for Foci**	**62.1**	**77.9**	**NA**	**31.1**	**0.2**	**0.1**	**73.6**	**83.7**	**56.2**	**54.8**	**27.7**	**46.0**	**27.5**	**66.1**	**30.8**	
Number of Nuclear Foci	147	14023	0	1200	85	92	5079	3753	1326	595	728	1461	815	2597	405	
Number of Cells (Cells)	29	2560	3	396	467	2270	813	514	299	166	519	459	550	431	214	
Number Cells (Cells) +VE cytoplasmic Foci	NA	26	NA	7	2	5	62	29	33	7	8	5	8	14	7	
Number of Cells (Cells) -VE cytoplasmic Foci	29	2536	3	389	465	2267	751	485	266	159	511	454	542	417	207	
% Cells (Cells) with cytoplasmic Foci	NA	1.01	NA	1.77	0.428	0.22	7.63	5.64	11	4.22	1.54	1.09	1.45	3.25	3.27	
Number of Cytoplasmic Foci	0	84	0	16	2	36	153	60	93	14	14	14	15	44	14	
GM Cytoplasmic Int (Cells) - PSPC1-A546	7.84	6.46	5.09	6.3	5.8	5.73	6.45	6.57	6.23	5.83	5.94	6.38	5.59	6.37	5.84	Per Cell (Sample Wide)
GM Cytoplasmic Int (Cells) - GFP-IMPs	5.59	4.59	4.68	4.55	8.72	3.97	14.1	14.4	13.4	8.43	9.28	5.66	9.1	11.2	9.03	
GM Cytoplasmic Int (Cells) - DRAQ5	21.5	14.7	13.1	13.5	11.9	13	10.7	11.3	9.57	11.5	12.2	16.6	12.9	11.7	11	
GM Nucleus Int (Nuclei) - PSPC1-A546	16	17	7.12	11.3	7.58	6.59	19.9	21.9	16.9	14.3	10.8	12.2	10.6	18.2	11.4	
GM Nucleus Int (Nuclei) - GFP-IMPs	7.85	4.68	8.32	4.71	36.5	6.34	41.4	40.5	43.1	22.2	43.5	17.8	48.6	41.6	31.7	
GM Nucleus Int (Nuclei) - DRAQ5	77.8	55.5	50.7	45.1	46.9	46.1	47.9	53.1	40.1	54.1	48.7	64.8	51	50.4	42.1	
fNC Inf nCells (Count) - PSPC1-A546	NA	NA	NA	NA	NA	21	NA	NA	NA	NA	NA	NA	NA	NA	NA	
fNC Inf nCells (Count) - GFP-IMPs	NA	NA	NA	NA	NA	21	NA	NA	NA	NA	NA	NA	NA	NA	NA	
fNC Inf nCells (Count) - DRAQ5	NA	NA	NA	NA	NA	21	NA	NA	NA	NA	NA	NA	NA	NA	NA	
**GM fNC per Cell (N&C) - PSPC1-A546**	**2.05**	**2.63**	**1.4**	**1.79**	**1.31**	**1.14**	**3.09**	**3.33**	**2.72**	**2.45**	**1.83**	**1.92**	**1.9**	**2.85**	**1.95**	
GM fNC per Cell (N&C) - GFP-IMPs	1.41	1.02	1.78	1.03	4.18	1.59	2.95	2.8	3.21	2.63	4.68	3.14	5.34	3.72	3.52	
GM fNC per Cell (N&C) - DRAQ5	3.61	3.78	3.86	3.34	3.94	3.47	4.5	4.68	4.19	4.7	3.99	3.91	3.95	4.32	3.82	
**GM Int per Cell (C&N) - PSPC1-A546**	**12.5**	**11.8**	**5.93**	**8.51**	**6.13**	**5.86**	**8.77**	**9.45**	**7.71**	**7.66**	**7.16**	**9.09**	**6.83**	**8.79**	**6.82**	
GM Int per Cell (C&N) - GFP-IMPs	6.96	4.62	6.42	4.59	14.3	4.49	18.4	19.2	17	11.4	17.2	11.1	18.3	17.2	12.7	
GM Int per Cell (C&N) - DRAQ5	51.1	35	27.8	25.5	18.9	22.8	16.9	19.3	13.5	20.2	20.6	37.6	21.7	19.5	16.1	
**GM number of nuclear Foci per cell (Nuclei)**	**4.89**	**4.78**	**NA**	**5.79**	**85**	**7.99**	**6.2**	**6.38**	**5.75**	**4.84**	**3.52**	**4.68**	**3.7**	**6**	**4.41**	Per Cell (Foci +VE)
GM number of nuclear Foci per cell (Cell)	4.89	4.78	NA	5.79	85	7.99	6.2	6.38	5.75	4.84	3.52	4.68	3.7	6	4.41	
GM number of cytoplasmic Foci per cell (Cell)	NA	2.09	NA	1.87	1	3.59	1.78	1.7	1.85	1.46	1.45	1.97	1.62	2.05	1.63	
**GM Sum volume of nuclear Foci per cell (Cell)**	**0.991**	**0.739**	**NA**	**1.07**	**174**	**3.27**	**1.32**	**1.32**	**1.3**	**1.08**	**0.523**	**0.845**	**0.605**	**1.34**	**0.754**	
GM Sum volume of cytoplasmic Foci per cell (Cell)	NA	0.28	NA	0.165	0.0723	1.58	0.219	0.213	0.224	0.194	0.409	0.302	0.313	0.175	0.188	
**GM Sum Nuclear Foci Int per Cell (Cell) - PSPC1-A546**	**3720**	**2800**	**NA**	**4110**	**1360000**	**18700**	**5070**	**5110**	**4970**	**4190**	**1850**	**3180**	**2170**	**5170**	**2810**	
GM Sum Nuclear Foci Int per Cell (Cell) - GFP-IMPs	322	179	NA	261	158000	1450	3450	3370	3660	1290	1540	1190	1870	3430	1370	
GM Sum Nuclear Foci Int per Cell (Cell) - DRAQ5	4730	3120	NA	3660	1030000	14200	4830	5320	3770	4770	1800	4120	2270	4930	2500	
GM Sum Cytoplasmic Foci Int per Cell (Cell) - PSPC1-A546	NA	1030	NA	591	281	8450	783	732	831	626	1490	1130	1280	522	584	
GM Sum Cytoplasmic Foci Int per Cell (Cell) - GFP-IMPs	NA	61.3	NA	46.5	14.4	382	471	519	433	80	1100	140	320	278	222	
GM Sum Cytoplasmic Foci Int per Cell (Cell) - DRAQ5	NA	891	NA	396	195	6030	433	473	400	477	1040	635	761	361	285	
**GM Nuclear Foci Volume**	**0.225**	**0.158**	**NA**	**0.198**	**1.51**	**1.38**	**0.19**	**0.185**	**0.207**	**0.209**	**0.156**	**0.212**	**0.165**	**0.215**	**0.173**	Per Foci
**GM Nuclear Foci Int - PSPC1-A546**	**90.3**	**91.6**	**NA**	**92.9**	**190**	**187**	**90.7**	**91**	**89.8**	**91.1**	**89.3**	**94.9**	**87.8**	**92.3**	**90.3**	
GM Nuclear Foci Int - GFP-IMPs	7.74	5.67	NA	5.32	11	10.7	60.4	57.3	70.1	26.6	75.8	36.2	73.5	54.8	43.6	
GM Nuclear Foci Int - DRAQ5	127	99.5	NA	77	139	138	86.1	93.6	68.1	105	86.3	122	89.3	85.1	78.6	
**GM Sum Nuclear Foci Int - PSPC1-A546**	**836**	**584**	**NA**	**756**	**12500**	**11200**	**701**	**678**	**767**	**792**	**549**	**822**	**584**	**815**	**639**	
GM Sum Nuclear Foci Int - GFP-IMPs	71.6	36.1	NA	43.3	720	640	466	427	598	231	465	313	489	484	309	
GM Sum Nuclear Foci Int - DRAQ5	1180	634	NA	627	9150	8240	665	698	581	910	530	1060	595	752	556	
GM Cytoplasmic Foci Volume	NA	0.185	NA	0.0835	0.0723	0.884	0.128	0.12	0.134	0.107	0.314	0.119	0.173	0.109	0.122	
GM Cytoplasmic Foci Int - PSPC1-A546	NA	103	NA	81.5	81.2	166	90.5	91	90.2	83.1	101	84.8	98	84.1	87.8	
GM Cytoplasmic Foci Int - GFP-IMPs	NA	6.04	NA	5.37	4.15	5.68	62	75.4	54.6	8.89	105	10.1	20	35.4	22.8	
GM Cytoplasmic Foci Int - DRAQ5	NA	79.9	NA	50.1	56.3	115	49.5	54.9	46.2	72.6	72.4	43.9	51.5	53.8	38.2	
GM Sum Cytoplasmic Foci Int - PSPC1-A546	NA	739	NA	286	281	6300	454	405	489	342	1300	437	742	357	392	
GM Sum Cytoplasmic Foci Int - GFP-IMPs	NA	43.5	NA	18.9	14.4	215	311	336	296	36.6	1350	51.8	151	150	102	
GM Sum Cytoplasmic Foci Int - DRAQ5	NA	574	NA	176	195	4380	248	244	250	299	929	226	390	229	171	

Outcomes are labelled (rows) as integers or geometric means (GM) for each experimental group (columns). Groups with the " C" suffix are control groups and are copies of their compliment group without GFP-thresholding applied. GFP-tagged importin transient transfection groups are all as indicated, while "GFP" group is a merge of the two GFP-alone groups "GFP-1" and "GFP-2", which showed no obviously disenable differences. Other control groups included were not transfected cells "Not-Trans", mock transfected cells (without plasmid DNA) "Mock-Trans" and secondary antibody only control images from all of the other experiment groups "ALL-GFP".

Supplementary Table S6: Detailed outcomes of modulating IMPα expression and transport function on endogenous SFPQ-positive nuclear foci.

8C-S-SFPQ_V2.00	Not-Trans	Not-Trans-C	Mock	Mock-C	ALL-GFP	ALL-GFP-C	GFP	GFP-1	GFP-2	GFP-IMPa2-FL	GFP-IMPa2-IBB	GFP-IMPa2-ED	GFP-IMPa4-FL	GFP-IMPa4-IBB	GFP-IMPa6-FL	GFP-IMPa6-IBB	
SortOrder	1	1	4	4	6	6	7	8	9	10	11	12	13	14	15	16	Additional Limits
Nuclei PRE X, Y & Z Exclusions	2114	2114	5873	5873	2542	2542	1601	1038	563	2185	889	941	1530	1133	935	1386	
CountExcludeImgBound.x	90	90	251	251	127	127	63	46	17	170	33	47	70	72	23	58	
CountExcludeImgBound.y	97	97	295	295	111	111	74	44	30	142	53	36	97	66	40	75	
CountExcludeImgBound.z	271	271	1318	1318	37	37	NA	139	NA	59	3	196	9	NA	NA	115	
CountExcludeImgBound	422	422	1771	1771	270	270	270	223	47	354	88	262	167	136	62	236	
Nuclei POST X, Y & Z Exclusions	1692	1692	4102	4102	2272	2272	1331	815	516	1831	801	679	1363	997	873	1150	
CountPreGFPLimit	2123	2123	5892	5892	2689	2689	1820	1157	663	2228	980	981	1640	1219	1039	1491	
CountPostGFPLimit	NA	2123	6	5892	588	2689	734	434	300	234	355	251	211	365	325	435	
Cells removed on IMP Limit	NA	NA	NA	NA	NA	NA	NA	NA	NA	NA	NA	NA	NA	NA	NA	NA	
Number of Cells (Nuclei)	NA	1692	5	4102	467	2272	566	311	255	192	301	143	173	299	273	311	Per Sample
Number Cells (Nuclei) +VE for Foci	NA	1513	4	2569	1	3	460	281	179	161	174	84	158	180	234	128	
Number Cells (Nuclei) -VE for Foci	NA	179	1	1533	466	2269	106	30	76	31	127	59	15	119	39	183	
**% Cells (Nuclei) Positive for Foci**	**NA**	**89.4**	**80.0**	**62.6**	**0.2**	**0.1**	**81.3**	**90.4**	**70.2**	**83.9**	**57.8**	**58.7**	**91.3**	**60.2**	**85.7**	**41.2**	
Number of Nuclear Foci	NA	11888	37	15240	85	92	4570	2882	1688	1529	1666	845	1787	1297	2123	933	
Number of Cells (Cells)	NA	1690	5	4100	467	2270	566	311	255	192	301	143	173	299	273	311	
Number Cells (Cells) +VE cytoplasmic Foci	NA	7	NA	17	2	5	53	32	21	7	13	2	14	7	40	5	
Number of Cells (Cells) -VE cytoplasmic Foci	NA	1685	5	4085	465	2267	513	279	234	185	288	141	159	292	233	306	
% Cells (Cells) with cytoplasmic Foci	NA	0.414	NA	0.414	0.428	0.22	9.36	10.3	8.24	3.65	4.32	1.4	8.09	2.34	14.7	1.61	
Number of Cytoplasmic Foci	NA	13	0	49	2	36	237	172	65	16	62	3	76	42	399	7	
GM Cytoplasmic Int (Cells) - SFPQ-A546	NA	3.24	4.11	3.94	5.8	5.73	4.35	4.71	3.95	3.95	4.13	5.37	4.61	4.19	4.93	3.7	Per Cell (Sample Wide)
GM Cytoplasmic Int (Cells) - GFP-IMPs	NA	2.71	5.13	4.03	8.72	3.97	10.3	10.4	10.3	6.62	6.83	4.82	6.63	6.11	8.58	7.54	
GM Cytoplasmic Int (Cells) - DRAQ5	NA	7.31	35.3	18.6	11.9	13	8.76	9.14	8.31	14.8	11.4	17.3	12.3	12.8	10.6	11.9	
GM Nucleus Int (Nuclei) - SFPQ-A546	NA	25.1	10.5	15.9	7.58	6.59	27.1	31.7	22.4	18.8	13.2	15	25.3	16.5	26.2	9.86	
GM Nucleus Int (Nuclei) - GFP-IMPs	NA	3.77	6.75	3.88	36.5	6.34	35.7	33.8	38.1	14	28.2	11.3	17.9	30.2	31.4	23.2	
GM Nucleus Int (Nuclei) - DRAQ5	NA	47.5	75.7	66.3	46.9	46.1	40.7	41.4	39.9	75.8	48.2	53.1	55	47.3	44.1	48.5	
fNC Inf nCells (Count) - SFPQ-A546	NA	540	NA	257	NA	21	NA	NA	NA	NA	NA	NA	NA	NA	NA	NA	
fNC Inf nCells (Count) - GFP-IMPs	NA	540	NA	257	NA	21	NA	NA	NA	NA	NA	NA	NA	NA	NA	NA	
fNC Inf nCells (Count) - DRAQ5	NA	540	NA	257	NA	21	NA	NA	NA	NA	NA	NA	NA	NA	NA	NA	
**GM fNC per Cell (N&C) - SFPQ-A546**	**NA**	**4.55**	**2.55**	**3.65**	**1.31**	**1.14**	**6.24**	**6.73**	**5.69**	**4.75**	**3.21**	**2.8**	**5.49**	**3.93**	**5.32**	**2.66**	
GM fNC per Cell (N&C) - GFP-IMPs	NA	0.889	1.32	0.882	4.18	1.59	3.45	3.27	3.69	2.12	4.13	2.35	2.7	4.94	3.66	3.08	
GM fNC per Cell (N&C) - DRAQ5	NA	2.67	2.14	2.97	3.94	3.47	4.65	4.53	4.8	5.11	4.24	3.07	4.46	3.68	4.15	4.07	
**GM Int per Cell (C&N) - SFPQ-A546**	**NA**	**24.2**	**9.05**	**14.5**	**6.13**	**5.86**	**7.14**	**8.17**	**6.06**	**9.22**	**6.7**	**11**	**9.68**	**7.59**	**8.55**	**5.34**	
GM Int per Cell (C&N) - GFP-IMPs	NA	3.79	6.36	3.95	14.3	4.49	13.4	13.5	13.3	9.34	11.9	8.4	9.42	12.5	12.9	11	
GM Int per Cell (C&N) - DRAQ5	NA	46	65.7	60.1	18.9	22.8	12.6	13.4	11.7	36.5	20.3	37	23.2	21.9	16.5	20	
**GM number of nuclear Foci per cell (Nuclei)**	**NA**	**5.86**	**5.34**	**4.48**	**85**	**7.99**	**7.61**	**7.9**	**7.19**	**6.25**	**7.1**	**7.5**	**8.13**	**5.14**	**6.85**	**5.25**	Per Cell (Foci +VE)
GM number of nuclear Foci per cell (Cell)	NA	5.86	5.34	4.48	85	7.99	7.61	7.9	7.19	6.25	7.1	7.5	8.13	5.14	6.85	5.25	
GM number of cytoplasmic Foci per cell (Cell)	NA	1.64	NA	1.8	1	3.59	2.91	3.45	2.24	2.03	2.22	1.41	3.16	2.72	3.97	1.32	
**GM Sum volume of nuclear Foci per cell (Cell)**	**NA**	**1.12**	**2.05**	**0.916**	**174**	**3.27**	**2.68**	**2.89**	**2.37**	**1.96**	**2.44**	**2.33**	**3.11**	**1.16**	**1.99**	**1.38**	
GM Sum volume of cytoplasmic Foci per cell (Cell)	NA	0.181	NA	0.221	0.0723	1.58	0.506	0.626	0.367	0.276	0.346	0.342	0.612	0.383	0.785	0.148	
**GM Sum Nuclear Foci Int per Cell (Cell) - SFPQ-A546**	**NA**	**4230**	**6630**	**2910**	**1360000**	**18700**	**9420**	**9680**	**9030**	**7280**	**9310**	**9160**	**12100**	**4340**	**7560**	**5050**	
GM Sum Nuclear Foci Int per Cell (Cell) - GFP-IMPs	NA	208	337	154	158000	1450	5100	5000	5240	1700	4640	1690	3310	2650	3660	1910	
GM Sum Nuclear Foci Int per Cell (Cell) - DRAQ5	NA	3790	9090	3840	1030000	14200	7200	7340	6970	11600	8470	9530	13100	4170	6080	5250	
GM Sum Cytoplasmic Foci Int per Cell (Cell) - SFPQ-A546	NA	606	NA	684	281	8450	1770	2080	1380	875	1240	1350	2260	1240	2730	467	
GM Sum Cytoplasmic Foci Int per Cell (Cell) - GFP-IMPs	NA	32.3	NA	38	14.4	382	714	858	539	229	328	267	456	260	789	169	
GM Sum Cytoplasmic Foci Int per Cell (Cell) - DRAQ5	NA	333	NA	738	195	6030	1070	1210	892	561	779	682	1470	442	1500	389	
**GM Nuclear Foci Volume**	**NA**	**0.183**	**0.297**	**0.195**	**1.51**	**1.38**	**0.318**	**0.324**	**0.308**	**0.329**	**0.317**	**0.286**	**0.32**	**0.202**	**0.264**	**0.264**	Per Foci
**GM Nuclear Foci Int - SFPQ-A546**	**NA**	**89.4**	**84.9**	**84**	**190**	**187**	**89.6**	**89.4**	**89.8**	**87.2**	**89**	**89.3**	**90.2**	**87.6**	**87.5**	**88.3**	
GM Nuclear Foci Int - GFP-IMPs	NA	4.32	4.48	4.35	11	10.7	50.9	49.4	53.6	19.2	45.2	16.3	23.4	46.9	40.8	33.6	
GM Nuclear Foci Int - DRAQ5	NA	79.7	110	108	139	138	67.4	66.6	68.8	140	79.7	92	91.5	80.9	68.3	92.2	
**GM Sum Nuclear Foci Int - SFPQ-A546**	**NA**	**669**	**956**	**599**	**12500**	**11200**	**1110**	**1080**	**1160**	**1200**	**1190**	**1070**	**1200**	**728**	**964**	**963**	
GM Sum Nuclear Foci Int - GFP-IMPs	NA	32.3	50.4	31	720	640	630	596	692	265	603	195	313	390	449	366	
GM Sum Nuclear Foci Int - DRAQ5	NA	596	1240	773	9150	8240	834	803	888	1920	1060	1100	1220	672	753	1010	
GM Cytoplasmic Foci Volume	NA	0.14	NA	0.157	0.0723	0.884	0.204	0.212	0.185	0.15	0.4	0.266	0.297	0.145	0.202	0.0958	
GM Cytoplasmic Foci Int - SFPQ-A546	NA	91.3	NA	81.1	81.2	166	89.6	89.7	89.5	88.5	132	86.1	89.1	96.5	88.3	91.2	
GM Cytoplasmic Foci Int - GFP-IMPs	NA	4.72	NA	4.2	4.15	5.68	38	40.6	31.9	26.7	9.5	12.1	27.1	10.2	21.6	58.9	
GM Cytoplasmic Foci Int - DRAQ5	NA	48	NA	99.1	56.3	115	49.6	47.6	55.2	63.7	88	44.1	60.4	47.1	46.6	73.7	
GM Sum Cytoplasmic Foci Int - SFPQ-A546	NA	500	NA	486	281	6300	687	686	688	482	2170	988	1120	499	723	277	
GM Sum Cytoplasmic Foci Int - GFP-IMPs	NA	25.9	NA	25.1	14.4	215	291	311	245	145	156	139	341	52.8	177	179	
GM Sum Cytoplasmic Foci Int - DRAQ5	NA	263	NA	594	195	4380	380	365	424	347	1450	506	760	244	382	224	

Outcomes are labelled (rows) as integers or geometric means (GM) for each experimental group (columns). Groups with the “ C” suffix are control groups and are copies of their compliment group without GFP-thresholding applied. The GFP-tagged importin transient transfection groups are all as indicated, while “GFP” group is a merge of the two GFP-alone groups “GFP-1” and “GFP-2”, which showed no obviously disenable differences. Other control groups included were not transfected cells “Not-Trans”, mock transfected cells (without plasmid DNA) “Mock-Trans” and secondary antibody only control images from all of the other experiment groups “ALL-GFP”.

Supplementary Table S7: Detailed outcomes of modulating IMPα expression and transport function on exogenous dsRed2-PSPC1-positive nuclear foci.

8C-C-PSPC1_V2.00	GFP-VOID	GFP-VOID-C	GFP	GFP-IMPa2-FL	GFP-IMPa2-IBB	GFP-IMPa2-ED	GFP-IMPa4-FL	GFP-IMPa4-IBB	GFP-IMPa6-FL	GFP-IMPa6-IBB	Section
SortOrder	3	3	7	10	11	12	13	14	15	16	Additional Limits
Nuclei PRE X, Y & Z Exclusions	457	457	364	1308	1206	1116	1587	1488	1290	1156	
CountExcludeImgBound.x	22	22	20	64	65	71	93	74	85	66	
CountExcludeImgBound.y	30	30	24	86	73	80	91	71	90	61	
CountExcludeImgBound.z	54	54	NA	102	630	22	7	480	36	549	
CountExcludeImgBound	101	101	43	240	706	165	182	587	203	613	
Nuclei POST X, Y & Z Exclusions	356	356	321	1068	500	951	1405	901	1087	543	
CountPreGFPLimit	533	533	453	1368	1315	1187	1638	1571	1342	1269	
CountPostGFPLimit	8	533	265	297	687	529	358	711	629	565	
Cells removed on IMP Limit	NA	NA	NA	NA	NA	NA	NA	NA	NA	NA	
Number of Cells (Nuclei)	5	356	180	228	241	446	317	386	513	219	Per Sample
Number Cells (Nuclei) +VE for Foci	4	165	50	117	92	195	189	143	195	87	
Number Cells (Nuclei) -VE for Foci	1	191	130	111	149	251	128	243	318	132	
**% Cells (Nuclei) Positive for Foci**	**80**	**46.3**	**27.8**	**51.3**	**38.2**	**43.7**	**59.6**	**37**	**38**	**39.7**	
Number of Nuclear Foci	163	19627	2508	3392	2147	6218	10853	4339	5473	3745	
Number of Cells (Cells)	5	356	180	228	241	446	317	386	513	219	
Number Cells (Cells) +VE cytoplasmic Foci	1	80	13	40	30	43	51	53	52	18	
Number of Cells (Cells) -VE cytoplasmic Foci	4	276	167	188	211	403	266	333	461	201	
% Cells (Cells) with cytoplasmic Foci	20	22.5	7.22	17.5	12.4	9.64	16.1	13.7	10.1	8.22	
Number of Cytoplasmic Foci	1	1019	66	270	141	123	204	179	183	81	
GM Cytoplasmic Int (Cells) - DsRed2-PSPC1	5.84	6.25	4.86	4.82	3.6	3.85	4.75	3.8	3.57	3.65	Per Cell (Sample Wide)
GM Cytoplasmic Int (Cells) - GFP-IMPs	3.92	4.04	15.5	7.43	9.1	5.83	6.13	7.27	8.91	9.03	
GM Cytoplasmic Int (Cells) - DRAQ5	13.1	16.9	11.2	11.6	11.7	16.3	14.1	12.1	15.1	11.7	
GM Nucleus Int (Nuclei) - DsRed2-PSPC1	20.7	11	8.07	8.95	5.84	6.16	9.2	6.78	5.57	6.79	
GM Nucleus Int (Nuclei) - GFP-IMPs	17.5	4.29	55	23.3	43.1	19.2	19.5	29.3	27.1	30.5	
GM Nucleus Int (Nuclei) - DRAQ5	59.3	37.7	40.4	39.6	42.4	50.7	46.7	40.2	55	43.1	
fNC Inf nCells (Count) - DsRed2-PSPC1	0	0	0	0	0	0	0	0	0	0	
fNC Inf nCells (Count) - GFP-IMPs	0	0	0	0	0	0	0	0	0	0	
fNC Inf nCells (Count) - DRAQ5	0	0	0	0	0	0	0	0	0	0	
**GM fNC per Cell (N&C) - DsRed2-PSPC1**	**3.54**	**1.76**	**1.66**	**1.86**	**1.62**	**1.6**	**1.94**	**1.78**	**1.56**	**1.86**	
GM fNC per Cell (N&C) - GFP-IMPs	4.45	1.06	3.55	3.13	4.73	3.29	3.17	4.03	3.04	3.38	
GM fNC per Cell (N&C) - DRAQ5	4.52	2.23	3.6	3.42	3.63	3.1	3.31	3.32	3.63	3.69	
**GM Int per Cell (C&N) - DsRed2-PSPC1**	**15.3**	**8.91**	**5.78**	**5.85**	**4.28**	**5.19**	**6.38**	**4.95**	**4.4**	**4.59**	
GM Int per Cell (C&N) - GFP-IMPs	12.6	4.15	24.5	11.1	18.1	13	10.6	14.3	15.7	14.5	
GM Int per Cell (C&N) - DRAQ5	41.9	26	18.4	18.2	19.7	34.5	25.1	21.2	29.9	19.8	
**GM number of nuclear Foci per cell (Nuclei)**	**28.1**	**31.1**	**12.7**	**11.5**	**7.49**	**10.3**	**14.9**	**7.78**	**9.04**	**15.6**	Per Cell (Foci +VE)
GM number of nuclear Foci per cell (Cell)	28.1	31.1	12.7	11.5	7.49	10.3	14.9	7.78	9.04	15.6	
GM number of cytoplasmic Foci per cell (Cell)	1	4.56	2.91	3.91	2.73	1.95	2.86	2.32	2.21	2.41	
**GM Sum volume of nuclear Foci per cell (Cell)**	**11.4**	**6.11**	**2.15**	**1.88**	**1.03**	**1.43**	**2.48**	**1**	**1.29**	**2.59**	
GM Sum volume of cytoplasmic Foci per cell (Cell)	0.0686	0.733	0.55	0.586	0.321	0.247	0.376	0.312	0.276	0.328	
**GM Sum Nuclear Foci Int per Cell (Cell) - DsRed2-PSPC1**	**66500**	**26500**	**8720**	**7700**	**3920**	**5500**	**10200**	**3840**	**5100**	**10400**	
GM Sum Nuclear Foci Int per Cell (Cell) - GFP-IMPs	42500	1440	17500	3240	5400	2470	4260	3250	5430	10500	
GM Sum Nuclear Foci Int per Cell (Cell) - DRAQ5	96700	14400	6870	5160	3080	5040	8320	2930	5340	8580	
GM Sum Cytoplasmic Foci Int per Cell (Cell) - DsRed2-PSPC1	62	3100	2370	2510	1200	964	1410	1220	1100	1260	
GM Sum Cytoplasmic Foci Int per Cell (Cell) - GFP-IMPs	6	199	2250	1010	704	145	364	342	1030	856	
GM Sum Cytoplasmic Foci Int per Cell (Cell) - DRAQ5	11	1240	1050	1000	561	435	609	480	578	573	
**GM Nuclear Foci Volume**	**0.432**	**0.259**	**0.215**	**0.162**	**0.159**	**0.155**	**0.229**	**0.179**	**0.197**	**0.18**	Per Foci
**GM Nuclear Foci Int - DsRed2-PSPC1**	**145**	**108**	**99.9**	**95.2**	**97.1**	**92.4**	**103**	**95.5**	**102**	**97.1**	
GM Nuclear Foci Int - GFP-IMPs	72.6	5.06	211	67	191	79.4	76.2	178	165	136	
GM Nuclear Foci Int - DRAQ5	167	53.6	76.1	63.8	79	94.4	87.7	73	116	83.8	
**GM Sum Nuclear Foci Int - DsRed2-PSPC1**	**2670**	**1160**	**885**	**613**	**613**	**577**	**970**	**694**	**820**	**707**	
GM Sum Nuclear Foci Int - GFP-IMPs	1340	54.5	1870	432	1210	495	718	1290	1320	991	
GM Sum Nuclear Foci Int - DRAQ5	3080	578	674	411	499	589	827	530	932	610	
GM Cytoplasmic Foci Volume	0.0686	0.17	0.193	0.13	0.153	0.118	0.134	0.141	0.128	0.171	
GM Cytoplasmic Foci Int - DsRed2-PSPC1	62	102	105	98.7	105	99	95	101	99.5	100	
GM Cytoplasmic Foci Int - GFP-IMPs	6	5.62	110	45	78.2	14.9	25.1	23.4	89.4	95.7	
GM Cytoplasmic Foci Int - DRAQ5	11	36.4	40.7	40.1	40.8	40.5	36.1	35.7	46.7	57.7	
GM Sum Cytoplasmic Foci Int - DsRed2-PSPC1	62	712	846	513	621	444	503	561	509	680	
GM Sum Cytoplasmic Foci Int - GFP-IMPs	6	39.2	885	234	461	67	133	131	457	649	
GM Sum Cytoplasmic Foci Int - DRAQ5	11	254	327	208	240	182	191	199	239	391	

Outcomes are labelled (rows) as integers or geometric means (GM) for each experimental group (columns). Groups with the “ C” suffix are control groups and are copies of their compliment group without GFP-thresholding applied. The GFP-tagged importin transient transfection groups are all as indicated, these plus the GFP-alone group “GFP” were all co-transfected with DsRed2-PSPC1 while the “GFP-VOID” group was transfected with only the DsRed2-PSPC1 construct.

Supplementary Table S8: Detailed outcomes of siRNA knockdown of IMPα2 on endogenous SFPQ-positive nuclear foci (EXP#1).

8C1-siRNA-IMPa2-SFPQ_V2.00	ALL-CTRLS	NT	MT	siRNA-IMPa2-10	siRNA-IMPa2-25	siRNA-SC-10	siRNA-SC-25	
SortOrder	0	2	5	17	18	21	22	Additional Limits
Nuclei PRE X, Y & Z Exclusions	640	839	456	1245	687	1063	644	
CountExcludeImgBound.x	60	87	36	105	50	108	49	
CountExcludeImgBound.y	62	86	41	107	61	104	58	
CountExcludeImgBound.z	0	0	0	0	0	0	0	
CountExcludeImgBound	122	161	76	205	108	206	101	
Nuclei POST X, Y & Z Exclusions	518	678	380	1040	579	857	543	
Cells removed on GFP Limit	NA	NA	NA	NA	NA	NA	NA	
Cells removed on IMP Limit	NA	NA	NA	NA	NA	NA	NA	
Number of Cells (Nuclei)	518	678	380	1040	579	857	543	Per Sample
Number Cells (Nuclei) +VE for Foci	2	537	327	514	500	733	490	
Number Cells (Nuclei) -VE for Foci	516	141	53	526	79	124	53	
% Cells (Nuclei) Positive for Foci	0.39	79.20	86.05	49.42	86.36	85.53	90.24	
Number of Nuclear Foci	2	2075	1663	5611	2444	3354	3219	
Number of Cells (Cells)	456	630	356	594	551	779	518	
Number Cells (Cells) +VE cytoplasmic Foci	2	27	39	37	69	37	71	
Number of Cells (Cells) -VE cytoplasmic Foci	454	603	317	557	482	742	447	
% Cells (Cells) with cytoplasmic Foci	0.439	4.29	11	6.23	12.5	4.75	13.7	
Number of Cytoplasmic Foci	2	81	98	88	152	89	197	
GM Cytoplasmic Int (Cells) - SFPQ-A488	1.88	3.09	3.84	2.85	5.08	3.55	4.07	Per Cell (Sample Wide)
GM Cytoplasmic Int (Cells) - DAPI	17.2	21.2	18.8	47.1	19.1	21.8	16	
GM Cytoplasmic Int (Cells) - IMPα2-A546	29.1	92.1	92.7	67.6	57.1	98.1	91.8	
GM Nucleus Int (Nuclei) - SFPQ-A488	1.98	28.6	41	8.19	46	35.3	44.6	
GM Nucleus Int (Nuclei) - DAPI	119	125	121	94.2	122	128	120	
GM Nucleus Int (Nuclei) - IMPα2-A546	27.5	149	156	60.2	98.4	155	159	
fNC Inf nCells (Count) - SFPQ-A488	NA	NA	NA	3	NA	NA	1	
fNC Inf nCells (Count) - DAPI	NA	NA	NA	3	NA	NA	1	
fNC Inf nCells (Count) - IMPα2-A546	NA	NA	NA	3	NA	NA	1	
GM fNC per Cell (N&C) - SFPQ-A488	1.05	9.24	10.7	2.88	9.05	9.94	11	
GM fNC per Cell (N&C) - DAPI	6.95	5.91	6.45	1.98	6.38	5.88	7.46	
GM fNC per Cell (N&C) - IMPα2-A546	0.946	1.61	1.68	0.885	1.72	1.58	1.73	
GM Int per Cell (C&N) - SFPQ-A488	1.93	11.3	13.3	12.5	19.4	14.1	14.6	
GM Int per Cell (C&N) - DAPI	72.2	54.1	44.4	79	54.5	56.3	42.7	
GM Int per Cell (C&N) - IMPα2-A546	28.3	110	108	75.9	71.4	117	109	
GM number of nuclear Foci per cell (Nuclei)	1	3.17	4.01	8.77	3.99	3.55	4.98	Per Cell (Foci +VE)
GM number of nuclear Foci per cell (Cell)	1	3.25	4.14	8.59	4.12	3.7	5.2	
GM number of cytoplasmic Foci per cell (Cell)	1	2.23	1.73	1.71	1.59	1.61	1.84	
GM Sum volume of nuclear Foci per cell (Cell)	1.05	1.58	1.71	3.43	1.52	1.58	2.01	
GM Sum volume of cytoplasmic Foci per cell (Cell)	0.963	0.828	0.611	0.555	0.653	0.654	0.695	
GM Sum Nuclear Foci Int per Cell (Cell) - SFPQ-A488	27100	27700	34600	73800	32800	31200	41900	
GM Sum Nuclear Foci Int per Cell (Cell) - DAPI	12300	26400	25700	77300	23300	27900	29100	
GM Sum Nuclear Foci Int per Cell (Cell) - IMPα2-A546	22100	46800	50000	97700	33500	47300	62000	
GM Sum Cytoplasmic Foci Int per Cell (Cell) - SFPQ-A488	19300	11700	10100	7900	10800	9330	11800	
GM Sum Cytoplasmic Foci Int per Cell (Cell) - DAPI	3490	3690	3840	4740	4030	3680	4040	
GM Sum Cytoplasmic Foci Int per Cell (Cell) - IMPα2-A546	13400	24900	15300	15500	11400	19000	20500	
GM Nuclear Foci Volume	1.05	0.401	0.32	0.301	0.286	0.325	0.291	Per Foci
GM Nuclear Foci Int - SFPQ-A488	199	136	163	168	170	155	166	
GM Nuclear Foci Int - DAPI	90.5	119	110	159	111	126	105	
GM Nuclear Foci Int - IMPα2-A546	163	218	222	208	165	223	233	
GM Sum Nuclear Foci Int - SFPQ-A488	27100	7160	6850	6620	6370	6620	6310	
GM Sum Nuclear Foci Int - DAPI	12300	6280	4620	6280	4150	5390	4000	
GM Sum Nuclear Foci Int - IMPα2-A546	22100	11500	9330	8200	6180	9510	8870	
GM Cytoplasmic Foci Volume	0.963	0.294	0.304	0.312	0.361	0.303	0.297	
GM Cytoplasmic Foci Int - SFPQ-A488	157	110	138	114	120	114	134	
GM Cytoplasmic Foci Int - DAPI	28.5	28.2	45.5	63.9	37.5	31.6	35.9	
GM Cytoplasmic Foci Int - IMPα2-A546	110	222	204	235	135	239	219	
GM Sum Cytoplasmic Foci Int - SFPQ-A488	19300	4300	5370	4490	5650	4480	5180	
GM Sum Cytoplasmic Foci Int - DAPI	3490	1100	1770	2510	1770	1240	1380	
GM Sum Cytoplasmic Foci Int - IMPα2-A546	13400	8690	7920	9250	6380	9410	8460	

Outcomes are labelled (rows) as integers or geometric means (GM) for each experimental group (columns). The IMPα2 siRNAs were used at 10 and 25 nM final concentrations (“IMPα2-10” and “IMPα2-25” groups), with matched scrambled siRNA controls, also used at 10 and 25 nM final concentrations (“SC-10” and “SC-25” groups). Additional control groups included not transfected cells “NT”, mock transfected cells “MT” and secondary antibody control images from all the other groups “ALL-CTRLS”.

Supplementary Table S9: Detailed outcomes of siRNA knockdown of IMPα2 on endogenous PSPC1-positive nuclear foci (EXP#1).

8C1-siRNA-IMPa2-PSPC1_V2.00	ALL-CTRLS	NT	MT	siRNA-IMPa2-10	siRNA-IMPa2-25	siRNA-SC-10	siRNA-SC-25	
SortOrder	0	2	5	17	18	21	22	Additional Limits
Nuclei PRE X, Y & Z Exclusions	574	519	582	400	726	590	1297	
CountExcludeImgBound.x	62	59	59	32	70	44	105	
CountExcludeImgBound.y	52	49	53	30	65	60	132	
CountExcludeImgBound.z	NA	NA	NA	NA	NA	2	NA	
CountExcludeImgBound	110	103	111	62	133	104	232	
Nuclei POST X, Y & Z Exclusions	464	416	471	338	593	486	1065	
Cells removed on GFP Limit	NA	NA	NA	NA	NA	NA	NA	
Cells removed on IMP Limit	NA	NA	NA	NA	NA	NA	NA	
Number of Cells (Nuclei)	464	416	471	338	593	486	1065	Per Sample
Number Cells (Nuclei) +VE for Foci	3	398	438	329	559	479	988	
Number Cells (Nuclei) -VE for Foci	461	18	33	9	34	7	77	
% Cells (Nuclei) Positive for Foci	0.65	95.67	92.99	97.34	94.27	98.56	92.77	
Number of Nuclear Foci	4	4651	3187	12508	5071	24236	8768	
Number of Cells (Cells)	391	391	452	327	558	459	967	
Number Cells (Cells) +VE cytoplasmic Foci	22	184	167	315	222	437	227	
Number of Cells (Cells) -VE cytoplasmic Foci	369	207	285	12	336	22	740	
% Cells (Cells) with cytoplasmic Foci	5.63	47.1	36.9	96.3	39.8	95.2	23.5	
Number of Cytoplasmic Foci	50	945	625	5616	830	6590	490	
GM Cytoplasmic Int (Cells) - PSPC1-A488	8.8	8.99	8.86	17.7	12.7	16.6	10.4	Per Cell (Sample Wide)
GM Cytoplasmic Int (Cells) - DAPI	20.3	20.7	18.1	12.9	15	17.1	8.25	
GM Cytoplasmic Int (Cells) - IMPα2-A546	28.4	82.3	86.7	84.1	54.9	105	92	
GM Nucleus Int (Nuclei) - PSPC1-A488	9.29	46.1	48.9	85.9	52.6	88.5	54.1	
GM Nucleus Int (Nuclei) - DAPI	116	116	113	99.4	113	107	135	
GM Nucleus Int (Nuclei) - IMPα2-A546	23.4	123	141	122	78.3	151	144	
fNC Inf nCells (Count) - PSPC1-A488	NA	NA	NA	NA	NA	1	NA	
fNC Inf nCells (Count) - DAPI	NA	NA	NA	NA	NA	1	NA	
fNC Inf nCells (Count) - IMPα2-A546	NA	NA	NA	NA	NA	1	NA	
GM fNC per Cell (N&C) - PSPC1-A488	1.06	5.12	5.52	4.86	4.13	5.35	5.2	
GM fNC per Cell (N&C) - DAPI	5.69	5.62	6.26	7.73	7.51	6.19	16.3	
GM fNC per Cell (N&C) - IMPα2-A546	0.825	1.49	1.62	1.45	1.42	1.43	1.56	
GM Int per Cell (C&N) - PSPC1-A488	9.04	19	19.1	33.7	26.5	33.2	26.2	
GM Int per Cell (C&N) - DAPI	69.8	46.3	42.3	32.9	48.4	37.7	53.3	
GM Int per Cell (C&N) - IMPα2-A546	25.9	93.2	100	93.2	63.4	116	110	
GM number of nuclear Foci per cell (Nuclei)	1.26	9.57	5.74	24.6	6.84	38.1	7.05	Per Cell (Foci +VE)
GM number of nuclear Foci per cell (Cell)	1.19	10.1	5.83	25.5	7.18	41.1	7.56	
GM number of cytoplasmic Foci per cell (Cell)	1.74	3.43	2.5	9.05	2.33	8.62	1.65	
GM Sum volume of nuclear Foci per cell (Cell)	0.473	3.11	1.52	4.79	1.88	9.01	1.69	
GM Sum volume of cytoplasmic Foci per cell (Cell)	1	0.833	0.613	2.76	0.606	3.05	0.393	
GM Sum Nuclear Foci Int per Cell (Cell) - PSPC1-A488	6000	68300	34200	131000	44800	240000	41800	
GM Sum Nuclear Foci Int per Cell (Cell) - DAPI	3430	39000	18600	45100	24400	94700	28000	
GM Sum Nuclear Foci Int per Cell (Cell) - IMPα2-A546	4040	84900	41900	144000	32700	289000	49200	
GM Sum Cytoplasmic Foci Int per Cell (Cell) - PSPC1-A488	9490	16900	12800	48100	11600	45100	8190	
GM Sum Cytoplasmic Foci Int per Cell (Cell) - DAPI	3170	5780	4050	10100	3060	11200	2200	
GM Sum Cytoplasmic Foci Int per Cell (Cell) - IMPα2-A546	5750	19000	14800	73500	8190	85000	9080	
GM Nuclear Foci Volume	0.433	0.219	0.191	0.145	0.189	0.158	0.166	Per Foci
GM Nuclear Foci Int - PSPC1-A488	103	173	177	199	184	201	187	
GM Nuclear Foci Int - DAPI	57.6	89.1	88.8	69	88.6	77.6	114	
GM Nuclear Foci Int - IMPα2-A546	73.4	203	205	219	125	237	211	
GM Sum Nuclear Foci Int - PSPC1-A488	5780	5000	4490	3830	4610	4190	4150	
GM Sum Nuclear Foci Int - DAPI	3250	2570	2250	1320	2220	1620	2520	
GM Sum Nuclear Foci Int - IMPα2-A546	4140	5860	5200	4200	3130	4950	4680	
GM Cytoplasmic Foci Volume	0.483	0.189	0.182	0.184	0.197	0.215	0.207	
GM Cytoplasmic Foci Int - PSPC1-A488	81.5	166	162	166	163	144	166	
GM Cytoplasmic Foci Int - DAPI	23.9	51.7	45.9	31.7	37.9	31.5	42.3	
GM Cytoplasmic Foci Int - IMPα2-A546	51.5	169	172	194	94.7	216	171	
GM Sum Cytoplasmic Foci Int - PSPC1-A488	5150	4230	3950	4030	4240	4070	4520	
GM Sum Cytoplasmic Foci Int - DAPI	1510	1310	1120	768	988	889	1150	
GM Sum Cytoplasmic Foci Int - IMPα2-A546	3250	4300	4180	4700	2470	6070	4670	

Outcomes are labelled (rows) as integers or geometric means (GM) for each experimental group (columns). The IMPα2 siRNAs were used at 10 and 25 nM final concentrations (“IMPα2-10” and “IMPα2-25” groups), with matched scrambled siRNA controls, also used at 10 and 25 nM final concentrations (“SC-10” and “SC-25” groups). Additional control groups included not transfected cells “NT”, mock transfected cells “MT” and secondary antibody control images from all the other groups “ALL-CTRLS”.

Supplementary Table S10: Detailed outcomes of siRNA knockdown of IMPα4 on endogenous SFPQ-positive nuclear foci (EXP#1).

8C1-siRNA-IMPa4-SFPQ_V2.00	ALL-CTRLS	NT	MT	siRNA-IMPa4-10	siRNA-IMPa4-25	siRNA-SC-10	siRNA-SC-25	
SortOrder	0	2	5	19	20	21	22	Additional Limits
Nuclei PRE X, Y & Z Exclusions	1072	880	630	357	545	553	483	
CountExcludeImgBound.x	89	70	75	39	53	52	48	
CountExcludeImgBound.y	99	87	62	36	46	55	36	
CountExcludeImgBound.z	1	3	NA	NA	NA	14	NA	
CountExcludeImgBound	180	156	130	70	97	117	82	
Nuclei POST X, Y & Z Exclusions	892	724	500	287	448	436	401	
Cells removed on GFP Limit	NA	NA	NA	NA	NA	NA	NA	
Cells removed on IMP Limit	NA	NA	NA	NA	NA	NA	NA	
Number of Cells (Nuclei)	892	724	500	287	448	436	401	Per Sample
Number Cells (Nuclei) +VE for Foci	NA	530	280	263	392	375	355	
Number Cells (Nuclei) -VE for Foci	892	194	220	24	56	61	46	
% Cells (Nuclei) Positive for Foci	NA	73.20	56.00	91.64	87.50	86.01	88.53	
Number of Nuclear Foci	0	1766	1141	1589	2128	2076	1998	
Number of Cells (Cells)	762	666	474	277	415	416	389	
Number Cells (Cells) +VE cytoplasmic Foci	NA	22	7	10	49	38	82	
Number of Cells (Cells) -VE cytoplasmic Foci	762	644	467	267	366	378	307	
% Cells (Cells) with cytoplasmic Foci	NA	3.3	1.48	3.61	11.8	9.13	21.1	
Number of Cytoplasmic Foci	0	39	9	11	89	84	216	
GM Cytoplasmic Int (Cells) - SFPQ-A488	1.52	2.8	2.61	3.41	4.36	4.09	4.97	Per Cell (Sample Wide)
GM Cytoplasmic Int (Cells) - DAPI	20	31.5	25.8	19.6	19.4	18.2	12.1	
GM Cytoplasmic Int (Cells) - IMPα4-A546	45.9	53.3	63.4	57.1	59	64	57	
GM Nucleus Int (Nuclei) - SFPQ-A488	1.76	23.4	26.8	42.6	39.1	39.5	41.5	
GM Nucleus Int (Nuclei) - DAPI	118	135	130	134	113	127	103	
GM Nucleus Int (Nuclei) - IMPα4-A546	44.9	70.3	90.3	90.2	80.4	90.6	83.5	
fNC Inf nCells (Count) - SFPQ-A488	1	NA	NA	NA	NA	NA	NA	
fNC Inf nCells (Count) - DAPI	1	NA	NA	NA	NA	NA	NA	
fNC Inf nCells (Count) - IMPα4-A546	1	NA	NA	NA	NA	NA	NA	
GM fNC per Cell (N&C) - SFPQ-A488	1.16	8.36	10.3	12.5	8.99	9.65	8.36	
GM fNC per Cell (N&C) - DAPI	5.86	4.29	5.04	6.84	5.83	7	8.49	
GM fNC per Cell (N&C) - IMPα4-A546	0.974	1.32	1.42	1.58	1.36	1.41	1.46	
GM Int per Cell (C&N) - SFPQ-A488	1.62	11.9	10.8	16.5	15.3	15.2	15.5	
GM Int per Cell (C&N) - DAPI	59.7	75.7	61.1	57.7	48.7	52	37.8	
GM Int per Cell (C&N) - IMPα4-A546	46.1	61.1	72.7	68.4	66	72.7	64.8	
GM number of nuclear Foci per cell (Nuclei)	NA	2.67	2.95	4.92	4.13	4.23	4.37	Per Cell (Foci +VE)
GM number of nuclear Foci per cell (Cell)	NA	2.72	2.93	4.97	4.27	4.33	4.5	
GM number of cytoplasmic Foci per cell (Cell)	NA	1.47	1.17	1.07	1.54	1.78	1.81	
GM Sum volume of nuclear Foci per cell (Cell)	NA	1.4	1.39	1.92	2.01	1.87	1.92	
GM Sum volume of cytoplasmic Foci per cell (Cell)	NA	0.566	0.438	0.462	0.61	0.624	0.609	
GM Sum Nuclear Foci Int per Cell (Cell) - SFPQ-A488	NA	21700	25000	40000	37800	36700	37700	
GM Sum Nuclear Foci Int per Cell (Cell) - DAPI	NA	26200	24800	36100	27500	30500	23300	
GM Sum Nuclear Foci Int per Cell (Cell) - IMPα4-A546	NA	22900	25800	40600	36600	37700	36500	
GM Sum Cytoplasmic Foci Int per Cell (Cell) - SFPQ-A488	NA	7660	6410	6330	9570	8710	9930	
GM Sum Cytoplasmic Foci Int per Cell (Cell) - DAPI	NA	3950	3320	2640	3590	2910	3010	
GM Sum Cytoplasmic Foci Int per Cell (Cell) - IMPα4-A546	NA	12200	6770	10900	10000	10800	10000	
GM Nuclear Foci Volume	NA	0.444	0.382	0.291	0.357	0.337	0.321	Per Foci
GM Nuclear Foci Int - SFPQ-A488	NA	122	146	165	150	158	157	
GM Nuclear Foci Int - DAPI	NA	139	128	135	99.7	117	88.4	
GM Nuclear Foci Int - IMPα4-A546	NA	126	140	159	141	152	146	
GM Sum Nuclear Foci Int - SFPQ-A488	NA	7100	7290	6340	6990	6970	6610	
GM Sum Nuclear Foci Int - DAPI	NA	8100	6370	5210	4640	5150	3730	
GM Sum Nuclear Foci Int - IMPα4-A546	NA	7320	6970	6110	6550	6710	6170	
GM Cytoplasmic Foci Volume	NA	0.324	0.362	0.438	0.413	0.271	0.318	
GM Cytoplasmic Foci Int - SFPQ-A488	NA	120	115	101	126	123	134	
GM Cytoplasmic Foci Int - DAPI	NA	47.4	55	41.4	44.5	32.2	34.5	
GM Cytoplasmic Foci Int - IMPα4-A546	NA	140	125	182	128	151	134	
GM Sum Cytoplasmic Foci Int - SFPQ-A488	NA	5120	5550	5880	6690	4460	5600	
GM Sum Cytoplasmic Foci Int - DAPI	NA	2020	2670	2410	2360	1160	1440	
GM Sum Cytoplasmic Foci Int - IMPα4-A546	NA	5990	6070	10600	6760	5460	5590	

Outcomes are labelled (rows) as integers or geometric means (GM) for each experimental group (columns). The IMPα4 siRNAs were used at 10 and 25 nM final concentrations (“IMPα4-10” and “IMPα4-25” groups), with matched scrambled siRNA controls, also used at 10 and 25 nM final concentrations (“SC-10” and “SC-25” groups). Additional control groups included not transfected cells “NT”, mock transfected cells “MT” and secondary antibody control images from all the other groups “ALL-CTRLS”.

Supplementary Table S11: Detailed outcomes of siRNA knockdown of IMPα4 on endogenous PSPC1-positive nuclear foci (EXP#1).

8C1-siRNA-IMPa4-PSPC1_V2.00	ALL-CTRLS	NT	MT	siRNA-IMPa4-10	siRNA-IMPa4-25	siRNA-SC-10	siRNA-SC-25	
SortOrder	0	2	5	19	20	21	22	Additional Limits
Nuclei PRE X, Y & Z Exclusions	822	651	911	616	952	750	666	
CountExcludeImgBound.x	67	69	86	67	86	67	65	
CountExcludeImgBound.y	76	74	85	66	87	77	70	
CountExcludeImgBound.z	5	69	NA	NA	2	1	NA	
CountExcludeImgBound	146	185	165	128	172	143	124	
Nuclei POST X, Y & Z Exclusions	676	466	746	488	780	607	542	
Cells removed on GFP Limit	NA	NA	NA	NA	NA	NA	NA	
Cells removed on IMP Limit	NA	NA	NA	NA	NA	NA	NA	
Number of Cells (Nuclei)	676	466	746	488	780	607	542	Per Sample
Number Cells (Nuclei) +VE for Foci	45	451	705	443	735	590	516	
Number Cells (Nuclei) -VE for Foci	631	15	41	45	45	17	26	
% Cells (Nuclei) Positive for Foci	6.66	96.78	94.50	90.78	94.23	97.20	95.20	
Number of Nuclear Foci	130	5372	8711	3710	14101	13847	7172	
Number of Cells (Cells)	578	437	705	459	715	575	514	
Number Cells (Cells) +VE cytoplasmic Foci	111	140	290	319	465	391	211	
Number of Cells (Cells) -VE cytoplasmic Foci	467	297	415	140	250	184	303	
% Cells (Cells) with cytoplasmic Foci	19.2	32	41.1	69.5	65	68	41.1	
Number of Cytoplasmic Foci	393	441	1167	1538	2520	2775	788	
GM Cytoplasmic Int (Cells) - PSPC1-A488	6.08	9.48	12.1	13.8	17.1	14.5	12.7	Per Cell (Sample Wide)
GM Cytoplasmic Int (Cells) - DAPI	19.7	27.1	19	15.7	16	15.7	21.9	
GM Cytoplasmic Int (Cells) - IMPα4-A546	50.1	51.5	57.5	62.6	76.1	66.1	59	
GM Nucleus Int (Nuclei) - PSPC1-A488	9.32	50.2	63.5	61.5	84.1	74.3	72	
GM Nucleus Int (Nuclei) - DAPI	118	131	118	100	117	105	124	
GM Nucleus Int (Nuclei) - IMPα4-A546	46.2	61.8	72.1	74.5	90.8	89	82.8	
fNC Inf nCells (Count) - PSPC1-A488	NA	NA	1	NA	NA	NA	NA	
fNC Inf nCells (Count) - DAPI	NA	NA	1	NA	NA	NA	NA	
fNC Inf nCells (Count) - IMPα4-A546	NA	NA	1	NA	NA	NA	NA	
GM fNC per Cell (N&C) - PSPC1-A488	1.53	5.29	5.23	4.47	4.92	5.12	5.67	
GM fNC per Cell (N&C) - DAPI	6	4.85	6.18	6.4	7.3	6.72	5.65	
GM fNC per Cell (N&C) - IMPα4-A546	0.921	1.2	1.25	1.19	1.19	1.35	1.4	
GM Int per Cell (C&N) - PSPC1-A488	7.23	24.7	29.7	25.8	38.7	31.2	32.1	
GM Int per Cell (C&N) - DAPI	54.1	66	52.5	37	47.9	40.7	55.3	
GM Int per Cell (C&N) - IMPα4-A546	49.1	55.7	63.1	65.9	81.3	73	67.1	
GM number of nuclear Foci per cell (Nuclei)	2.02	9.51	9.76	6.45	13.7	15.4	10.8	Per Cell (Foci +VE)
GM number of nuclear Foci per cell (Cell)	2	9.96	10	6.61	14.7	16.3	11.3	
GM number of cytoplasmic Foci per cell (Cell)	2.25	2.13	2.68	3.26	3.27	4.12	2.51	
GM Sum volume of nuclear Foci per cell (Cell)	0.751	2.85	2.22	1.6	2.72	3.64	2.29	
GM Sum volume of cytoplasmic Foci per cell (Cell)	1.06	0.508	0.575	0.802	0.695	1.01	0.57	
GM Sum Nuclear Foci Int per Cell (Cell) - PSPC1-A488	8530	63800	53800	38500	71600	92200	57200	
GM Sum Nuclear Foci Int per Cell (Cell) - DAPI	6910	44000	29100	16700	32600	41100	31700	
GM Sum Nuclear Foci Int per Cell (Cell) - IMPα4-A546	10900	37600	36700	26200	61900	79500	44700	
GM Sum Cytoplasmic Foci Int per Cell (Cell) - PSPC1-A488	9250	9800	12200	16200	13400	19600	10600	
GM Sum Cytoplasmic Foci Int per Cell (Cell) - DAPI	3280	3620	3530	4270	3330	5000	3450	
GM Sum Cytoplasmic Foci Int per Cell (Cell) - IMPα4-A546	12200	5490	8710	12100	14200	19300	8940	
GM Nuclear Foci Volume	0.334	0.201	0.168	0.182	0.153	0.173	0.158	Per Foci
GM Nuclear Foci Int - PSPC1-A488	107	175	184	186	194	189	187	
GM Nuclear Foci Int - DAPI	71.5	109	90	77.8	84.7	82.7	95.8	
GM Nuclear Foci Int - IMPα4-A546	116	94.7	117	122	159	164	137	
GM Sum Nuclear Foci Int - PSPC1-A488	4690	4650	4100	4490	3940	4340	3950	
GM Sum Nuclear Foci Int - DAPI	3120	2880	2010	1870	1720	1900	2030	
GM Sum Nuclear Foci Int - IMPα4-A546	5050	2520	2620	2950	3240	3760	2900	
GM Cytoplasmic Foci Volume	0.354	0.188	0.153	0.176	0.169	0.178	0.169	
GM Cytoplasmic Foci Int - PSPC1-A488	88.2	156	172	166	160	164	159	
GM Cytoplasmic Foci Int - DAPI	23	50.7	44.4	39.3	37.2	36.8	46	
GM Cytoplasmic Foci Int - IMPα4-A546	91.4	92.1	110	109	149	139	119	
GM Sum Cytoplasmic Foci Int - PSPC1-A488	4070	3870	3550	3900	3610	3870	3530	
GM Sum Cytoplasmic Foci Int - DAPI	1060	1250	917	920	837	868	1020	
GM Sum Cytoplasmic Foci Int - IMPα4-A546	4220	2280	2280	2560	3350	3270	2650	

Outcomes are labelled (rows) as integers or geometric means (GM) for each experimental group (columns). The IMPα4 siRNAs were used at 10 and 25 nM final concentrations (“IMPα4-10” and “IMPα4-25” groups), with matched scrambled siRNA controls, also used at 10 and 25 nM final concentrations (“SC-10” and “SC-25” groups). Additional control groups included not transfected cells “NT”, mock transfected cells “MT” and secondary antibody control images from all the other groups “ALL-CTRLS”.

Supplementary Table S12: Detailed outcomes of siRNA knockdown of IMPα2 on endogenous SFPQ-positive nuclear foci (EXP#2).

8C2-siRNA-IMPa2-SFPQ_V2.00	ALL-CTRLS	NT	MT	siRNA-IMPa2-10	siRNA-IMPa2-25	siRNA-SC-10	siRNA-SC-25	Group
SortOrder	0	2	5	17	18	21	22	Additional Limits
Nuclei PRE X, Y & Z Exclusions	1263	2052	1004	761	810	1563	1280	
CountExcludeImgBound.x	141	217	121	80	84	145	97	
CountExcludeImgBound.y	137	214	97	74	94	154	137	
CountExcludeImgBound.z	13	2	5	NA	NA	6	7	
CountExcludeImgBound	279	418	213	147	171	294	234	
Nuclei POST X, Y & Z Exclusions	984	1634	791	614	639	1269	1046	
Cells removed on GFP Limit	NA	NA	NA	NA	NA	NA	NA	
Cells removed on IMP Limit	NA	NA	NA	NA	NA	NA	NA	
Number of Cells (Nuclei)	984	1634	791	614	639	1269	1046	Per Sample
Number Cells (Nuclei) +VE for Foci	NA	189	267	364	342	446	468	
Number Cells (Nuclei) -VE for Foci	984	1445	524	250	297	823	578	
% Cells (Nuclei) Positive for Foci	NA	11.57	33.75	59.28	53.52	35.15	44.74	
Number of Nuclear Foci	0	263	469	1040	959	797	1051	
Number of Cells (Cells)	734	1300	724	588	545	969	940	
Number Cells (Cells) +VE cytoplasmic Foci	NA	8	16	40	21	58	43	
Number of Cells (Cells) -VE cytoplasmic Foci	734	1292	708	548	524	911	897	
% Cells (Cells) with cytoplasmic Foci	NA	0.615	2.21	6.8	3.85	5.99	4.57	
Number of Cytoplasmic Foci	0	10	20	63	39	83	66	
GM Cytoplasmic Int (Cells) - SFPQ-A488	1.81	2.71	3.1	4.35	4.18	4.71	3.94	Per Cell (Sample Wide)
GM Cytoplasmic Int (Cells) - DAPI	13	18.2	11.4	8.51	7.86	8.83	9.42	
GM Cytoplasmic Int (Cells) - IMPα2-A546	31.7	104	98.5	73	88.7	124	105	
GM Nucleus Int (Nuclei) - SFPQ-A488	1.59	15.3	21.8	30.5	26.6	25	26.8	
GM Nucleus Int (Nuclei) - DAPI	90.8	101	88.6	77.3	92.1	76.9	81.2	
GM Nucleus Int (Nuclei) - IMPα2-A546	23.4	124	134	102	111	152	140	
fNC Inf nCells (Count) - SFPQ-A488	0	0	0	0	0	0	0	
fNC Inf nCells (Count) - DAPI	0	0	0	0	0	0	0	
fNC Inf nCells (Count) - IMPα2-A546	0	0	0	0	0	0	0	
GM fNC per Cell (N&C) - SFPQ-A488	0.881	5.64	7.04	7	6.37	5.3	6.8	
GM fNC per Cell (N&C) - DAPI	7	5.56	7.81	9.09	11.7	8.7	8.62	
GM fNC per Cell (N&C) - IMPα2-A546	0.739	1.19	1.36	1.4	1.25	1.22	1.33	
GM Int per Cell (C&N) - SFPQ-A488	1.66	8.88	8.96	12	13.1	11.9	11.4	
GM Int per Cell (C&N) - DAPI	66.2	56.9	35.3	28.5	40.3	32.2	32.2	
GM Int per Cell (C&N) - IMPα2-A546	26.1	114	110	81.3	98	135	117	
GM number of nuclear Foci per cell (Nuclei)	NA	1.26	1.51	2.22	2.2	1.52	1.83	Per Cell (Foci +VE)
GM number of nuclear Foci per cell (Cell)	NA	1.3	1.54	2.25	2.35	1.62	1.88	
GM number of cytoplasmic Foci per cell (Cell)	NA	1.19	1.17	1.33	1.6	1.26	1.34	
GM Sum volume of nuclear Foci per cell (Cell)	NA	0.71	0.838	1.02	0.984	0.827	0.899	
GM Sum volume of cytoplasmic Foci per cell (Cell)	NA	0.55	0.437	0.416	0.535	0.543	0.562	
GM Sum Nuclear Foci Int per Cell (Cell) - SFPQ-A488	NA	9640	12400	18300	18300	12900	14800	
GM Sum Nuclear Foci Int per Cell (Cell) - DAPI	NA	11000	9090	9480	12600	7410	8690	
GM Sum Nuclear Foci Int per Cell (Cell) - IMPα2-A546	NA	19800	23700	22600	24100	24900	25800	
GM Sum Cytoplasmic Foci Int per Cell (Cell) - SFPQ-A488	NA	5490	6250	6130	8010	7710	7510	
GM Sum Cytoplasmic Foci Int per Cell (Cell) - DAPI	NA	1690	1890	1470	1800	1810	1730	
GM Sum Cytoplasmic Foci Int per Cell (Cell) - IMPα2-A546	NA	17900	10200	7460	13300	15300	12900	
GM Nuclear Foci Volume	NA	0.508	0.501	0.368	0.343	0.458	0.404	Per Foci
GM Nuclear Foci Int - SFPQ-A488	NA	105	114	146	148	122	130	
GM Nuclear Foci Int - DAPI	NA	117	82	69.5	95.6	67.4	70	
GM Nuclear Foci Int - IMPα2-A546	NA	213	213	173	189	228	215	
GM Sum Nuclear Foci Int - SFPQ-A488	NA	7020	7590	7050	6690	7390	6880	
GM Sum Nuclear Foci Int - DAPI	NA	7820	5470	3350	4310	4070	3710	
GM Sum Nuclear Foci Int - IMPα2-A546	NA	14300	14200	8350	8530	13800	11400	
GM Cytoplasmic Foci Volume	NA	0.409	0.361	0.328	0.332	0.397	0.396	
GM Cytoplasmic Foci Int - SFPQ-A488	NA	79	106	116	108	108	103	
GM Cytoplasmic Foci Int - DAPI	NA	22.3	34.5	24.2	19.9	21.4	22.1	
GM Cytoplasmic Foci Int - IMPα2-A546	NA	243	168	134	186	220	172	
GM Sum Cytoplasmic Foci Int - SFPQ-A488	NA	4430	5280	4880	4740	5700	5220	
GM Sum Cytoplasmic Foci Int - DAPI	NA	1250	1710	1020	877	1130	1120	
GM Sum Cytoplasmic Foci Int - IMPα2-A546	NA	13600	8350	5620	8190	11600	8770	

Outcomes are labelled (rows) as integers or geometric means (GM) for each experimental group (columns). The IMPα2 siRNAs were used at 10 and 25 nM final concentrations (“IMPα2-10” and “IMPα2-25” groups), with matched scrambled siRNA controls, also used at 10 and 25 nM final concentrations (“SC-10” and “SC-25” groups). Additional control groups included not transfected cells “NT”, mock transfected cells “MT” and secondary antibody control images from all the other groups “ALL-CTRLS”.

Supplementary Table S13: Detailed outcomes of siRNA knockdown of IMPα2 on endogenous SFPQ-positive nuclear foci (EXP#2).

8C2-siRNA-IMPa2-PSPC1_V2.00	ALL-CTRLS	NT	MT	siRNA-IMPa2-10	siRNA-IMPa2-25	siRNA-SC-10	siRNA-SC-25	
SortOrder	0	2	5	17	18	21	22	Additional Limits
Nuclei PRE X, Y & Z Exclusions	1021	1172	894	905	612	531	886	
CountExcludeImgBound.x	77	104	89	75	64	57	85	
CountExcludeImgBound.y	104	125	105	103	64	64	95	
CountExcludeImgBound.z	178	2	NA	NA	111	NA	22	
CountExcludeImgBound	332	222	188	174	211	115	190	
Nuclei POST X, Y & Z Exclusions	689	950	706	731	401	416	696	
Cells removed on GFP Limit	NA	NA	NA	NA	NA	NA	NA	
Cells removed on IMP Limit	NA	NA	NA	NA	NA	NA	NA	
Number of Cells (Nuclei)	689	950	706	731	401	416	696	Per Sample
Number Cells (Nuclei) +VE for Foci	1	814	642	678	373	395	635	
Number Cells (Nuclei) -VE for Foci	688	136	64	53	28	21	61	
% Cells (Nuclei) Positive for Foci	0.15	85.68	90.93	92.75	93.02	94.95	91.24	
Number of Nuclear Foci	1	6249	5016	5905	3343	3482	5405	
Number of Cells (Cells)	623	824	633	661	352	397	638	
Number Cells (Cells) +VE cytoplasmic Foci	NA	54	53	163	67	173	131	
Number of Cells (Cells) -VE cytoplasmic Foci	623	770	580	498	285	224	507	
% Cells (Cells) with cytoplasmic Foci	NA	6.55	8.37	24.7	19	43.6	20.5	
Number of Cytoplasmic Foci	0	84	93	409	144	578	300	
GM Cytoplasmic Int (Cells) - PSPC1-A488	5.64	6.3	8.01	12	11.4	10.2	8.87	Per Cell (Sample Wide)
GM Cytoplasmic Int (Cells) - DAPI	23.2	29.7	25.9	21.7	23.8	12.4	18.3	
GM Cytoplasmic Int (Cells) - IMPα2-A546	16.8	65.2	69.6	56.5	42	72	70.4	
GM Nucleus Int (Nuclei) - PSPC1-A488	4.26	29.5	39.8	45.6	45.3	53.9	43.3	
GM Nucleus Int (Nuclei) - DAPI	118	107	112	91.9	119	86.4	100	
GM Nucleus Int (Nuclei) - IMPα2-A546	10.3	79.6	92.5	66	50.3	109	94.2	
fNC Inf nCells (Count) - PSPC1-A488	58	NA	NA	NA	NA	NA	NA	
fNC Inf nCells (Count) - DAPI	58	NA	NA	NA	NA	NA	NA	
fNC Inf nCells (Count) - IMPα2-A546	58	NA	NA	NA	NA	NA	NA	
GM fNC per Cell (N&C) - PSPC1-A488	0.666	4.69	4.97	3.79	3.96	5.3	4.89	
GM fNC per Cell (N&C) - DAPI	3.77	3.6	4.31	4.22	4.99	6.95	5.46	
GM fNC per Cell (N&C) - IMPα2-A546	0.484	1.22	1.33	1.17	1.2	1.51	1.34	
GM Int per Cell (C&N) - PSPC1-A488	4.41	16.7	21.5	26.6	28.4	23.3	22.1	
GM Int per Cell (C&N) - DAPI	110	64	62.1	52	71	34.7	49.2	
GM Int per Cell (C&N) - IMPα2-A546	10.9	72.4	79.8	61.1	46.5	83.3	80	
GM number of nuclear Foci per cell (Nuclei)	1	5.92	6.11	6.49	6.91	6.63	6.57	Per Cell (Foci +VE)
GM number of nuclear Foci per cell (Cell)	1	6.26	6.55	6.92	7.77	6.76	6.88	
GM number of cytoplasmic Foci per cell (Cell)	NA	1.37	1.48	1.86	1.62	2.19	1.66	
GM Sum volume of nuclear Foci per cell (Cell)	0.809	2.21	1.89	1.82	2.27	1.57	2.01	
GM Sum volume of cytoplasmic Foci per cell (Cell)	NA	0.19	0.354	0.371	0.383	0.417	0.386	
GM Sum Nuclear Foci Int per Cell (Cell) - PSPC1-A488	6480	43000	41900	41600	50200	38000	44800	
GM Sum Nuclear Foci Int per Cell (Cell) - DAPI	5450	31200	28900	19800	31500	15300	23900	
GM Sum Nuclear Foci Int per Cell (Cell) - IMPα2-A546	2510	41600	40000	28100	25700	37900	42600	
GM Sum Cytoplasmic Foci Int per Cell (Cell) - PSPC1-A488	NA	2610	6910	7890	7720	9410	7990	
GM Sum Cytoplasmic Foci Int per Cell (Cell) - DAPI	NA	1040	2170	1930	2250	1720	1930	
GM Sum Cytoplasmic Foci Int per Cell (Cell) - IMPα2-A546	NA	1930	5010	4260	3370	7430	5650	
GM Nuclear Foci Volume	0.809	0.238	0.196	0.192	0.204	0.177	0.205	Per Foci
GM Nuclear Foci Int - PSPC1-A488	57.9	159	175	179	175	185	176	
GM Nuclear Foci Int - DAPI	48.7	99.6	106	76.3	95.9	68.2	84.4	
GM Nuclear Foci Int - IMPα2-A546	22.4	135	149	110	77.8	172	151	
GM Sum Nuclear Foci Int - PSPC1-A488	6480	4950	4520	4490	4690	4350	4740	
GM Sum Nuclear Foci Int - DAPI	5450	3100	2730	1920	2570	1600	2280	
GM Sum Nuclear Foci Int - IMPα2-A546	2510	4220	3850	2760	2080	4050	4080	
GM Cytoplasmic Foci Volume	NA	0.14	0.212	0.178	0.2	0.154	0.196	
GM Cytoplasmic Foci Int - PSPC1-A488	NA	126	136	169	155	183	155	
GM Cytoplasmic Foci Int - DAPI	NA	46.7	43	38.9	41.6	31.1	36.6	
GM Cytoplasmic Foci Int - IMPα2-A546	NA	85.1	101	82.4	59.3	132	108	
GM Sum Cytoplasmic Foci Int - PSPC1-A488	NA	2170	3810	3950	4020	3710	3850	
GM Sum Cytoplasmic Foci Int - DAPI	NA	805	1200	908	1080	631	913	
GM Sum Cytoplasmic Foci Int - IMPα2-A546	NA	1470	2830	1920	1540	2680	2680	

Outcomes are labelled (rows) as integers or geometric means (GM) for each experimental group (columns). The IMPα2 siRNAs were used at 10 and 25 nM final concentrations (“IMPα2-10” and “IMPα2-25” groups), with matched scrambled siRNA controls, also used at 10 and 25 nM final concentrations (“SC-10” and “SC-25” groups). Additional control groups included not transfected cells “NT”, mock transfected cells “MT” and secondary antibody control images from all the other groups “ALL-CTRLS”.

Supplementary Table S14: Detailed outcomes of siRNA knockdown of IMPα4 on endogenous SFPQ-positive nuclear foci (EXP#2).

8C2-siRNA-IMPa4-SFPQ_V2.00	ALL-CTRLS	NT	MT	siRNA-IMPa4-10	siRNA-IMPa4-25	siRNA-SC-10	siRNA-SC-25	
SortOrder	0	2	5	19	20	21	22	Additional Limits
Nuclei PRE X, Y & Z Exclusions	1028	1078	1108	636	708	779	616	
CountExcludeImgBound.x	89	96	95	40	73	65	47	
CountExcludeImgBound.y	93	125	101	60	66	76	47	
CountExcludeImgBound.z	28	258	NA	3	NA	31	NA	
CountExcludeImgBound	202	436	189	100	130	163	94	
Nuclei POST X, Y & Z Exclusions	826	642	919	536	578	616	522	
Cells removed on GFP Limit	NA	NA	NA	NA	NA	NA	NA	
Cells removed on IMP Limit	NA	NA	NA	NA	NA	NA	NA	
Number of Cells (Nuclei)	826	642	919	536	578	616	522	Per Sample
Number Cells (Nuclei) +VE for Foci	NA	24	263	147	205	68	171	
Number Cells (Nuclei) -VE for Foci	826	618	656	389	373	548	351	
% Cells (Nuclei) Positive for Foci	NA	3.74	28.62	27.43	35.47	11.04	32.76	
Number of Nuclear Foci	0	28	451	253	432	90	320	
Number of Cells (Cells)	666	576	847	515	555	567	507	
Number Cells (Cells) +VE cytoplasmic Foci	NA	2	50	61	54	6	73	
Number of Cells (Cells) -VE cytoplasmic Foci	666	574	797	454	501	561	434	
% Cells (Cells) with cytoplasmic Foci	NA	0.347	5.9	11.8	9.73	1.06	14.4	
Number of Cytoplasmic Foci	0	3	62	78	81	8	130	
GM Cytoplasmic Int (Cells) - SFPQ-A488	1.35	3.53	4.97	5.52	5.02	3.2	5.2	Per Cell (Sample Wide)
GM Cytoplasmic Int (Cells) - DAPI	9.38	14.1	9.96	6.56	6.04	8.55	7.48	
GM Cytoplasmic Int (Cells) - IMPα4-A546	40	47.2	60.6	56.2	55	60.1	55.9	
GM Nucleus Int (Nuclei) - SFPQ-A488	1.37	15.9	22.7	24	26.4	16.3	26.5	
GM Nucleus Int (Nuclei) - DAPI	67.4	75.6	61.7	52.2	58.9	73	51.9	
GM Nucleus Int (Nuclei) - IMPα4-A546	27.3	48.4	62.9	60.8	63.8	71	64.6	
fNC Inf nCells (Count) - SFPQ-A488	0	0	0	0	0	0	0	
fNC Inf nCells (Count) - DAPI	0	0	0	0	0	0	0	
fNC Inf nCells (Count) - IMPα4-A546	0	0	0	0	0	0	0	
GM fNC per Cell (N&C) - SFPQ-A488	1.01	4.5	4.57	4.34	5.25	5.09	5.1	
GM fNC per Cell (N&C) - DAPI	7.18	5.38	6.2	7.96	9.76	8.53	6.93	
GM fNC per Cell (N&C) - IMPα4-A546	0.682	1.03	1.04	1.08	1.16	1.18	1.16	
GM Int per Cell (C&N) - SFPQ-A488	1.36	8.77	11.2	10.7	11.7	7.77	10.5	
GM Int per Cell (C&N) - DAPI	33.5	39.9	27.8	19	22.1	30.9	18.4	
GM Int per Cell (C&N) - IMPα4-A546	35	48.1	62	57.8	58.1	64.4	58.4	
GM number of nuclear Foci per cell (Nuclei)	NA	1.12	1.48	1.48	1.74	1.21	1.61	Per Cell (Foci +VE)
GM number of nuclear Foci per cell (Cell)	NA	1.12	1.51	1.48	1.74	1.21	1.63	
GM number of cytoplasmic Foci per cell (Cell)	NA	1.41	1.14	1.17	1.32	1.2	1.47	
GM Sum volume of nuclear Foci per cell (Cell)	NA	0.635	0.806	0.775	0.889	0.719	0.842	
GM Sum volume of cytoplasmic Foci per cell (Cell)	NA	0.867	0.491	0.63	0.584	0.566	0.741	
GM Sum Nuclear Foci Int per Cell (Cell) - SFPQ-A488	NA	7490	12200	11900	14100	10100	13800	
GM Sum Nuclear Foci Int per Cell (Cell) - DAPI	NA	5910	5770	4500	6100	5930	5120	
GM Sum Nuclear Foci Int per Cell (Cell) - IMPα4-A546	NA	7990	12600	12300	14300	11000	13500	
GM Sum Cytoplasmic Foci Int per Cell (Cell) - SFPQ-A488	NA	8740	6960	8490	8020	7530	10800	
GM Sum Cytoplasmic Foci Int per Cell (Cell) - DAPI	NA	4980	1790	1870	1750	2450	2300	
GM Sum Cytoplasmic Foci Int per Cell (Cell) - IMPα4-A546	NA	19300	7170	9030	8140	7130	10300	
GM Nuclear Foci Volume	NA	0.551	0.485	0.463	0.449	0.551	0.468	Per Foci
GM Nuclear Foci Int - SFPQ-A488	NA	88.9	119	118	128	111	129	
GM Nuclear Foci Int - DAPI	NA	68.1	56	44.2	54.9	63.3	46.2	
GM Nuclear Foci Int - IMPα4-A546	NA	94.1	119	120	126	117	123	
GM Sum Nuclear Foci Int - SFPQ-A488	NA	6580	7670	7240	7610	8050	7950	
GM Sum Nuclear Foci Int - DAPI	NA	5030	3610	2710	3250	4610	2840	
GM Sum Nuclear Foci Int - IMPα4-A546	NA	6960	7680	7330	7500	8530	7580	
GM Cytoplasmic Foci Volume	NA	0.62	0.441	0.489	0.419	0.456	0.467	
GM Cytoplasmic Foci Int - SFPQ-A488	NA	71.9	111	97	110	97.7	115	
GM Cytoplasmic Foci Int - DAPI	NA	29.1	26.6	20	22.9	31.3	22.4	
GM Cytoplasmic Foci Int - IMPα4-A546	NA	166	113	100	107	96.9	105	
GM Sum Cytoplasmic Foci Int - SFPQ-A488	NA	6000	6430	6210	6030	6040	7010	
GM Sum Cytoplasmic Foci Int - DAPI	NA	2420	1540	1280	1250	1940	1370	
GM Sum Cytoplasmic Foci Int - IMPα4-A546	NA	13800	6550	6430	5880	5990	6420	

Outcomes are labelled (rows) as integers or geometric means (GM) for each experimental group (columns). The IMPα4 siRNAs were used at 10 and 25 nM final concentrations (“IMPα4-10” and “IMPα4-25” groups), with matched scrambled siRNA controls, also used at 10 and 25 nM final concentrations (“SC-10” and “SC-25” groups). Additional control groups included not transfected cells “NT”, mock transfected cells “MT” and secondary antibody control images from all the other groups “ALL-CTRLS”.

Supplementary Table S15: Detailed outcomes of siRNA knockdown of IMPα4 on endogenous PSPC1-positive nuclear foci (EXP#2).

8C2-siRNA-IMPa4-PSPC1_V2.00	ALL-CTRLS	NT	MT	siRNA-IMPa4-10	siRNA-IMPa4-25	siRNA-SC-10	siRNA-SC-25	
SortOrder	0	2	5	19	20	21	22	Additional Limits
Nuclei PRE X, Y & Z Exclusions	1391	1043	1089	1647	968	1435	631	
CountExcludeImgBound.x	167	94	90	132	89	151	60	
CountExcludeImgBound.y	151	97	113	142	106	154	67	
CountExcludeImgBound.z	NA	NA	NA	1	NA	NA	NA	
CountExcludeImgBound	287	182	197	266	186	297	123	
Nuclei POST X, Y & Z Exclusions	1104	861	892	1381	782	1138	508	
Cells removed on GFP Limit	NA	NA	NA	NA	NA	NA	NA	
Cells removed on IMP Limit	NA	NA	NA	NA	NA	NA	NA	
Number of Cells (Nuclei)	1104	861	892	1381	782	1138	508	Per Sample
Number Cells (Nuclei) +VE for Foci	10	784	817	1168	696	1040	470	
Number Cells (Nuclei) -VE for Foci	1094	77	75	213	86	98	38	
% Cells (Nuclei) Positive for Foci	0.91	91.06	91.59	84.58	89.00	91.39	92.52	
Number of Nuclear Foci	11	10829	8899	9097	7360	12871	4323	
Number of Cells (Cells)	864	751	828	1020	722	1020	481	
Number Cells (Cells) +VE cytoplasmic Foci	10	157	137	323	160	58	146	
Number of Cells (Cells) -VE cytoplasmic Foci	854	594	691	698	562	961	335	
% Cells (Cells) with cytoplasmic Foci	1.16	20.9	16.5	31.6	22.2	5.69	30.4	
Number of Cytoplasmic Foci	16	345	323	765	394	116	467	
GM Cytoplasmic Int (Cells) - PSPC1-A488	6.03	10.1	11.8	15.7	15	9.36	12	Per Cell (Sample Wide)
GM Cytoplasmic Int (Cells) - DAPI	12.2	14.1	14.2	10.5	11.5	21.6	10.2	
GM Cytoplasmic Int (Cells) - IMPα4-A546	53.4	62.3	64.3	79.4	81.1	63.3	65.4	
GM Nucleus Int (Nuclei) - PSPC1-A488	6.75	52	65.2	60.2	70.8	51.4	69.3	
GM Nucleus Int (Nuclei) - DAPI	93.2	94	103	79.6	101	114	85.2	
GM Nucleus Int (Nuclei) - IMPα4-A546	44.7	69.7	84.7	90.1	106	77.8	91	
fNC Inf nCells (Count) - PSPC1-A488	0	0	0	0	0	0	0	
fNC Inf nCells (Count) - DAPI	0	0	0	0	0	0	0	
fNC Inf nCells (Count) - IMPα4-A546	0	0	0	0	0	0	0	
GM fNC per Cell (N&C) - PSPC1-A488	1.12	5.15	5.53	3.84	4.74	5.49	5.76	
GM fNC per Cell (N&C) - DAPI	7.65	6.69	7.28	7.57	8.74	5.27	8.35	
GM fNC per Cell (N&C) - IMPα4-A546	0.837	1.12	1.32	1.14	1.3	1.23	1.39	
GM Int per Cell (C&N) - PSPC1-A488	6.42	29.8	34.9	38.3	36.9	31.7	31.3	
GM Int per Cell (C&N) - DAPI	50.4	50.9	52.8	45.1	46.4	69.8	35.6	
GM Int per Cell (C&N) - IMPα4-A546	50.3	67	73.5	86	91.2	72.1	74.7	
GM number of nuclear Foci per cell (Nuclei)	1.07	10.9	8.39	5.99	7.84	9.74	6.92	Per Cell (Foci +VE)
GM number of nuclear Foci per cell (Cell)	1.07	11.9	8.71	7.35	8.19	10.3	7.17	
GM number of cytoplasmic Foci per cell (Cell)	1.28	1.7	1.77	1.77	1.74	1.53	2.08	
GM Sum volume of nuclear Foci per cell (Cell)	0.578	2.57	1.7	1.41	1.34	2.1	1.29	
GM Sum volume of cytoplasmic Foci per cell (Cell)	0.764	0.405	0.341	0.306	0.406	0.361	0.365	
GM Sum Nuclear Foci Int per Cell (Cell) - PSPC1-A488	5050	61500	42900	35300	34700	51300	32600	
GM Sum Nuclear Foci Int per Cell (Cell) - DAPI	3600	32700	21900	14400	17100	35400	12600	
GM Sum Nuclear Foci Int per Cell (Cell) - IMPα4-A546	5460	40300	30800	27100	31100	37700	24800	
GM Sum Cytoplasmic Foci Int per Cell (Cell) - PSPC1-A488	8430	7970	6670	6560	7670	6650	8100	
GM Sum Cytoplasmic Foci Int per Cell (Cell) - DAPI	1880	1890	1550	1280	1460	2000	1500	
GM Sum Cytoplasmic Foci Int per Cell (Cell) - IMPα4-A546	10500	4760	4640	4820	7510	5810	5730	
GM Nuclear Foci Volume	0.538	0.158	0.15	0.151	0.134	0.154	0.141	Per Foci
GM Nuclear Foci Int - PSPC1-A488	66.3	182	187	189	192	183	188	
GM Nuclear Foci Int - DAPI	46.2	86.9	87.4	70.2	85.9	118	65.9	
GM Nuclear Foci Int - IMPα4-A546	73.2	111	127	140	163	127	136	
GM Sum Nuclear Foci Int - PSPC1-A488	4770	3830	3770	3810	3450	3760	3560	
GM Sum Nuclear Foci Int - DAPI	3330	1830	1760	1420	1540	2420	1250	
GM Sum Nuclear Foci Int - IMPα4-A546	5270	2340	2550	2820	2940	2620	2560	
GM Cytoplasmic Foci Volume	0.398	0.182	0.163	0.146	0.162	0.205	0.15	
GM Cytoplasmic Foci Int - PSPC1-A488	93.7	156	164	168	155	145	172	
GM Cytoplasmic Foci Int - DAPI	12.6	35.6	35.6	31.4	25.6	42.1	28.4	
GM Cytoplasmic Foci Int - IMPα4-A546	91.7	87.4	110	119	133	132	114	
GM Sum Cytoplasmic Foci Int - PSPC1-A488	4830	3700	3520	3240	3300	4080	3430	
GM Sum Cytoplasmic Foci Int - DAPI	652	846	764	604	547	1180	566	
GM Sum Cytoplasmic Foci Int - IMPα4-A546	4730	2070	2370	2300	2840	3710	2270	

Outcomes are labelled (rows) as integers or geometric means (GM) for each experimental group (columns). The IMPα4 siRNAs were used at 10 and 25 nM final concentrations (“IMPα4-10” and “IMPα4-25” groups), with matched scrambled siRNA controls, also used at 10 and 25 nM final concentrations (“SC-10” and “SC-25” groups). Additional control groups included not transfected cells “NT”, mock transfected cells “MT” and secondary antibody control images from all the other groups “ALL-CTRLS”.

Supplementary Figure S1: SFPQ nuclear accumulation and cellular IMPα levels in HeLa cells.

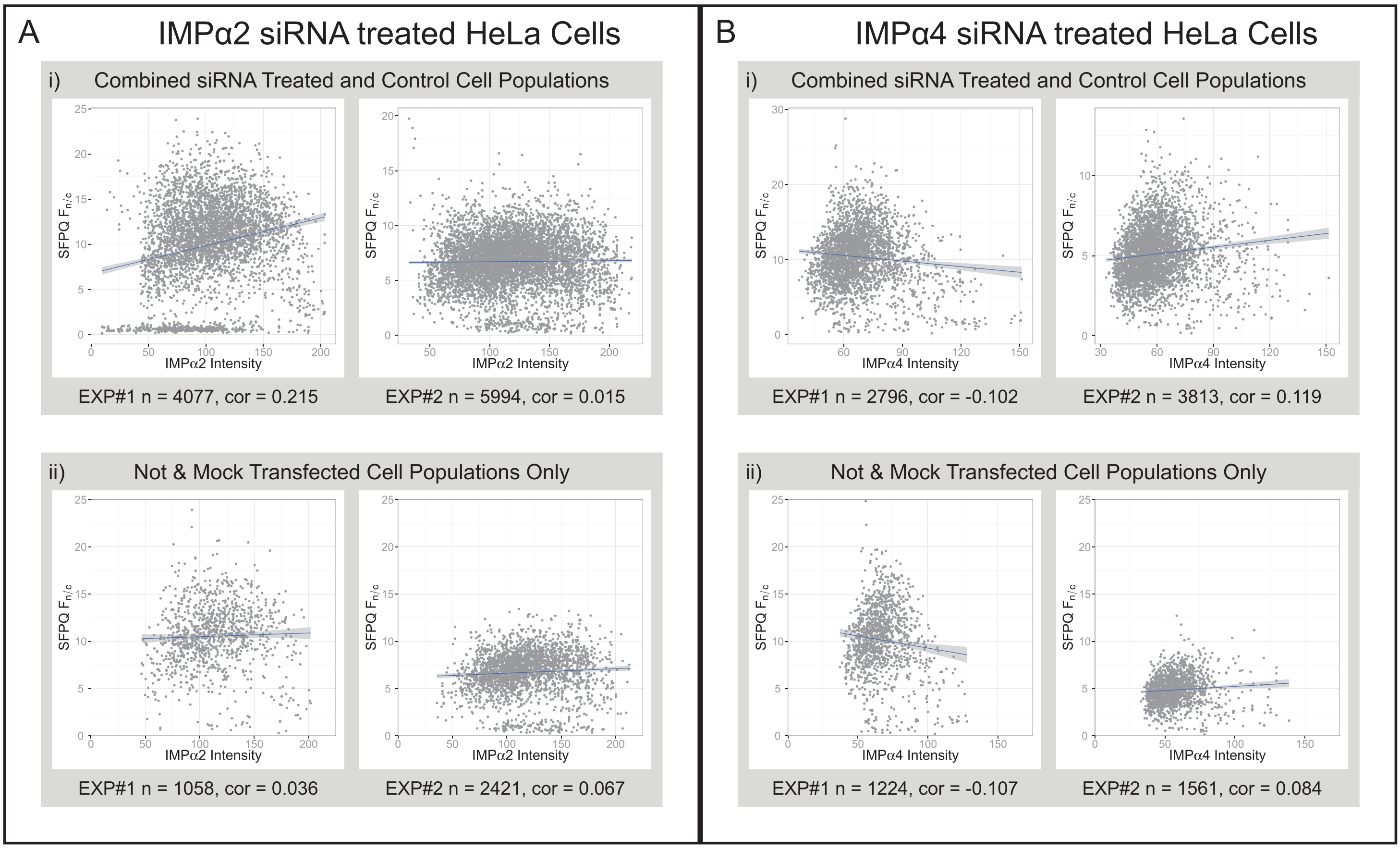

Population wide correlations were observed, where treatment groups (Not transfected, mock transfected, scrambled-10, scrambled-25, IMPα-10, IMPα-25) within two independent experiments (EXP#1 and EXP#2) were pooled and the total number of cells (n) were used to produce correlation coefficients (c) between cellular IMPα intensity and the ratio of SFPQ nuclear to cytoplasmic intensity ($F_{n/c}$).
